# Effect of pH on the denitrification proteome of the soil bacterium *Paracoccus denitrificans* PD1222

**DOI:** 10.1038/s41598-021-96559-2

**Published:** 2021-08-26

**Authors:** Alfonso Olaya-Abril, Jesús Hidalgo-Carrillo, Víctor M. Luque-Almagro, Carlos Fuentes-Almagro, Francisco J. Urbano, Conrado Moreno-Vivián, David J. Richardson, María Dolores Roldán

**Affiliations:** 1 Departamento de Bioquímica y Biología Molecular, Universidad de Córdoba, Edificio Severo Ochoa, 1ª planta, Campus de Rabanales, 14071 Córdoba, Spain; 2 Departamento de Química Orgánica, Universidad de Córdoba, Edificio Marie Curie, Campus de Rabanales, 14071 Córdoba, Spain; 3 Servicio Central de Apoyo a la Investigación (SCAI), Unidad de Proteómica, Universidad de Córdoba, Campus de Rabanales, 14071 Córdoba, Spain; 4 School of Biological Sciences, University of East Anglia, Norwich Research Park, Norwich, NR4 7TJ UK

**Keywords:** Microbiology, Environmental sciences

## Abstract

Denitrification is a respiratory process by which nitrate is reduced to dinitrogen. Incomplete denitrification results in the emission of the greenhouse gas nitrous oxide and this is potentiated in acidic soils, which display reduced denitrification rates and high N_2_O/N_2_ ratios compared to alkaline soils. In this work, impact of pH on the proteome of the soil denitrifying bacterium *Paracoccus denitrificans* PD1222 was analysed with nitrate as sole energy and nitrogen source under anaerobic conditions at pH ranging from 6.5 to 7.5. Quantitative proteomic analysis revealed that the highest difference in protein representation was observed when the proteome at pH 6.5 was compared to the reference proteome at pH 7.2. However, this difference in the extracellular pH was not enough to produce modification of intracellular pH, which was maintained at 6.5 ± 0.1. The biosynthetic pathways of several cofactors relevant for denitrification and nitrogen assimilation like cobalamin, riboflavin, molybdopterin and nicotinamide were negatively affected at pH 6.5. In addition, peptide representation of reductases involved in nitrate assimilation and denitrification were reduced at pH 6.5. Data highlight the strong negative impact of pH on NosZ synthesis and intracellular copper content, thus impairing active NosZ assembly and, in turn, leading to elevated nitrous oxide emissions.

## Introduction

A great variety of nitrogen cycle processes are highly active in soils, including denitrification, which contributes to 70% of global nitrous oxide emissions^1,2^. This pathway, carried out by different bacteria and fungi, comprises the sequential reduction of nitrate (NO_3_^−^) to nitrite (NO_2_^−^), nitric oxide (NO), nitrous oxide (N_2_O) and dinitrogen (N_2_). Under oxygen limiting conditions, each intermediate serve as respiratory electron acceptor enabling energy metabolism and growth^3,4^.

The model soil bacterium *Paracoccus denitrificans* performs the complete denitrification pathway, using a molybdenum-dependent membrane-bound nitrate reductase (Nar), a cytochrome *cd*_1_-type nitrite reductase (NirS), a heme *c/b* nitric oxide reductase (Nor), and a copper-dependent nitrous oxide reductase (NosZ). All these reductases can be coupled to the core electron transport pathway at the level of the ubiquinol pool in the membrane and the periplasmic heme-containing cytochrome *c*_550_ or Cu-containing pseudoazurin pool^3,5^. Denitrification is negatively controlled in response to oxygen by FnrP and positively regulated in response to nitric oxide by Nnr. In addition, NarR, NirI, and NosR specifically regulate expression of the *nar*, *nir*, and *nos* genes, respectively^5^.

Large quantities of nitrogen species are released into the environment because of the intense productivity levels in agricultural systems, mainly based on the conversion of ammonium into nitrite through nitrification and the inefficiency in the uptake of nitrogenous sources^6^. This fact has led to serious environmental problems, such as increased emissions of nitrous oxide, and health problems associated with the accumulation of nitrate and nitrite in water resources^7^.

Nitrous oxide is a potent greenhouse gas with a long atmospheric lifetime and a global warming potential about 300 times higher than carbon dioxide^8^. Additionally, it is a relevant molecule in the depletion of stratospheric ozone^9^. N_2_O emissions come from natural processes in oceans and soils, and from anthropogenic sources^10^. In crop soils, the highest N_2_O emissions occur following the application of fertilizers, especially with the cultivation of cereals^11^. Denitrification is not only favoured by excess of inorganic nitrogen, but also by low oxygen or carbon availability, high pH and elevated temperature and humidity that influence oxygen availability^12,13^. However, key processes leading to the passage through the soil of N-fertilizers, and formation, consumption and emission of N_2_O, are not fully understood in the natural context^14^.

Recent studies support that N_2_O emissions are potentiated in acidic soils. Thus, it has been described that a denitrifier community from farmed soil was impaired to reduce N_2_O to N_2_ at acidic pH, but this capability was reestablished at neutral pH^15^. In this sense, to reduce N_2_O emissions from soil denitrifiers, management techniques have been applied to increase soil pH, thus mitigating emissions of this greenhouse gas^16^. In another work, only about 20% of the total denitrifiers isolated from soils at controlled acidic and neutral pHs were described to perform complete denitrification. However, opposed to the commonly described inability to reduce N_2_O under acidic conditions, one isolated strain identified as *Rhodanobacter* reduced N_2_O only at low pH^17^.

Denitrification is conditioned by the need to regulate this process avoiding accumulation of toxic intermediaries, mainly nitric oxide^5,18^. A global understanding of the biochemical machinery and regulation involved in the microbial production and consumption of inorganic nitrogen could enhance application of soil management practices to increase the yield of N-source utilization and to reduce N_2_O emissions. In this sense, next-generation proteomic techniques face new challenges in environmental biotechnology^19^. Recently, a holistic quantitative proteomic analysis was applied to elucidate the denitrification proteome in the soil bacterium *Paracoccus denitrificans*^20^.

Soil pH has been described as a key factor affecting denitrification in a great variety of denitrifiers, especially at the level of nitrous oxide reduction^13–17^. The aim of this work was to study the influence of pH on the proteome of the model soil denitrifier *Paracoccus denitrificans* PD1222, identifying proteins affected by changes in the pH with a particular interest on denitrification proteins and their cofactors and how these changes may impact on the variations in N_2_O emissions.

## Results

### Effect of pH on *P. denitrificans* PD1222 growth under denitrifying conditions

**Figure 1:**
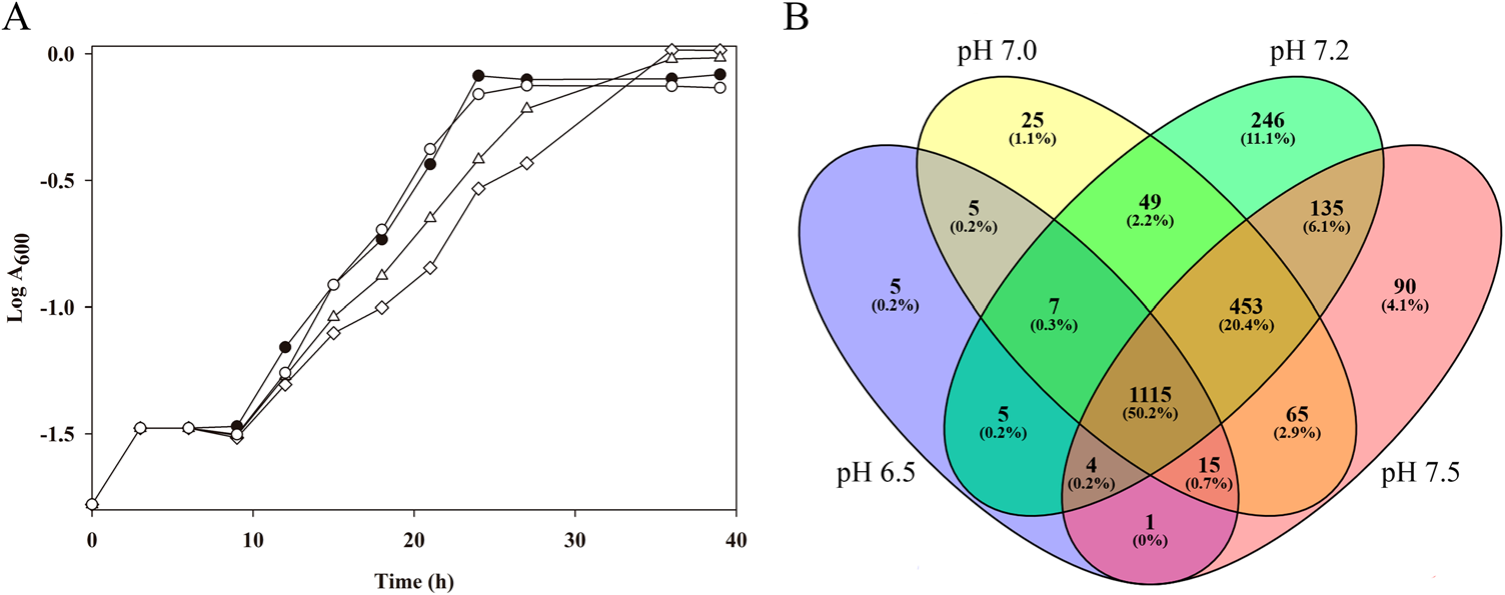
Effect of pH on growth and proteome of *P. denitrifcans* PD1222. Growth curves of *P. denitrificans* in mineral media with nitrate (30 mM) as nitrogen and energy source. Black circles, growth at pH 7.2; triangles, growth at pH 7.0; diamonds, growth at pH 6.5; white circles, growth at pH 7.5 (**A**). Proteomic analysis of the *P. denitrificans* cells grown at different pHs: blue, pH 6.5; yellow, pH 7.0; green, pH 7.2; red, pH 7.5. Number of identified proteins in each pH condition is indicated, and the percentage with respect to the total identified protein in all conditions is given in brackets (**B**). The Sigmaplot software for Windows version 11.0 (http://www.sigmaplot.co.uk) was used to create the growth curves and the Venny software version 2.1 (https://bioinfogp.cnb.csic.es/tools/venny/) was used to design the diagram.

To study the effect of pH on growth and the proteome of *P. denitrificans* PD1222, this strain was cultured in mineral salt media at different initial pHs (6.5, 7.0, 7.2 or 7.5) under anaerobic denitrifying conditions with nitrate (30 mM) as both nitrogen source and electron acceptor. *P. denitrificans* showed a lower growth rate at pH 6.5 compared to the optimal pH 7.2 (Fig. 1A), but nitrate consumption was similar in both conditions. Thus, at early exponential growth phase (A_600_ ⁓0.3) about 17–18 mM nitrate was consumed, and at late exponential phase (A_600_ ⁓0.9) nitrate was completely exhausted, and almost totally converted into dinitrogen, at both pHs (Table 1). Nitrite was accumulated in the media at low concentration (µM), independently of the initial pH. Nitrous oxide was not released at pH 7.2, but it was detected at µM (early exponential phase) or mM (late exponential phase) concentrations when media were adjusted to pH 6.5. The intracellular pH was determined in *P. denitrificans* cells when cultures reached an A_600_ ~ 0.3. Independently of the extracellular pH, the intracellular pH was maintained at a value of 6.5 ± 0.1.

**Table 1:** Effect of pH on nitrogenous compounds and gases detected during denitrification.

pHe	A_600_	Protein (µg ml^-1^)	NO_3_^─^ consumed (mM)	NO_2_^─^ accumulated (µM)	N_2_O released (mM)	N_2_ produced (mM)
7.2	0.32 ± 0.01	29.3 ± 1.6	18.1 ± 0.4	64.5 ± 8.2	0 ± 0	7.8 ± 0.8
7.2	0.89 ± 0.02	236.5 ± 12.6	30 ± 0	349.7 ± 22.5	0 ± 0	13.6 ± 0.9
6.5	0.32 ± 0.01	35.2 ± 2.4	17.3 ± 0.4	563.0 ± 84.9	0.25 ± 0.1	7.6 ± 0.8
6.5	0.94 ± 0.03	236.8 ± 5.4	30 ± 0	72.7 ± 8.8	2.8 ± 0.4	12.8 ± 0.7

Cells were grown in anaerobiosis with nitrate (30 mM) in minimal medium at different initial pH (6.5 or 7.2). Samples were taken at early (A_600_ ⁓ 0.3) or late (A_600_ ⁓ 0.9) exponential growth phase. Data are means ± SD of three independent biological replicates (n = 3). N-oxyanions and denitrification gasses were determined as indicated in experimental procedures. pHe: extracellular pH.

### Global changes in the denitrification proteome depending on the pH

*P. denitrificans* PD1222 was cultured under anaerobic denitrifying conditions at four different pHs (6.5, 7.0, 7.2 or 7.5) to perform a quantitative proteomic analysis by Liquid Chromatography-Mass Spectrometry/Mass Spectrometry (LC–MS/MS). The principal component analysis (PCA) and the clustering of these biological replicates are shown as supplementary material (Figs. S1 & S2). Total proteins identified were 1157 at pH 6.5, 1734 at pH 7.0, 2014 at pH 7.2 and 1878 at pH 7.5 (Fig. 1B). Thus, the highest number of proteins was identified at pH 7.2, with 2014 proteins from ~ 5100 putative structural genes present in the *P. denitrificans* genome (~ 40% representation of the total predicted gene products).

The protein profile obtained at pH 7.2 was used as the reference condition, and differential analyses were performed by comparison of the proteome at pH 7.2 with those at pH 6.5, 7.0 or 7.5. In these comparative proteomic analyses, the term ‘exclusive’ describes proteins with an absolute value of fold change higher than 100, whereas proteins shared at both analysed pHs, but nevertheless differentially represented with fold changes lower than 100, were considered ‘over-represented’ (positive fold change) or ‘down-represented’ (negative fold change) at the reference pH 7.2. The most drastic differences were observed when comparing the proteomes at pH 6.5 and 7.2, with a total of 922 proteins affected, of which 702 were exclusive to pH 7.2 and 10 were exclusive to pH 6.5. Another 158 proteins were over-represented at pH 7.2 (down-represented at pH 6.5) and 52 proteins were over-represented at pH 6.5 (down-represented at pH 7.2) (Table S1).

A total of 584 proteins were differentially represented when comparing the proteomes at pH 7.0 and pH 7.2. In this case, 262 proteins were exclusive to pH 7.2, 85 were exclusive to pH 7.0 and 237 were shared at both pHs, with 148 proteins over-represented at pH 7.2 and 89 over-represented at pH 7.0 (Table S2). When comparing the proteomes at pH 7.5 and 7.2, 637 proteins were differentially represented, with 209 proteins exclusive to pH 7.2, 127 exclusive to pH 7.5 and 301 shared between both pHs, of which 172 were over-represented at pH 7.2 and 129 were over-represented at pH 7.5 (Table S3).

**Figure 2:**
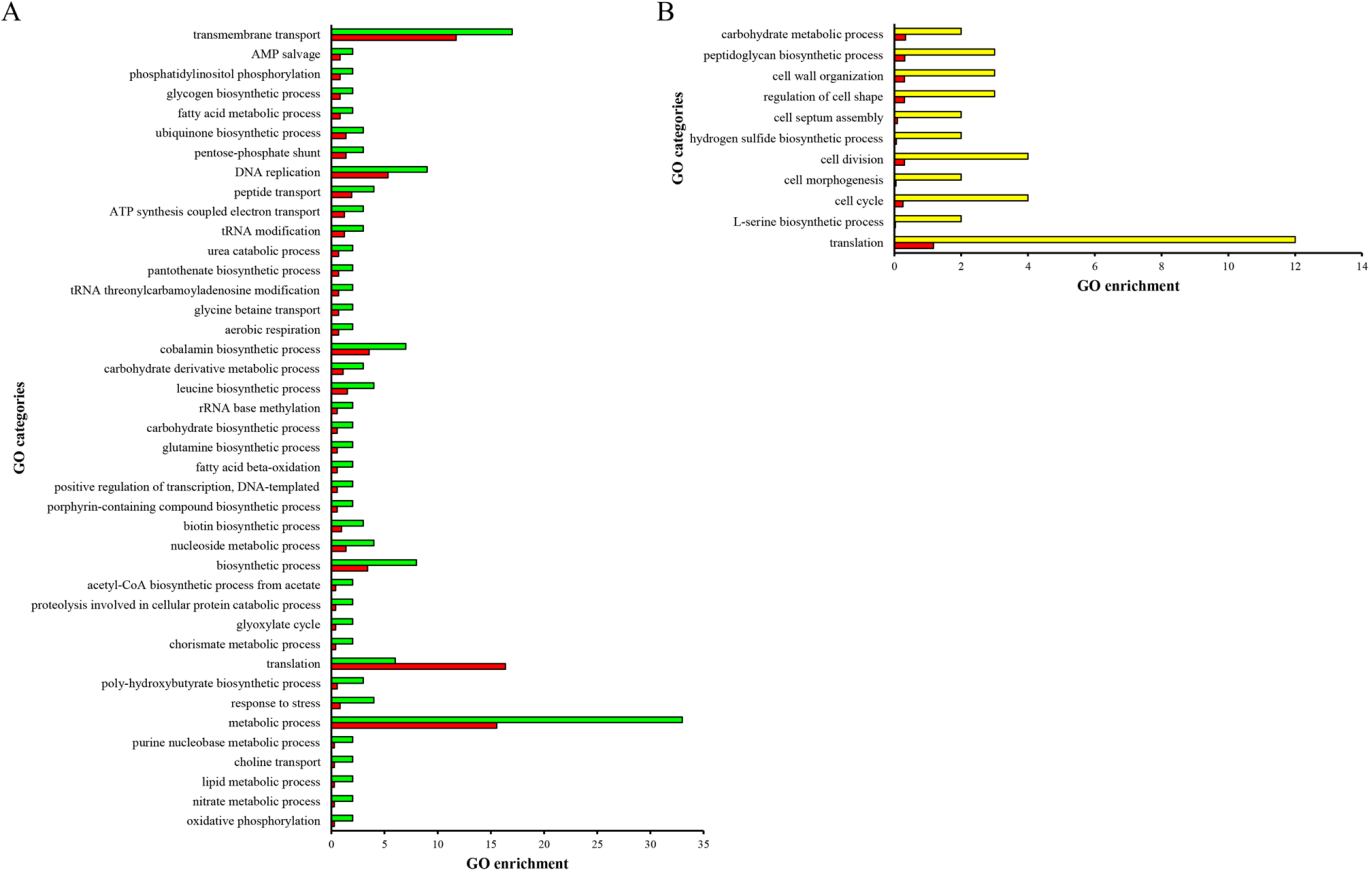
Significant changes in Gene Ontology (GO) groups affected by pH in *P. denitrifcans* PD1222 in the comparative study pH 7.2/pH 6.5. Only changes in GO groups with a *p-*value < 0.05 are shown. The third level of biological function GO was used for proteins with significant differential expression and a hypergeometric test ([E(GOi)]), where [E(GOi)] = sample size/genome size × GOi, was evaluated^21^. In this analysis the *P. denitrificans* PD1222 whole genome was used as reference (red bars) and GO groups of significantly over-represented proteins at pH 7.2 are shown in green bars (**A**), whereas down-represented proteins are shown in yellow bars (**B**).

The Gene Ontology (GO) categories^21^ from the comparative analysis between pH 6.5 and 7.2 are shown (Fig. 2). Breaking down the proteome into GO groups gives insight into functional protein categories that are either enriched (over-represented) or suppressed (down-represented) under denitrifying and nitrate assimilating conditions. Proteins over-represented at pH 7.2 belonged to significantly changed GO groups ‘transmembrane transport’, ‘metabolic process’ ‘pentose phosphate shunt’, ‘pantothenate biosynthetic process’, ‘cobalamin biosynthetic process’, ‘leucine biosynthetic process’, ‘glutamine biosynthetic process’, ‘porphyrin-containing compounds biosynthetic process’, ‘biotin biosynthetic process’, ‘glyoxylate cycle’ and ‘nitrate metabolic process’, among others (Fig. 2A). Down-represented proteins at pH 7.2 belong to the GO groups ‘cell cycle’, ‘cell division’, ‘cell morphogenesis’, ‘regulation of cell shape’, ‘cell wall organization’ and ‘hydrogen sulphide biosynthetic process’ (Fig. 2B).

### Effect of pH on peptide representation of proteins involved in denitrification and nitrate assimilation

**Figure 3:**
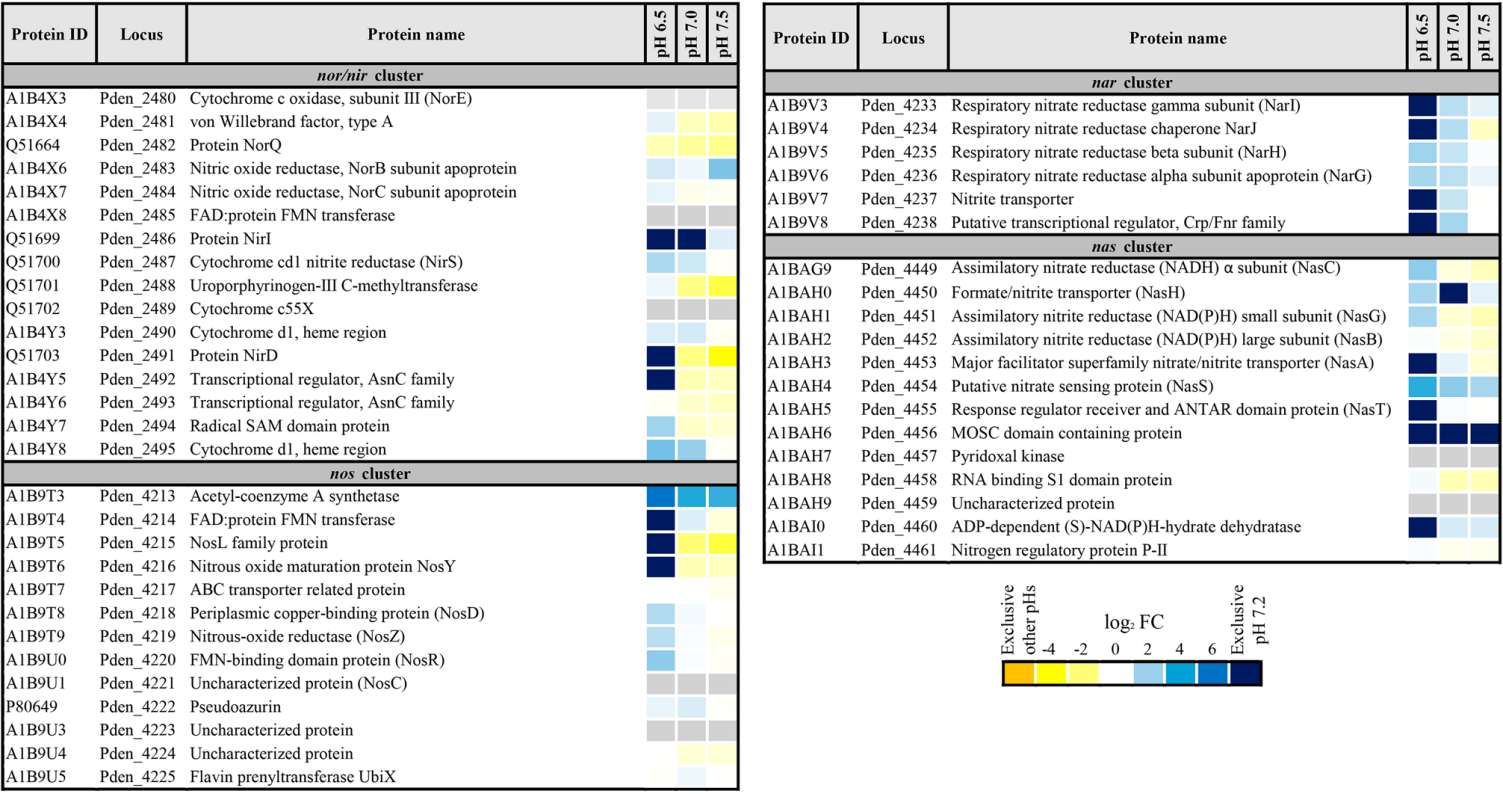
LC–MS/MS analysis of *P. denitrificans* proteins encoded by denitrification and nitrate assimilation gene clusters affected by pH. Heatmap shows the fold changes, represented as log_2_ normalized expression using pH 7.2 as reference. Therefore, positive values correspond to proteins over-represented at pH 7.2 (blue) and negative values correspond to proteins over-represented at pH 6.5, pH 7.0 or pH 7.5 (yellow). Proteins with an absolute fold change > 100 are considered exclusive at pH 7.2 (dark blue) or exclusive at other pHs (orange). Grey color represents proteins not detected in this study. Protein code according to Uniprot database under the accession number UP000000361. This figure was created with the Microsoft Excel software from Microsoft Office version Professional Plus 2019 (https://www.microsoft.com).

The catalytic subunit of the assimilatory nitrate reductase NasC was negatively affected at pH 6.5, showing increased amounts of its peptides at pH 7.2 (3.06-fold change). Increased peptide intensities corresponding to other proteins encoded by the assimilatory *nasTSABGHC* gene cluster of *P. denitrificans*^22^ were also found at pH 7.2, except for the assimilatory nitrite reductase NasB, which was not significantly affected at pH 6.5 (Fig. 3, Table S1).

Several denitrification proteins encoded by the *nar*, *nir*, *nor* and *nos* gene clusters were negatively affected at pH 6.5 (Fig. 3, Table S1). Thus, identified peptides that belong to the catalytic subunit of the respiratory nitrate reductase NarG were over-represented in the proteome at pH 7.2 (3.36-fold change). Additionally, peptides from the *cd*_*1*_-type nitrite reductase NirS and a putative protein involved in its maturation, annotated as cytochrome *d*_*1*_-heme region, were over-represented at pH 7.2 (3.33- and 6.61-fold change, respectively). Furthermore, NosZ, the last denitrification enzyme, was also found over-represented at pH 7.2 (2.80-fold change). However, peptides from nitric oxide reductase NorC were identified and quantified by LC–MS/MS, but they were not significantly affected by extracellular pH. Other proteins participating in the denitrification process were either over-represented at pH 7.2, such as the regulatory protein NosR (4.85-fold change), or exclusive to pH 7.2 like the FMN transferase NosX (Fig. 3, Table S1).

### Determination of enzymic activities involved in nitrate, nitrite and N_2_O reduction

**Figure 4:**
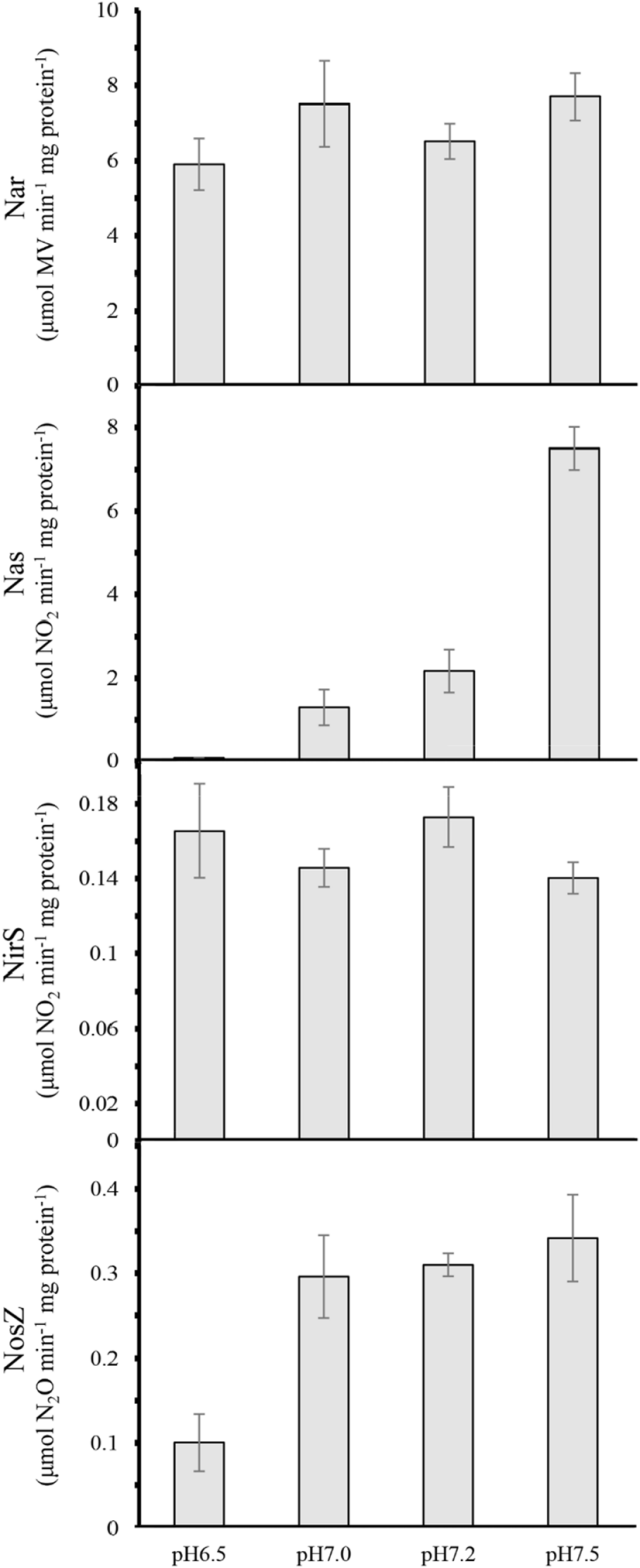
Effect of pH on denitrification enzymes and assimilatory nitrate reductase in *P. denitrificans*. Cells were grown under anaerobic conditions with nitrate (30 mM) as nitrogen and energy source at the indicated pHs. Enzymic activities of respiratory (Nar) and assimilatory (Nas) nitrate reductases, nitrite reductase (NirS) and nitrous oxide reductase (NosZ) were determined as described in “Materials and Methods”. Error bars correspond to data from three independent replicates (n = 3).

Activities of reductases involved in denitrification or nitrate assimilation were determined in *P. denitrificans* cells grown under denitrifying conditions at different pHs (Fig. 4). Nitrite reductase NirS activity was similar at all tested pHs. However, nitrous oxide reductase NosZ activity was markedly decreased at pH 6.5. Both respiratory (Nar) and assimilatory (Nas) nitrate reductases showed the lowest activity at pH 6.5 (Fig. 4).

### Analysis of NosZ copper-centers biogenesis proteins and determination of intracellular copper concentration

The putative copper-processing permease NosY was exclusive to the *P. denitrificans* proteome at pH 7.2 and the periplasmic copper-binding protein NosD was over-represented at pH 7.2 (2.91-fold change) (Fig. 3, Table S1). Interestingly, the intracellular concentration of copper was about 23% higher in cells grown at pH 7.2 than at pH 6.5 (69.10 ± 7.99 and 52.97 ± 5.93 µg Cu/mg protein, respectively).

### Effect of pH on cofactor biosynthetic routes

Intensities of represented peptides from proteins that participate in the biosynthesis of several enzymic cofactors required for denitrification like riboflavin, nicotinamide, molybdopterin, cobalamin, biotin and pyridoxal phosphate were significantly down-represented in the *P. denitrificans* proteome at pH 6.5 compared with the proteome at pH 7.2 (Tables S1 & S4).

Regarding riboflavin biosynthesis, peptides down-represented at pH 6.5 corresponded to diaminohydroxyphosphoribosylaminopyrimidine deaminase, GTP cyclohydrolase-2, riboflavin synthase, HAD superfamily phosphatase, riboflavin synthesis protein and NADPH-dependent FMN reductase (Fig. S3, Tables S1 & S4).

Quantity of peptides belonging to enzymes involved in molybdopterin biosynthesis, such as cyclic pyranopterin monophosphate synthase accessory protein, molybdopterin synthase MoaE, molybdopterin molybdochelatase and molybdenum cofactor guanylyltransferase, was diminished at low pH (Fig. S4, Tables S1 & S4). Additionally, the FAD-binding molybdopterin dehydrogenase was exclusive to pH 7.2.

The synthesis of nicotinamide-derived metabolites could be negatively affected at pH 6.5 because the purine nucleoside phosphorylase, the 5'-nucleotidase SurE, the nicotinate-nucleotide adenylyltransferase, the NAD kinase, the nicotinamidase, the purine nucleosidase, the MazG family protein and the nicotinate-nucleotide pyrophosphorylase were exclusive to the proteome at pH 7.2 (Fig. S5, Tables S1 & S4). Similarly, different enzymes involved in cobalamin biosynthesis were down-represented at pH 6.5. Thus, precorrin-3 methyltransferase, precorrin-4 C11-methyltransferase, precorrin-6A synthase, precorrin-6A reductase, cobaltochelatase CobN, cob(I)yrinic acid a,c-diamide adenosyltransferase and CibX were exclusive to pH 7.2 (Fig. S6, Tables S1 & S4).

Enzymes involved in biotin biosynthesis like 3-hydroxyacyl-(acyl-carrier-protein) dehydratase FabZ, adenosylmethionine-8-amino-7-oxononanoate aminotransferase, ATP-dependent dethiobiotin synthetase BioD, biotin synthase 1 and biotin-acetyl-CoA-carboxylase ligase, were also negatively affected at pH 6.5. Additionally, several biotin-containing enzymes were found diminished at pH 6.5, such as biotin carboxyl carrier protein, 3-methylcrotonyl-CoA carboxylase and propionyl-CoA carboxylase carboxyltransferase subunit (Tables S1 & S4).

Finally, key enzymes in the biosynthesis of pyridoxal 5’-phosphate were negatively affected at pH 6.5, including the 4-hydroxythreonine-4-phosphate dehydrogenase and the pyridoxine/pyridoxamine 5'-phosphate oxidase/pyridoxal 5'-phosphate synthase, which were exclusive to pH 7.2 (Tables S1 & S4). Several pyridoxal phosphate-dependent aminotransferases were also found decreased at pH 6.5, including the branched-chain amino acid aminotransferase (3.48-fold change), and the adenosylmethionine-8-amino-7-oxononanoate aminotransferase, which was exclusive to pH 7.2 (Tables S1 & S4).

### Central metabolic pathways affected by pH

In the comparative proteomic analysis between proteomes at pH 6.5 and pH 7.2, different enzymes involved in central metabolic routes were also found down-represented at pH 6.5. Thus, the tricarboxylic acid (TCA) cycle and glyoxylate shunt enzymes fumarate hydratase and isocitrate lyase were exclusive to pH 7.2, and malate dehydrogenase and malate synthase were over-represented at pH 7.2 (2.52- and 3.49-fold change, respectively) (Table S1). Glucose-6-phosphate dehydrogenase and 6-phosphogluconolactonase, involved in the pentose phosphate route, were also exclusive to pH 7.2. Additionally, two vitamin B_12_-dependent enzymes with ribonucleoside-diphosphate reductase-class II activity were over-represented at pH 7.2 (4.05- and 2.56-fold change, respectively) (Table S1).

Coenzyme A biosynthetic pathway was also negatively affected at low pH, especially the enzymes pantothenate synthetase and phosphopantetheine adenylyltransferase, which were exclusive to pH 7.2 (Tables S1 & S4). Additionally, acetyl-coenzyme A synthetase and the coenzyme A-dependent enzymes acetyl-CoA acetyltransferases, 3-methylcrotonyl-CoA carboxylase and propionyl-CoA carboxylase (carboxyltransferase subunit), and two methylmalonyl-CoA mutases were also over-represented at pH 7.2 (Table S1).

### Effect of pH on the expression of genes encoding cofactor biosynthesis proteins

To explore if the extracellular pH 6.5 affects negatively not only peptide quantities but also gene transcript levels, a qPCR analysis was carried out. For this purpose, expression of different genes encoding key enzymes involved in major coenzyme biosynthetic pathways was evaluated in cells grown at pH 6.5 or 7.2. Results showed that expression of selected genes was significantly decreased at pH 6.5 (Table 2).

**Table 2:** Gene expresión analysis by qPCR of several genes involved in biosynthesis of cofactors in *P. denitrificans* PD1222.

Gene ID^1^	Protein ID^2^	Name	Relative expression pH 6.5	Relative expression pH 7.2	Fold change^3^
Pden_3662	A1B885	Riboflavin biosynthesis protein (EC 2.7.1.26)	0.03 ± 0.01	0.19 ± 0.05	5.68
Pden_2141	A1B3Y9	Molybdopterin molybdochelatase	0.09 ± 0.01	0.19 ± 0.03	2.06
Pden_0851	A1B0B9	Nicotinamidase (EC 3.5.1.19)	1.23 ± 0.01	2.90 ± 0.03	2.35
Pden_2529	A1B522	Cob(I)yrinic acid a,c-diamide adenosyltransferase (EC 2.5.1.17)	0.07 ± 0.01	0.20 ± 0.01	3.03
Pden_1658	A1B2L1	Pantothenate synthetase (PS) (EC 6.3.2.1)	0.20 ± 0.03	0.69 ± 0.01	3.43
Pden_1433	A1B1Z0	Biotin synthase 1 (EC 2.8.1.6)	1.34 ± 1.61	10.16 ± 2.12	7.56
Pden_2918	A1B658	ATP-dependent dethiobiotin synthetase BioD (EC 6.3.3.3)	3.82 ± 1.21	8.04 ± 2.81	2.10
Pden_2731	A1B5M1	5'-nucleotidase SurE (EC 3.1.3.5)	0.47 ± 0.04	1.86 ± 0.05	3.99

^1^Protein annotated from UniProt (UP000000361). ^2^Genes annotated from GeneBank (T00440). ^3^Fold change calculated as the ratio gene expression at pH 7.2 and pH 6.5 (using pH 7.2 as reference condition). The primers used are described in Table S5. Data are means ± SD of three independent biological replicates (n = 3).

## Discussion

### Influence of pH on the biogenesis of the copper-containing NosZ and other denitrification proteins in *P. denitrificans*

*P. denitrificans* PD1222 is a soil denitrifying bacterium able to grow with nitrate as terminal electron acceptor under anaerobic conditions^3,23^. Denitrification is widespread in nature among ecosystems that display different physicochemical properties like pH. Soil pH is a key parameter affecting denitrification, as previously described in *P. denitrificans*, which shows high nitrous oxide emissions and reduced copper-dependent nitrous oxide reductase activity (NosZ) at pH 6.5^24,25^. In this work, the effect of pH on denitrification and general metabolic processes was determined in *P. denitrificans* PD1222 at proteomic level. This strain was cultured under denitrifying and nitrate-assimilating conditions at different pHs from 6.5 to 7.5. Independently of the tested pH, *P. denitrificans* was able to growth supporting substantial nitrate denitrification and converting almost all nitrate into dinitrogen (Fig. 1A, Table 1). Results suggest that at pH 7.2 the overall electron flux through the first three reactions of denitrification matches or exceeds that of the final step catalysed by NosZ. However, at pH 6.5 electron flux through the nitrous oxide reductase becomes limiting, leading to high N_2_O emissions (Table 1).

A high number of proteins were identified in the proteomic analysis of *P. denitrificans* at different pHs (Fig. 1B). The most drastic changes were found when compared the proteomes at pH 6.5 and 7.2 (Table S1), affecting proteins belonging to significative GO groups that were over-represented at pH 7.2 (down-represented at pH 6.5) (Fig. 2A). However, changes in the *P. denitrificans* proteome at pH 6.5 do not correlate with variations of the intracellular pH, which was kept at 6.5 ± 0.1, independently of the initial extracellular pH. This result demonstrates, for the first time, pH homeostasis in *P. denitrificans* during denitrification. Maintenance of intracellular pH when conditions are fluctuating is crucial for cell survival.

Periplasmic nitrous oxide reductase NosZ, the last denitrification enzyme, contains two types of copper centers, a binuclear center Cu_A_ that functions as electron acceptor and a tetranuclear center Cu_Z_ at the active site^26,27^. In *P. denitrificans*, NosZ activity is sensitive to extracellular changes in pH, but *nosZ* gene expression is not altered, suggesting that pH affects protein synthesis and/or assembly^24,25^. Furthermore, the bacterium *Marinobacter hydrocarbonoclasticus* is unable to reduce N_2_O at pH 6.5, displaying low amounts of copper-loaded NosZ enzyme at this pH^28^. Reduced NosZ activity (Fig. 4) could be caused by the low representation of NosZ in the *P. denitrificans* proteome at pH 6.5 (Fig. 3, Table S1). Additionally, two proteins putatively involved in copper transport, NosD and NosY, were over-represented or exclusive at pH 7.2, respectively (Fig. 3, Table S1). Inactivation of any of the *nosDFY* genes, which cluster together the *nosZ* gene in *P. dentrificans* and other denitrifiers, results in a NosZ enzyme that lacks the Cu_Z_ center, suggesting that these genes code for a three-component complex involved in copper transport or assembly to the apoprotein^26,27^. Furthermore, the predicted function of NosF as an ATPase and the general distribution of the complex to both sides of the cytoplasmic membrane also suggest that the NosDFY complex acts as a copper transporter. However, a role of NosDFY in the biogenesis of the copper centers of NosZ could not be discarded. In this sense, an involvement of this complex in sulphide transport associated with the assembly of the copper sulphide Cu_Z_ centre of NosZ has been suggested in the light of the sequence similarity shared between NosDFY and Atm1, which is involved in assembly of mitochondrial iron-sulphur clusters^29,30^. Independently of the involvement of the NosDFY complex in copper transport and/or NosZ Cu_Z_ center assembly, our work highlights a decrease of both the peptide intensities of NosDY and the intracellular copper concentration at pH 6.5 in *P. dentrificans*. Therefore, pH 6.5 affects negatively the NosZ polypeptide amount, and probably the assembly of its copper centers, explaining the low NosZ activity.

Although denitrifying bacteria are phylogenetically diverse, *P. denitrificans* PD1222 can be considered a typical denitrifier. Our results demonstrate that slightly acidic pH provokes drastic changes in the proteome of this bacterium. Thus, the denitrification reductases NarG, NirS and NosZ were negatively affected at pH 6.5, and the influence of pH was even more evident in the case of the nitrous oxide reductase NosZ, probably because its maturation could not be fully completed under copper-limiting conditions at this pH. This fact provides a direct link between an immature NosZ at low pH, and in turn, affecting soil N_2_O emissions. The regulatory flavoprotein NosR that may control *nos* gene expression^31^ was over-represented at pH 7.2. The *nos* gene cluster of *P. denitrificans* also includes a gene encoding the FAD-containing FMN transferase NosX, which was exclusive to pH 7.2 (Fig. 3, Table S1). The role of NosX remains unclear, but its homologue NirX that is encoded by a gene of the *P. denitrificans nir* cluster was unaffected by pH changes (Fig. 3). It has been reported that NosZ activity is eliminated in a double *nosX/nirX* mutant, although single mutations of these genes do not result in any apparent phenotype^32^. NosR and NosX/NirX proteins could provide a link between N_2_O consumption and flavin biosynthesis. In this sense, several enzymes that participate in the biosynthesis of riboflavin in *P. denitrificans* were significantly diminished at pH 6.5 (Fig. S3, Tables S1 & S4), and as result, a great variety of FAD- or FMN-containing enzymes were found down-represented at this pH. Regarding nitrous oxide reduction, these enzymes include the FAD-containing FMN transferase NosX and the FMN-binding domain protein NosR (Table S1). These two proteins are involved in regulation of *nosZ* gene expression and activation of NosZ protein, and therefore their reduced levels at pH 6.5 could also impact negatively on the formation of an active NosZ enzyme.

### Effect of pH on nitrate assimilation, cofactor biosynthetic routes and central metabolic pathways

All key steps in denitrification and nitrate assimilation are dependent on a range of cofactors. Therefore, it is not possible to consider these processes without additional exploration in the proteome of cofactor biosynthesis enzymes. Many different proteins involved in the synthesis of molybdopterin, nicotinamide adenine dinucleotide, and cobalamin, among others enzymic cofactors, were exclusive to pH 7.2 or down-represented at pH 6.5 (Tables S1 & S4). Almost all these proteins displayed a cytoplasmic location according to PSOTb v3.0.2^33^. In *P. denitrificans*, the assimilatory nitrate reduction system includes an NADH-dependent nitrate/nitrite reductase complex (NasCBG) that reduces nitrate to ammonium^22,34,35^. This proteomic study has revealed that the molybdopterin-containing subunit NasC, and other components of the Nas system, were negatively affected at pH 6.5 (Fig. 3, Table S1). The FAD-binding molybdopterin dehydrogenase and other enzymes involved in molybdopterin biosynthesis were exclusive or over-represented at pH 7.2 (Fig. S5, Tables S1 & S4). This fact could contribute to the decreased peptide levels and reductase activity of NasC at pH 6.5, and this may be extended to the respiratory nitrate reductase NarG, which also binds a molybdopterin guanine dinucleotide cofactor (Fig. 4, Table S1). Synthesis of nicotinamide adenine dinucleotide, another relevant cofactor for nitrate assimilation, is negatively affected at pH 6.5, considering that peptides from several enzymes involved in the formation of its precursors (nicotinamide, nicotinate and quinolinate) were exclusive at pH 7.2 (Fig. S5, Tables S1 & S4). Consistent with these results, several NAD(P)H-dependent enzymes were diminished at low pH in *P. denitrificans*, including the assimilatory nitrate reductase NasC (Table S1). Perhaps for its NADH-dependence, Nas activity is even more drastically affected at pH 6.5 than Nar activity (Fig. 4). Denitrification also depends on heme, siroheme and cobalamin (vitamin B_12_) as cofactors. Biosynthetic routes of these cofactors share uroporphyrinogen III as common intermediate. In *P. denitrificans*, the cobalamin biosynthetic pathway could be severely affected at pH 6.5 because many enzymes involved in this route were exclusive at pH 7.2 (Fig. S6, Tables S1 & S4). Copper deficiency increases N_2_O emissions by *P. denitrificans*, and this condition leads to switching from vitamin B_12_-dependent to vitamin B_12_-independent pathways through transcriptional modulation of genes controlled by vitamin B_12_ riboswitches^36^. Although N_2_O emissions increased at pH 6.5 (Table 1), no differences were observed in the comparative proteomic analysis concerning to vitamin B_12_-dependent or independent metabolic pathways. Cobalamin is a key cofactor for a complete denitrification, but also it plays an important role in remodelling terrestrial habitats as demonstrated by a recent metagenomic study^37^. Integration of denitrification with central metabolic pathways is required to generate necessary reducing power. Furthermore, in a previous study it was found that levels of acetyl-CoA increased during denitrification, suggesting a tight link between carbon and nitrogen metabolism during this process^20^. Several enzymes of the TCA and glyoxylate cycles were down-represented at pH 6.5. Additionally, the acetyl-coenzyme A synthetase encoded by a gene that clusters together the *nos* genes of *P. denitrificans* was negatively affected at pH 6.5. Many enzymes involved in fatty acid metabolism are coenzyme A-dependent and use NAD^+^ or FAD, being altered by the negative effect of low pH on the biosynthesis of these cofactors (Tables S1 & S4). In this regard, down-regulation of the NAD(P)H biosynthetic route could also explain why the pentose phosphate pathway was sensitive to low pH. This fact, in turn, may negatively affect nucleoside and nucleotide metabolism and enzymes using phosphoribosyl pyrophosphate as substrate (Table S1). Biotin is a cofactor present in many carboxylases that are involved in carbohydrates, fatty acids and branched-chain amino acids metabolism^38^. Biotin biosynthesis could be also negatively affected at pH 6.5 (Table S4), and the inability to satisfy cellular demand for biotin may have negative consequences on cell viability. On the other hand, key enzymes required for biosynthesis of pyridoxal 5’-phosphate were down-represented at low pH in *P. denitrificans* **(**Tables S1 & S4), leading to reduced levels of several aminotransferases like the branched-chain amino acid aminotransferase involved in pantothenate biosynthesis (Table S4). Extracellular low pH not only affected the biosynthesis of relevant cofactors required for denitrification and other metabolic processes at proteomic level, but also had a negative impact on expression of essential genes encoding cofactor biosynthetic enzymes (Table 2). The negative effect of pH 6.5 on gene expression and protein synthesis and folding is a relevant finding that should be consider for soil microorganisms that carry out many different metabolic processes putatively affected by acidic environments.

In conclusion, denitrification, nitrate assimilation and cofactor biosynthetic routes were negatively affected at pH 6.5 in *P. denitrificans.* Although Nar activity diminished at pH 6.5, the overall rate of nitrate consumption through to nitrous oxide remained similar and protein synthesis was not affected sufficiently to limit electron flux. This is not the case for the copper-containing NosZ, which was drastically decreased at pH 6.5, leading to a large increase in emissions of the potent greenhouse gas nitrous oxide. This may be also related with the reduced levels of NosD and NosY proteins involved in copper transport and/or copper-sulphide Cu_Z_ center biogenesis of NosZ, and with the low intracellular copper concentration at pH 6.5. Despite all these effect of low pH on the proteome, *P. denitrificans* cells are able to maintain the intracellular pH, thus highlighting the importance of pH homeostasis during denitrification.

## Materials and methods

### Bacterial strain, media and growth conditions

*P. denitrificans* PD1222 was grown under anaerobic conditions at 30 °C in a defined mineral salt medium^39^ with potassium nitrate (30 mM) as nitrogen source and electron acceptor, and sodium succinate (30 mM) as sole carbon source. Initial pH of the media was adjusted to 6.5, 7.0, 7.2 or 7.5. In all cases, an aerobic overnight culture with ammonium chloride (10 mM), was used as inoculum at an initial absorbance at 600 nm (A_600_) of about 0.01. Cells were harvested either at A_600_ ~ 0.3 (early exponential growth phase) or at A_600_ ~ 0.9 (late exponential growth phase), as indicated in each experiment. Spectinomycin (25 µg/ml) was used as antibiotic.

### Enzymic activity assays

Respiratory nitrate reductase (Nar), assimilatory nitrate reductase (Nas), nitrite reductase (NirS) and nitrous oxide reductase (NosZ) activities were assayed as previously described^20,40^. To obtain subcellular fractions, cells were incubated with lysozyme and centrifugated (10,000 rpm, 4 °C, 15 min) and the supernatants, corresponding to the periplasmic fractions, were used to determine NirS activity, as previously described^40^. Pellets containing the spheroplasts were resuspended in 1 ml of Tris–HCl buffer (100 mM, pH 8.0) supplemented with a few granules of DNase and broken by sonication (3 pulses of 10 s, 90 W). Unbroken spheroplasts were removed by centrifugation and membrane and cytoplasmic fractions were collected by ultracentrifugation (45,000 rpm, 4 °C, 45 min). Cytoplasmic fractions (supernatants) were used to determine Nas activity and membrane fractions (pellets) were homogenized in 1 ml of Tris–HCl buffer (50 mM, pH 8.0) to measure Nar activity as previously reported^20,40^. Nitrous oxide reductase activity was measured in vivo by using whole cells placed in sealed 10-ml serum vials under helium atmosphere as previously described^20,41^. Enzymic activities were assayed from three separate independent cultures at the indicated pHs. Protein concentration was estimated in subcellular fractions by the method of Bradford^42^ or in whole cells following a modification of the Lowry procedure^43^.

### In vivo gas emissions and extracellular nitrate and nitrite determinations

*P. denitrificans* PD1222 was cultured with nitrate (30 mM) in 30 ml-sealed tubes with 15 ml of Ar-anaerobic atmosphere at initial pH 6.5 or 7.2. After inoculation, tubes were placed onto ice and sparged with argon for 1 h until molecular nitrogen was not detected by GC. Cells were incubated at 30 °C for bacterial growth. When cells reached an A_600_ of approximately 0.3 or 0.9, 1 ml from the headspace was analysed by gas chromatography to determine N_2_O and N_2_ as previously described^41^. Nitrate in the media was determined by using a method based on the incubation with sulfamic and perchloric acids^44^, and nitrite was measured colorimetrically at 540 nm as previously described^45^.

### Determination of intracellular copper concentration by ICP-MS

To determine the intracellular content of copper, 100 ml of *P. denitrificans* cells grown at the pH 6.5 or 7.2 were harvested upon reaching an A_600_ of 0.3. Cells were washed in 20 ml of a buffer solution with Tris–HCl (20 mM, pH 8.0) and EDTA (4 mM). After centrifugation, pellets were dried (80 °C, 96 h), weighted and subjected to digestion with high-purity nitric acid. Copper measurements were carried out by Inductively Coupled Plasma Mass Spectrometry (ICP-MS, PerkinElmer Nexion350X) at the Central Service for Research Support (SCAI), University of Córdoba. Six different biological samples were analysed for each condition.

### Determination of intracellular pH

The intracellular pH of *P. denitrificans* PD1222 cells was determined as previously reported^46,47^. Cells were grown (per triplicate) in minimal media initially adjusted at pH values of 6.5, 7.2, 7.0 or 7.5. When cultures reached an A_600_ of approximately 0.3, 2 ml aliquots were harvested for each pH, washed and resuspended in 200 μl of minimal media (adjusted to the pH fixed for growth). Collected samples were divided in two aliquots, centrifuged and resuspended in 200 μl of the same media, in the presence or absence of 2′,7′-bis(2-carboxyethyl)*-*5(6)-carboxyfluorescein tetrakis(acetoxymethyl) ester (50 μM) (Millipore, Massachusetts, USA). In situ calibration was performed replacing the minimal media by MES buffer (50 mM) for pHs 6.0, 6.5 and 7.0 or Bis–Tris buffer (50 mM) for pH 7.5. Background was determined considering samples without the fluorophore. All samples were incubated at 30 °C for 1 h with gentle shanking in darkness. Cells were centrifuged (12,000 rpm, 15 min) and washed three times in 1 ml of the corresponding buffer without the fluorophore. Finally, cells were resuspended in 200 μl of 1/10 diluted minimal media adjusted at different pHs or in the buffer used for in situ calibration, as appropriate. Fluorescent measurements were performed using a Flex Station 3 (Molecular Devices- LLC, California, USA) at room temperature. Samples were sequentially excited at two wavelengths, 450 nm and 490 nm, and emission fluorescence was detected at 535 nm for each of the two excitation wavelengths. Measurements were repeated three times for each sample.

### Proteomic analysis

*P. denitrificans* cells were grown anaerobically under denitrification conditions at pH 6.5, 7.0, 7.2 or 7.5. When cultures reached an A_600_ of about 0.3, cells were harvested by centrifugation (12,000 rpm, 15 min) and kept at − 80 °C until use. Samples for proteomic analysis by LC–MS/MS were prepared by resuspension of frozen cells in 300 µl of a lysis buffer containing Tris–HCl (50 mM, pH 8.0), CHAPS (4%) and urea (8 M). Proteins extracted from cells broken by cavitation (3 pulses of 10 s, 90 W) were cleaned with the 2-D Clean-UP Kit (GE Healthcare, Little Chalfont, UK). Proteins were quantified and analysed by LC–MS/MS at SCAI, University of Cordoba, as previously described^20^. Data were used to generate a heat-map and a volcano plot (Figs. S1 & S2). Relative protein quantification after LC–MS/MS was performed by the MaxQuant software^48^ on the three most intense peptides. Results were then filtered considering a *p-*value ≤ 0.05 and a fold change ≥ 2. Data were deposited to the ProteomeXchange Consortium (http://proteomecentral.proteomexchange.org) via the PRIDE partner repository^49^ (dataset identifier PXD013138). Bioinformatic platform BLAST2GO^50^ was used to predict the protein subcellular location. GO analysis were performed using the web application ComparativeGO^21^. As a source of information and to perform integration of final proteomic data the tool KEGG Mapper was used. Only label-free quantification (LFQ) intensity values of differentially expressed proteins with respect to the reference condition (pH 7.2) were used. Data were used for PCA analysis and for generation of a hierarchical cluster by using Euclidean distance.

### Transcriptomic analysis by qPCR

*P. denitrificans* cells were washed three times in 1 ml of TEG buffer containing Tris–HCl (25 mM, pH 8.0), glucose (1%) and EDTA (10 mM). RNA isolations were performed using the Qiagen RNA extraction kit (RNeasy midi kit). DNase incubation was carried out in the column with RNase-free DNase set (Qiagen) and an additional post-column treatment was required with DNase I (Ambion). The concentration and purity of the RNA samples were measured by using a ND1000 spectrophotometer (Nanodrop Technologies). Data were normalized using *dnaN* as housekeeping gene that codes for the DNA polymerase III β-subunit. Synthesis of total cDNA and PCR reactions were carried out with specific primers (Table S5) as previously described^20^.

### Statistical analysis

Statistical significance was analysed by a two-tailed t-test analysis, considering that pair of samples were different when the *p* value was lower than 0.05. Perseus (v1.6.5.0) software was used for the proteomic data analysis. Other data were compared using the IBM SPSS Statistics v22 software.

## Supplementary Information

Supplementary Information 1.

Supplementary Information 2.
