# Supplementary material for: Effect of pH on the denitrification proteome of the soil bacterium Paracoccus denitrificans PD1222
Source: Sci Rep. 2021 Aug 26;11:17276. doi: 10.1038/s41598-021-96559-2 (PMC8390676; doi:10.1038/s41598-021-96559-2)
Supplement: Supplementary file 1 — Supplementary Information 1. [file 41598_2021_96559_MOESM1_ESM.pdf]

**Supplementary material (figures, figure legends and cited reference) to "Effect of pH on the denitrification proteome of the soil bacterium *Paracoccus denitrificans* PD1222"**

Alfonso Olaya-Abril

*Departamento de Bioquímica y Biología Molecular, Universidad de Córdoba, Edificio Severo Ochoa, 1ª planta, Campus de Rabanales, Córdoba, 14071, Spain*

Jesús Hidalgo-Carrillo

D*epartamento de Química Orgánica, Universidad de Córdoba, Edificio Marie Curie, Campus de Rabanales, Córdoba, 14071, Spain*

Víctor M. Luque-Almagro

*Departamento de Bioquímica y Biología Molecular, Universidad de Córdoba, Edificio Severo Ochoa, 1ª planta, Campus de Rabanales, Córdoba, 14071, Spain*

Carlos Fuentes-Almagro

*Servicio Central de Apoyo a la Investigación (SCAI), Unidad de Proteómica, Universidad de Córdoba, Campus de Rabanales, Córdoba, 14071, Spain*

Francisco J. Urbano

*Departamento de Química Orgánica, Universidad de Córdoba, Edificio Marie Curie, Campus de Rabanales, Córdoba, 14071, Spain*

Conrado Moreno-Vivián

*Departamento de Bioquímica y Biología Molecular, Universidad de Córdoba, Edificio Severo Ochoa, 1ª planta, Campus de Rabanales, Córdoba, 14071, Spain.*

David J. Richardson

*School of Biological Sciences, University of East Anglia, Norwich, NR4 7TJ, UK*

María Dolores Roldán

*Departamento de Bioquímica y Biología Molecular, Universidad de Córdoba, Edificio Severo Ochoa, 1ª planta, Campus de Rabanales, Córdoba, 14071, Spain*

This SM provides supplementary information on "proteomic and qPCR data analyses" of the Effect of pH on the Denitrification Proteome of the Soil Bacterium *Paracoccus denitrificans* PD1222.

## FIGURE LEGENDS

**Figure S1.** PCA analysis of *P. denitrifcans* PD1222 proteomes at different pHs. The analysis was carried out in Perseus (version 1.6.2.3). Only LFQ intensity values of differentially expressed proteins with respect to the reference condition (pH 7.2) were used. Missing values were imputed with the value of the mean of the five lowest LFQ normalized intensity values of the whole experiment. Resulting data were normalized by Z-score using the mean without grouping.

**Figure S2.** Hierarchical clustering of *P. denitrifcans* PD1222 proteome obtained by LC-MS/MS analyses at different pHs. Clustering approach was carried out in Perseus (version 1.6.2.3). Values previously assigned were used for a hierarchical cluster by using Euclidean distance. Eleven clusters representing different expression profiles were defined by using a distance threshold of 3.43253755569458.

**Figure S3.** Effect of pH on riboflavin biosynthetic pathways in *P. denitrificans*. The biosynthetic routes are shown with heatmaps of over-represented (blue) and exclusive (dark blue) proteins at the reference pH 7.2 or down-represented (yelow) and exclusive (orange) proteins at the tested pH (6.5, 7.0 or 7.5). Fold change is represented as $Log_2$. Protein code according to Uniprot database under the accession number UP000000361. Pathway data were obtained from KEGG (https://www.kegg.jp/) under the accession code pde00740 [1].The software used to create the supplementary figures S3, S4, and S6 was Microsoft PowerPoint de Microsoft Office Professional Plus 2019 (https://www.microsoft.com).

**Figure S4.** Effect of pH on the molybdopterin biosynthetic pathway in *P. denitrificans*. The biosynthetic route is shown with heatmaps of over-represented (blue) and exclusive (dark blue) proteins at the reference pH 7.2 or down-represented (yelow) and exclusive (orange) proteins at the tested pH (6.5, 7.0 or 7.5). Fold change is represented as $Log_2$. Protein code according to Uniprot database under the accession number UP000000361. Pathway data were obtained from KEGG (https://www.kegg.jp/) under the accession code pde00790 [1]. The software used to create the supplementary figures S3, S4, and S6 was Microsoft PowerPoint de Microsoft Office Professional Plus 2019 (https://www.microsoft.com).

**Figure S5.** Effect of pH on the biosynthetic pathways of nicotinamide and nicotinate in *P. denitrificans*. The biosynthetic routes of nicotinamide and nicotinate are shown with

heatmaps of over-represented (blue) and exclusive (dark blue) proteins at the reference pH 7.2 or down-represented (yelow) and exclusive (orange) proteins at the tested pH (6.5, 7.0 or 7.5). Fold change is represented as $Log_2$. Protein code according to Uniprot database under the accession number UP000000361. Pathway data were obtained from KEGG (https://www.kegg.jp/) under the accession code pde00760 [1].The software used to create the supplementary figures S3, S4, and S6 was Microsoft PowerPoint de Microsoft Office Professional Plus 2019 (https://www.microsoft.com).

**Figure S6.** Effect of pH on the biosynthesis of vitamin $B_{12}$ in *P. denitrificans*. The biosynthetic route is shown with heatmaps of over-represented (blue) and exclusive (dark blue) proteins at the reference pH 7.2 or down-represented (yelow) and exclusive (orange) proteins at the tested pH (6.5, 7.0 or 7.5). Fold change is represented as $Log_2$. Protein code according to Uniprot database under the accession number UP000000361. Pathway data were obtained from KEGG (https://www.kegg.jp/) under the accession code pde00860 [1]. The software used to create the supplementary figures S3, S4, and S6 was Microsoft PowerPoint de Microsoft Office Professional Plus 2019 (https://www.microsoft.com).

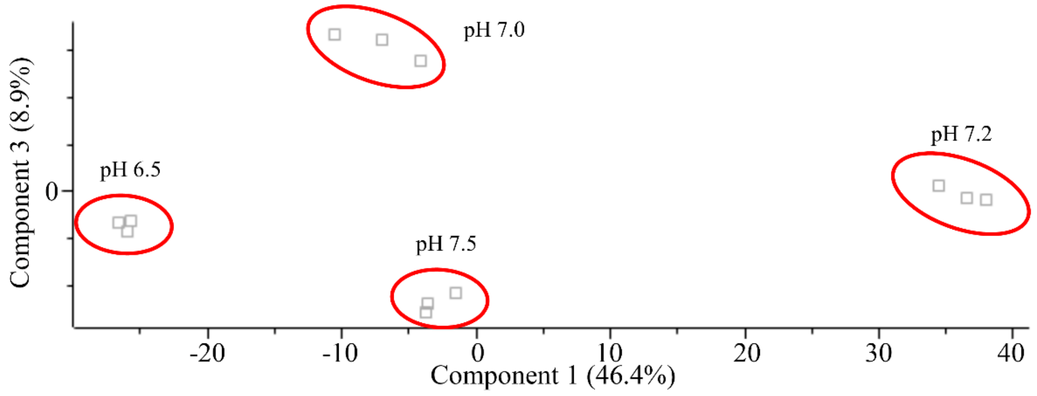
pH 7.0
pH 7.2
pH 6.5
pH 7.5
Component 3 (8.9%)
0
-20
-10
0
10
20
30
40
Component 1 (46.4%)

Figure S1

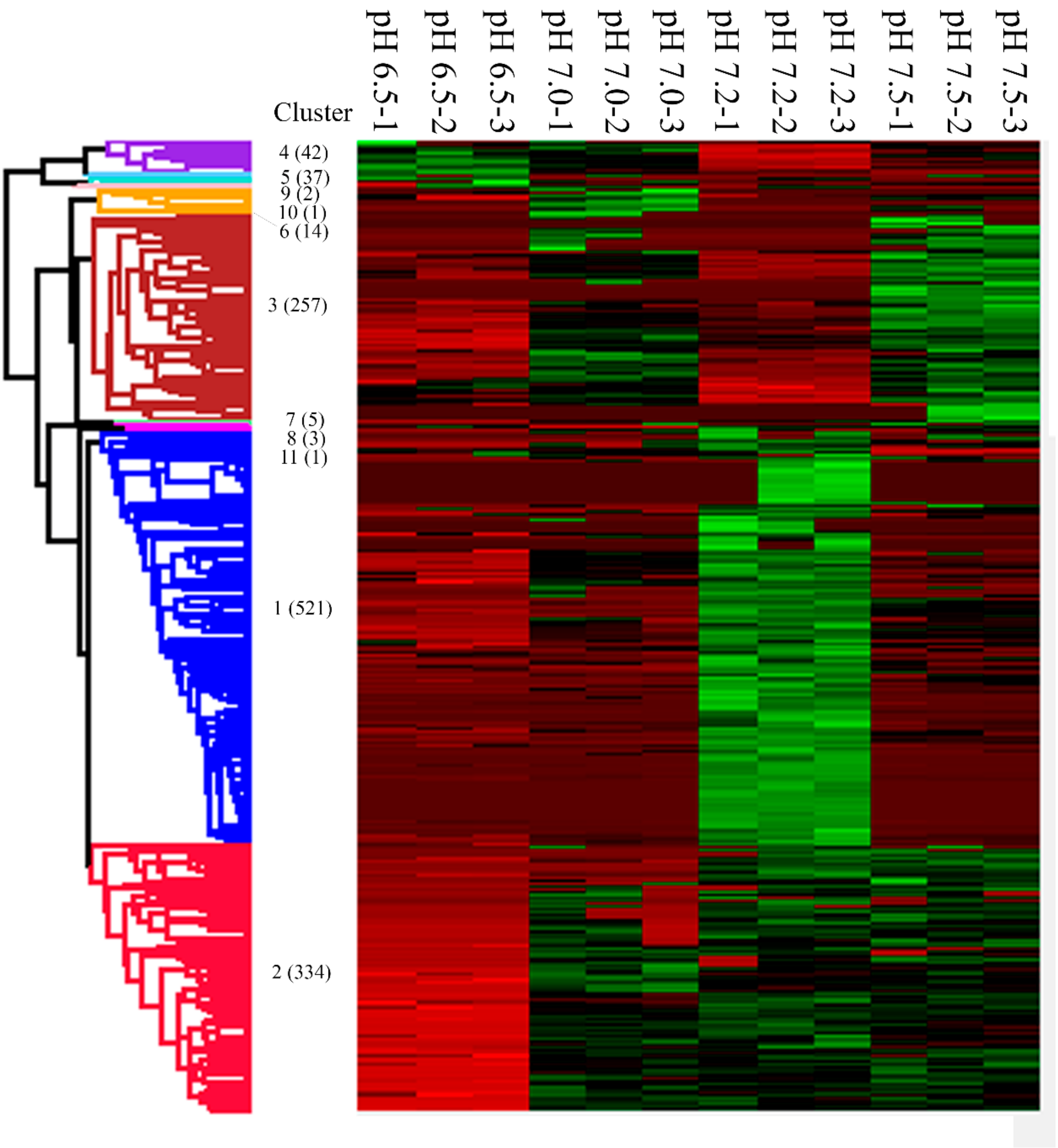
pH 6.5-1
pH 6.5-2
pH 6.5-3
pH 7.0-1
pH 7.0-2
pH 7.0-3
pH 7.2-1
pH 7.2-2
pH 7.2-3
pH 7.5-1
pH 7.5-2
pH 7.5-3
Cluster
4 (42)
5 (37)
9 (2)
10 (1)
6 (14)
3 (257)
7 (5)
8 (3)
11 (1)
1 (521)
2 (334)

Figure S2

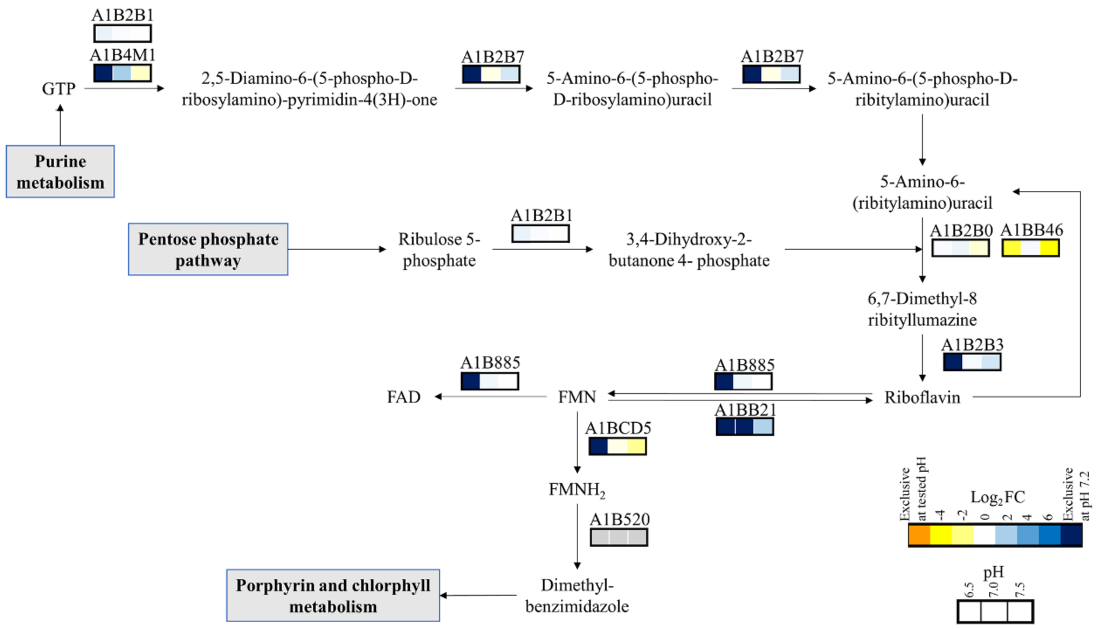
A1B2B1
A1B4M1
GTP
Purine metabolism
2,5-Diamino-6-(5-phospho-D-ribosylamino)-pyrimidin-4(3H)-one
A1B2B7
5-Amino-6-(5-phospho-D-ribosylamino)uracil
A1B2B7
5-Amino-6-(5-phospho-D-ribitylamino)uracil
5-Amino-6-(ribitylamino)uracil
Pentose phosphate pathway
Ribulose 5-phosphate
A1B2B1
3,4-Dihydroxy-2-butanone 4- phosphate
A1B2B0
A1BB46
6,7-Dimethyl-8 ribityllumazine
A1B2B3
A1B885
FAD
FMN
A1B885
A1BB21
Riboflavin
A1BCD5
FMNH2
A1B520
Dimethyl-benzimidazole
Porphyrin and chlorphyll metabolism
Exclusive at tested pH
Log2FC
-4
-2
0
2
4
6
Exclusive at pH 7.2
pH
6.5
7.0
7.5

Figure S3

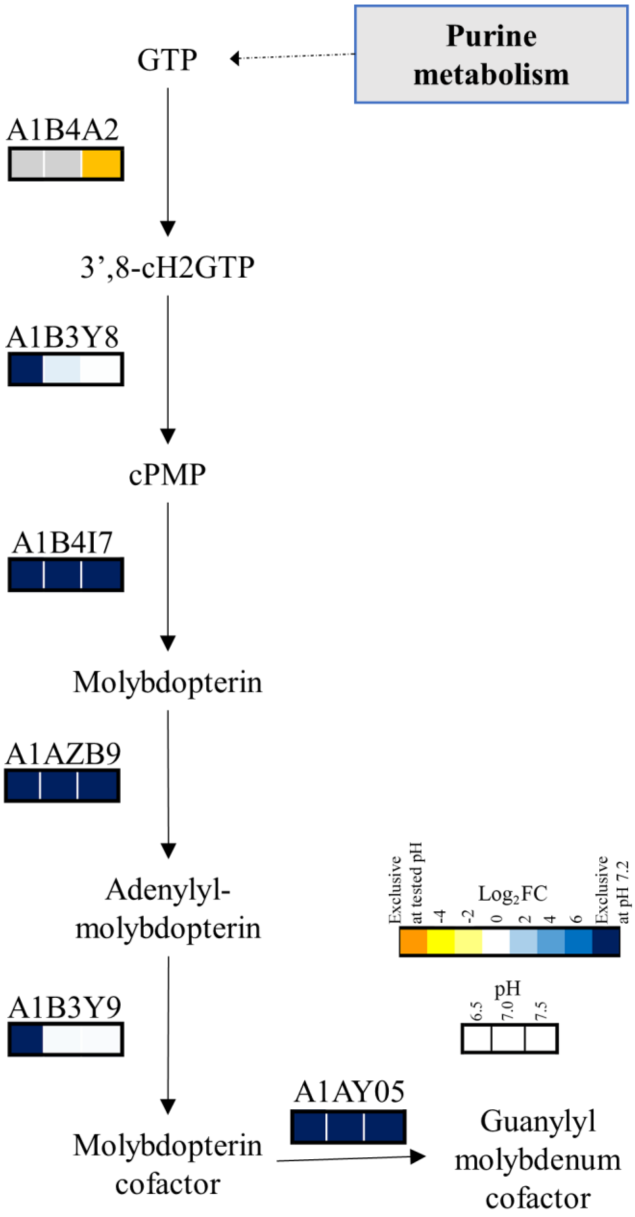
Purine metabolism
GTP
A1B4A2
3',8-cH2GTP
A1B3Y8
cPMP
A1B4I7
Molybdopterin
A1AZB9
Adenylyl-molybdopterin
A1B3Y9
Molybdopterin cofactor
A1AY05
Guanylyl molybdenum cofactor
Exclusive at tested pH
Log2FC
-4
-2
0
2
4
6
Exclusive at pH 7.2
pH
6.5
7.0
7.5

Figure S4

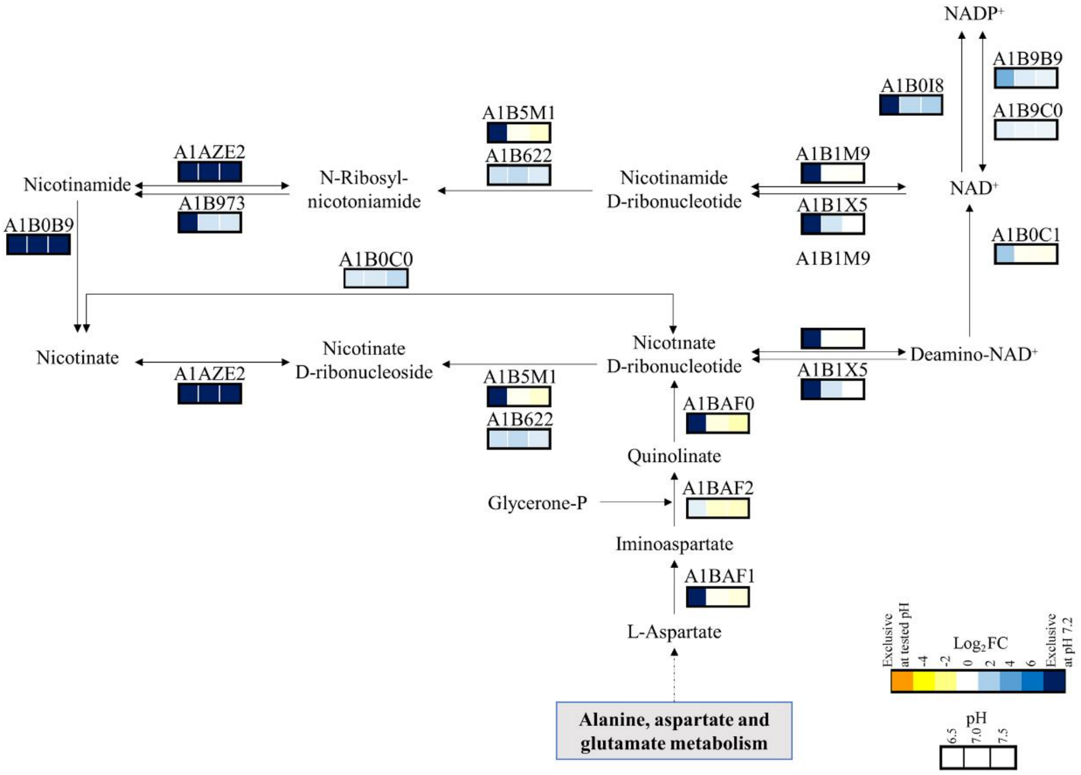

NADP+
A1B9B9
A1B0I8
A1B9C0
A1B5M1
A1B622
A1AZE2
Nicotinamide
N-Ribosyl-
nicotoniamide
Nicotinamide
D-ribonucleotide
A1B1M9
NAD+
A1B973
A1B1X5
A1B0B9
A1B0C1
A1B1M9
A1B0C0
Nicotinate
Nicotinate
D-ribonucleoside
Nicotinate
D-ribonucleotide
Deamino-NAD+
A1AZE2
A1B5M1
A1B1X5
A1BAF0
A1B622
Quinolinate
A1BAF2
Glycerone-P
Iminoaspartate
A1BAF1
L-Aspartate
Alanine, aspartate and
glutamate metabolism
Exclusive
at tested pH
Log2FC
-4
-2
0
2
4
6
Exclusive
at pH 7.2
pH
6.5
7.0
7.5


Figure S5

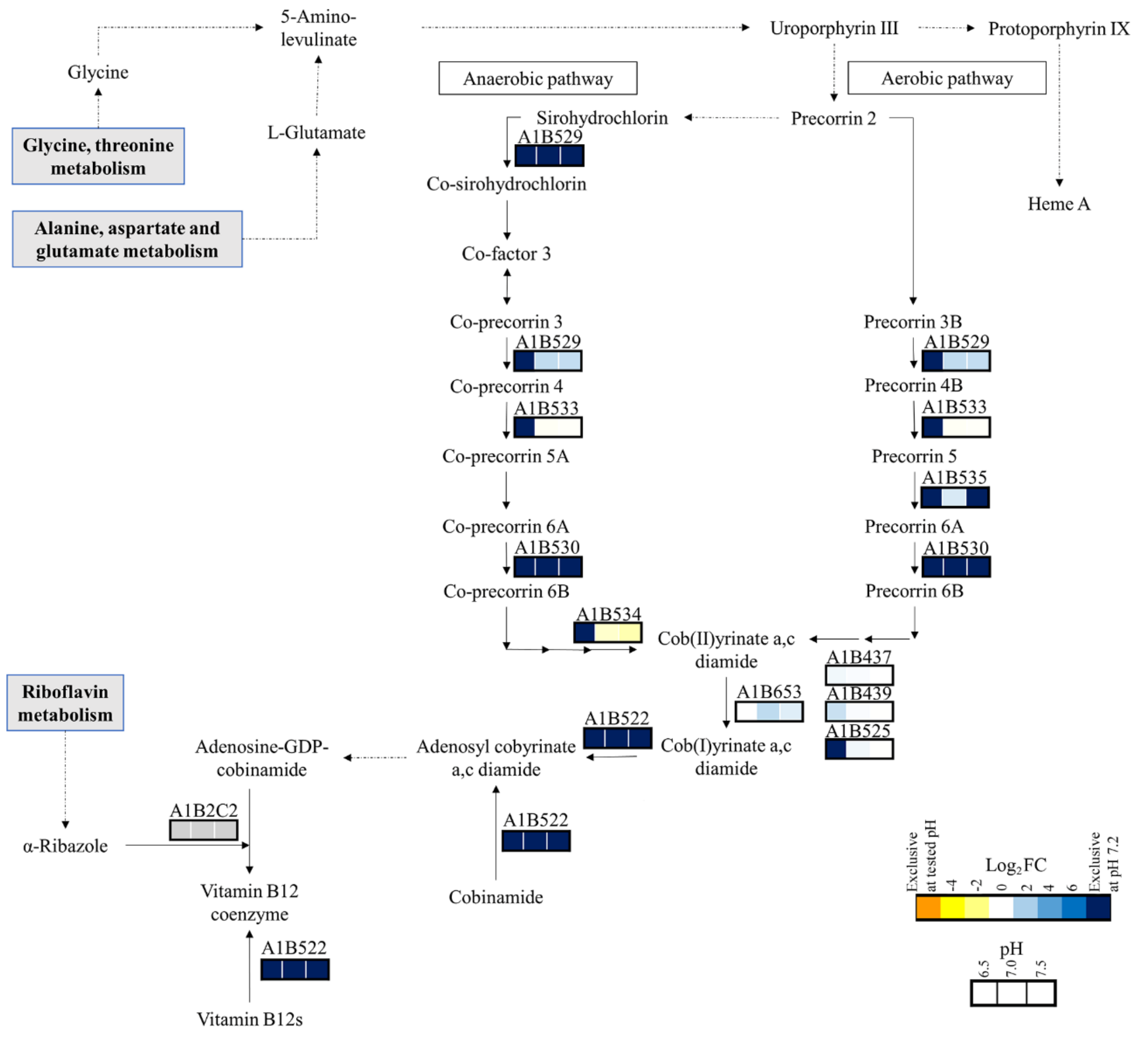

Glycine
5-Amino-levulinate
Uroporphyrin III
Protoporphyrin IX
Glycine, threonine metabolism
L-Glutamate
Alanine, aspartate and glutamate metabolism
Anaerobic pathway
Aerobic pathway
Sirohydrochlorin
Precorrin 2
A1B529
Co-sirohydrochlorin
Heme A
Co-factor 3
Co-precorrin 3
Precorrin 3B
A1B529
A1B529
Co-precorrin 4
Precorrin 4B
A1B533
A1B533
Co-precorrin 5A
Precorrin 5
A1B535
Co-precorrin 6A
Precorrin 6A
A1B530
A1B530
Co-precorrin 6B
Precorrin 6B
A1B534
Cob(II)yrinate a,c diamide
A1B437
A1B653
A1B439
A1B525
Riboflavin metabolism
Adenosine-GDP-cobinamide
A1B522
Adenosyl cobyrinate a,c diamide
Cob(I)yrinate a,c diamide
A1B2C2
α-Ribazole
A1B522
Vitamin B12 coenzyme
Cobinamide
A1B522
Vitamin B12s
Exclusive at tested pH
Log2FC
-4
-2
0
2
4
6
Exclusive at pH 7.2
pH
6.5
7.0
7.5


Figure S6

# REFERENCE

1. Kanehisa, M., Furumichi, M., Sato, Y., Ishiguro-Watanabe, M. & Tanabe, M. KEGG: integrating viruses and cellular organisms. Nucleic Acid Res. **49,** D545-D551. https://doi:10.1093/nar/gkaa970 (2021).
