# Supplementary material for: Effect of pH on the denitrification proteome of the soil bacterium Paracoccus denitrificans PD1222
Source: Sci Rep. 2021 Aug 26;11:17276. doi: 10.1038/s41598-021-96559-2 (PMC8390676; doi:10.1038/s41598-021-96559-2)
Supplement: Supplementary file 2 — Supplementary Information 2. [file 41598_2021_96559_MOESM2_ESM.pdf]

Supplementary material (Tables) to "Effect of pH on the denitrification proteome of the soil bacterium *Paracoccus denitrificans* PD1222"

Alfonso Olaya-Abril

Departamento de Bioquímica y Biología Molecular, Universidad de Córdoba, Edificio Severo Ochoa, 1ª planta, Campus de Rabanales, Córdoba, 14071, Spain

Jesús Hidalgo-Carrillo

Departamento de Química Orgánica, Universidad de Córdoba, Edificio Marie Curie, Campus de Rabanales, Córdoba, 14071, Spain

Víctor M. Luque-Almagro

Departamento de Bioquímica y Biología Molecular, Universidad de Córdoba, Edificio Severo Ochoa, 1ª planta, Campus de Rabanales, Córdoba, 14071, Spain

Carlos Fuentes-Almagro

Servicio Central de Apoyo a la Investigación (SCAI), Unidad de Proteómica, Universidad de Córdoba, Campus de Rabanales, Córdoba, 14071, Spain

Francisco J. Urbano

Departamento de Química Orgánica, Universidad de Córdoba, Edificio Marie Curie, Campus de Rabanales, Córdoba, 14071, Spain

Conrado Moreno-Vivián

Departamento de Bioquímica y Biología Molecular, Universidad de Córdoba, Edificio Severo Ochoa, 1ª planta, Campus de Rabanales, Córdoba, 14071, Spain.

David J. Richardson

School of Biological Sciences, University of East Anglia, Norwich, NR4 7TJ, UK

María Dolores Roldán

Departamento de Bioquímica y Biología Molecular, Universidad de Córdoba, Edificio Severo Ochoa, 1ª planta, Campus de Rabanales, Córdoba, 14071, Spain

This SM provides supplementary information on "proteomic and qPCR data analyses" of the Effect of pH on the Denitrification Proteome of the Soil Bacterium *Paracoccus denitrificans* PD1222.

PROTEOMIC AND qPCR DATA ANALYSES

Table S1. Differential analysis of the *P. denitrificans* proteome by LC-MS/MS at pH 7.2 *versus* pH 6.5 (pH 7.2 was used as reference).

Protein ID[1]	Gene ID[2]	Name	Location[3]	FC[4]
A1B8B1	Pden_3688	Propionyl-CoA carboxylase carboxyltransferase subunit	Cytoplasmic	96.69
A1B9T1	Pden_4211	SSS sodium solute transporter superfamily	Cytoplasmic/Membrane	80.80
A1B4W5	Pden_2472	Carbohydrate ABC transporter substrate-binding protein, CUT1 family	Periplasmic	60.97
A1B9T3	Pden_4213	Acetyl-coenzyme A synthetase (AcCoA synthetase) (Acs) (EC 6.2.1.1) (Acetate-CoA ligase) (Acyl-activating enzyme)	Cytoplasmic	60.80
A1BAR9	Pden_4550	Acetyl-coenzyme A synthetase (AcCoA synthetase) (Acs) (EC 6.2.1.1) (Acetate--CoA ligase) (Acyl-activating enzyme)	Cytoplasmic	60.80
A1B862	Pden_3639	3-methylcrotonoyl-CoA carboxylase, alpha subunit (EC 6.4.1.4)	Cytoplasmic	45.77
A1AZC3	Pden_0505	Mannose-binding protein / fructose-binding protein / ribose-binding protein	Periplasmic	43.29
A1BBI4	Pden_4817	TRAP dicarboxylate transporter, DctP subunit	Periplasmic	42.30
A1B473	Pden_2225	Propionyl-CoA synthetase (EC 6.2.1.17)	Cytoplasmic	29.03
A1B8A7	Pden_3684	Biotin carboxyl carrier protein / biotin carboxylase (EC 6.3.4.14)	Cytoplasmic	25.44
A1B8A4	Pden_3681	Methylmalonyl-CoA mutase (EC 5.4.99.2)	Cytoplasmic	16.08
A1B9P3	Pden_4173	TonB-dependent receptor	OuterMembrane	15.23
A1B350	Pden_1847	Cytochrome c oxidase, cbb3-type, subunit II	Cytoplasmic	15.04
A1BAG7	Pden_4447	Extracellular solute-binding protein, family 1	Periplasmic	14.35
A1B2N7	Pden_1684	Monosaccharide ABC transporter substrate-binding protein, CUT2 family	Periplasmic	13.08
A1B2M4	Pden_1671	Extracellular solute-binding protein, family 5	Periplasmic	12.61
A1B9W4	Pden_4244	Asparaginase (EC 3.5.1.1)	Unknown	10.70
A1AZM6	Pden_0608	Extracellular solute-binding protein, family 5	Periplasmic	10.67
A1B067	Pden_0799	Citrate (Pro-3S)-lyase (EC 4.1.3.6)	Cytoplasmic	10.54
A1B3T6	Pden_2088	Endonuclease	Unknown	10.40
A1B2B9	Pden_1563	TRAP dicarboxylate transporter-DctP subunit	Periplasmic	10.25
A1AZY4	Pden_0716	Amino acid ABC transporter substrate-binding protein, PAAT family	Periplasmic	10.09
A1B8C8	Pden_3705	Amino acid ABC transporter substrate-binding protein, PAAT family	Periplasmic	9.78
A1B3P1	Pden_2043	GcrA cell cycle regulator	Unknown	9.25
A1B6E6	Pden_3007	TonB-dependent siderophore receptor	Outer Membrane	9.02
A1B5F6	Pden_2663	Acetyl-CoA acetyltransferase (EC 2.3.1.9)	Cytoplasmic	8.55
A1B608	Pden_2868	Acyl-CoA dehydrogenase domain protein	Cytoplasmic	8.35
A1B8L7	Pden_3795	Polysaccharide deacetylase	Cytoplasmic	7.78
A1BB82	Pden_4715	TRAP dicarboxylate transporter, DctP subunit	Periplasmic	7.52
A1B347	Pden_1844	4Fe-4S ferredoxin, iron-sulfur binding domain protein	Cytoplasmic/Membrane	7.45
A1B1N5	Pden_1324	Oligopeptide/dipeptide ABC transporter, ATPase subunit	Cytoplasmic/Membrane	7.36
A1B3K2	Pden_2004	Peptidoglycan-binding LysM	Unknown	7.35

A1B4D2	Pden_2285	Substrate-binding region of ABC-type glycine betaine transport system	Periplasmic	6.95
A1B348	Pden_1845	Cbb3-type cytochrome c oxidase subunit CcoP (Cbb3-Cox subunit CcoP) (C-type cytochrome CcoP) (Cyt c(P)) (Cytochrome c oxidase subunit III)	Cytoplasmic	6.87
A1AZK8	Pden_0590	Membrane protein involved in aromatic hydrocarbon degradation	Outer Membrane	6.70
A1B2Y3	Pden_1780	Efflux transporter, RND family, MFP subunit	Cytoplasmic/Membrane	6.61
A1B4Y8	Pden_2495	Cytochrome d1, heme region	Periplasmic	6.61
A1B5H9	Pden_2686	Uncharacterized protein	Periplasmic	6.58
A1B904	Pden_3932	Amino acid/amide ABC transporter substrate-binding protein, HAAT family	Periplasmic	5.67
A1BAK2	Pden_4482	3-hydroxyisobutyrate dehydrogenase (HIBADH) (EC 1.1.1.31)	Cytoplasmic	5.54
A1B2X1	Pden_1768	Acyl carrier protein (ACP)	Cytoplasmic	5.37
A1AZQ1	Pden_0633	Thiamine pyrophosphate enzyme TPP binding domain protein	Cytoplasmic	5.34
A1B0P5	Pden_0978	Uncharacterized protein	Unknown	5.05
A1AZ54	Pden_0434	Acyl-CoA dehydrogenase domain protein	Cytoplasmic	4.93
A1B9U0	Pden_4220	FMN-binding domain protein (NosR)	Cytoplasmic/Membrane	4.85
A1B2K8	Pden_1655	Elongation factor P (EF-P)	Cytoplasmic	4.69
A1B610	Pden_2870	Acetyl-CoA acetyltransferase (EC 2.3.1.9)	Cytoplasmic	4.64
A1B4A0	Pden_2252	UvrABC system protein A (UvrA protein) (Excinuclease ABC subunit A)	Cytoplasmic	4.57
A1B2T9	Pden_1736	Uncharacterized protein	Periplasmic	4.56
A1BA28	Pden_4308	Lipoprotein, YaeC family	Cytoplasmic/Membrane	4.56
A1B4F2	Pden_2305	Ubiquinol-cytochrome c reductase iron-sulfur subunit (EC 1.10.2.2)	Cytoplasmic/Membrane	4.54
A1AZI1	Pden_0563	Citryl-CoA lyase (EC 4.1.3.34)	Cytoplasmic	4.39
A1B989	Pden_4017	Amino acid/amide ABC transporter substrate-binding protein, HAAT family	Unknown	4.32
A1B697	Pden_2957	Oxidoreductase domain protein	Unknown	4.24
A1B3P3	Pden_2045	Methionine--tRNA ligase (EC 6.1.1.10) (Methionyl-tRNA synthetase) (MetRS)	Cytoplasmic	4.20
A1AZ52	Pden_0432	Aa3 type cytochrome c oxidase subunit IV	Cytoplasmic/Membrane	4.19
A1B612	Pden_2872	PfkB domain protein	Cytoplasmic	4.18
A1B1U1	Pden_1384	Ribonucleoside-diphosphate reductase class II (EC 1.17.4.-)	Cytoplasmic	4.05
A1BAE9	Pden_4429	Alpha-1,4 glucan phosphorylase (EC 2.4.1.1)	Cytoplasmic	3.92
A1B0M5	Pden_0958	Poly(R)-hydroxyalkanoic acid synthase, class I	Cytoplasmic	3.87
A1B5Y0	Pden_2840	Acyl-CoA dehydrogenase (EC 1.3.8.-)	Cytoplasmic	3.86
A1B3B6	Pden_1916	Peptidyl-prolyl cis-trans isomerase (PPIase) (EC 5.2.1.8)	Unknown	3.83
A1B5A6	Pden_2613	Formyltetrahydrofolate deformylase (EC 3.5.1.10) (Formyl-FH(4) hydrolase)	Cytoplasmic	3.77
A1B9V5	Pden_4235	Respiratory nitrate reductase beta subunit (NarH)	Cytoplasmic/Membrane	3.75
A1B3Z9	Pden_2151	Polyamine ABC trasnporter, periplasmic polyamine-binding protein	Periplasmic	3.74
A1B9C5	Pden_4053	Alanyl aminopeptidase, Metallo peptidase, MEROPS family M01	Unknown	3.71

A1AZI4	Pden_0566	L-erythro-3-methylmalyl-CoA dehydratase (EC 4.2.1.-)	Cytoplasmic	3.65
A1B3P4	Pden_2046	TonB-dependent receptor	Outer Membrane	3.62
A1B4F4	Pden_2307	Cytochrome c1	Unknown	3.58
A1B400	Pden_2152	Extracellular solute-binding protein, family 1	Periplasmic	3.55
A1B2N0	Pden_1677	Xylose isomerase domain protein TIM barrel	Cytoplasmic	3.55
A1AZF5	Pden_0537	Peptidoglycan-binding domain 1 protein	Cytoplasmic	3.52
A1B3I8	Pden_1990	Uncharacterized protein	Unknown	3.51
A1B076	Pden_0808	Uncharacterized protein	Unknown	3.51
A1B9C3	Pden_4051	Malate synthase G (EC 2.3.3.9)	Cytoplasmic	3.49
A1B595	Pden_2602	Branched chain amino acid aminotransferase apoenzyme (EC 2.6.1.42)	Cytoplasmic	3.48
A1B2M1	Pden_1668	ABC transporter related protein	Cytoplasmic/Membrane	3.48
A1B6A2	Pden_2962	Phosphoribosylformylglycinamidine synthase subunit PurQ (FGAM synthase) (EC 6.3.5.3) (Formylglycinamide ribonucleotide amidotransferase subunit I) (FGAR amidotransferase I) (FGAR-AT I) (Glutaminase PurQ) (EC 3.5.1.2) (Phosphoribosylformylglycinamidine synthase subunit I)	Cytoplasmic	3.47
A1B3Y7	Pden_2139	Indole-3-glycerol phosphate synthase (IGPS) (EC 4.1.1.48)	Cytoplasmic	3.44
A1B0M2	Pden_0955	Threonine--tRNA ligase (EC 6.1.1.3) (Threonyl-tRNA synthetase) (ThrRS)	Cytoplasmic	3.44
A1B1T7	Pden_1380	Uncharacterized protein	Unknown	3.42
A1AYH6	Pden_0206	Short-chain dehydrogenase/reductase SDR	Cytoplasmic	3.41
A1B5H1	Pden_2678	Uncharacterized protein	Unknown	3.39
A1B9V6	Pden_4236	Respiratory nitrate reductase alpha subunit apoprotein (NarG)	Cytoplasmic/Membrane	3.36
A1B2M0	Pden_1667	ABC transporter related protein	Cytoplasmic/Membrane	3.36
A1B911	Pden_3939	Amidophosphoribosyltransferase (ATase) (EC 2.4.2.14) (Glutamine phosphoribosylpyrophosphate amidotransferase) (GPATase)	Cytoplasmic	3.36
Q51700	Pden_2487	Nitrite reductase (EC 1.7.2.1) (Cytochrome cd1) (Cytochrome oxidase) (Hydroxylamine reductase) (EC 1.7.99.1)	Periplasmic	3.33
A1B559	Pden_2566	Uncharacterized protein	Unknown	3.29
A1B8B9	Pden_3696	GMP synthase [glutamine-hydrolyzing] (EC 6.3.5.2) (GMP synthetase) (Glutamine amidotransferase)	Cytoplasmic	3.28
A1B452	Pden_2204	Uncharacterized protein	Unknown	3.24
A1B5M8	Pden_2738	Chromosome partition protein Smc	Cytoplasmic	3.22
A1B9S1	Pden_4201	TonB-dependent heme/hemoglobin receptor family protein	Outer Membrane	3.19
A1B051	Pden_0783	30S ribosomal protein S11	Cytoplasmic	3.16
A1AZV6	Pden_0688	Uncharacterized protein	Unknown	3.08
A1BAG9	Pden_4449	Assimilatory nitrate reductase (NADH) alpha subunit apoprotein (EC 1.7.1.1)	Cytoplasmic	3.06
A1B0K5	Pden_0938	Aminotransferase (EC 2.6.1.-)	Cytoplasmic	3.04
A1B835	Pden_3612	Isoleucine--tRNA ligase (EC 6.1.1.5) (Isoleucyl-tRNA synthetase) (IleRS)	Cytoplasmic	3.03
A1B4Z7	Pden_2504	Uncharacterized protein	Unknown	3.02
A1B4I5	Pden_2338	4-aminobutyrate aminotransferase apoenzyme (EC 2.6.1.19)	Cytoplasmic	3.01

A1AY09	Pden_0036	NlpA lipoprotein	Unknown	2.93
A1B9T8	Pden_4218	Periplasmic copper-binding protein (NosD)	Periplasmic	2.91
A1B2C5	Pden_1569	Extracellular solute-binding protein, family 5	Periplasmic	2.90
A1B611	Pden_2871	3-hydroxyacyl-CoA dehydrogenase (EC 1.1.1.35)	Cytoplasmic	2.89
A1B8N4	Pden_3812	ATP-dependent Clp protease, ATP-binding subunit clpA	Cytoplasmic	2.88
A1B1B9	Pden_1208	Urease subunit alpha (EC 3.5.1.5) (Urea amidohydrolase subunit alpha)	Cytoplasmic	2.88
A1BAD7	Pden_4417	Elongation factor 4 (EF-4) (EC 3.6.5.n1) (Ribosomal back-translocase LepA)	Cytoplasmic/Membrane	2.84
A1B2T8	Pden_1735	Dyp-type peroxidase family	Periplasmic	2.81
A1B9T9	Pden_4219	Nitrous-oxide reductase (EC 1.7.2.4) (N(2)OR) (N2O reductase)	Periplasmic	2.80
A1B4L0	Pden_2364	Alpha-2-macroglobulin domain protein	Unknown	2.77
A1B545	Pden_2552	3-hydroxyacyl-CoA dehydrogenase (EC 1.1.1.35)	Cytoplasmic	2.64
A1B2B8	Pden_1562	Transcriptional repressor NrdR	Cytoplasmic	2.62
A1B1F9	Pden_1248	Extracellular solute-binding protein, family 5	Periplasmic	2.61
A1B8Y2	Pden_3910	Endoribonuclease L-PSP	Cytoplasmic	2.61
A1B943	Pden_3971	Phosphoribosylformylglycinamidine synthase subunit PurL (FGAM synthase) (EC 6.3.5.3) (Formylglycinamide ribonucleotide amidotransferase subunit II) (FGAR amidotransferase II) (FGAR-AT II) (Glutamine amidotransferase PurL) (Phosphoribosylformylglycinamidine synthase subunit II)	Cytoplasmic	2.59
A1B1M5	Pden_1314	Periplasmic glucan biosynthesis protein, MdoG	Periplasmic	2.57
A1BA97	Pden_4377	Ribonucleoside-diphosphate reductase (EC 1.17.4.1)	Cytoplasmic	2.56
A1B1L6	Pden_1305	Antibiotic biosynthesis monooxygenase	Unknown	2.56
A1AZM8	Pden_0610	Amidohydrolase (EC 3.5.1.32)	Cytoplasmic	2.55
A1B467	Pden_2219	Uracil phosphoribosyltransferase (EC 2.4.2.9) (UMP pyrophosphorylase) (UPRTase)	Cytoplasmic	2.54
A1B0B6	Pden_0848	Malate dehydrogenase (Oxaloacetate-decarboxylating) (NADP(+)), Phosphate acetyltransferase (EC 1.1.1.40) (EC 2.3.1.8)	Cytoplasmic	2.52
A1B4A6	Pden_2258	Uncharacterized protein	Unknown	2.51
A1AZD3	Pden_0515	Sarcosine oxidase, alpha subunit family	Cytoplasmic	2.47
A1B4Y7	Pden_2494	Radical SAM domain protein	Cytoplasmic	2.45
A1B9D4	Pden_4062	2-isopropylmalate synthase (EC 2.3.3.13)	Cytoplasmic	2.44
A1B439	Pden_2191	Cobaltochelatase CobT subunit (EC 6.6.1.2)	Cytoplasmic	2.44
A1B092	Pden_0824	Uncharacterized protein	Unknown	2.44
A1B0M7	Pden_0960	Polyhydroxyalkonate synthesis repressor, PhaR	Cytoplasmic	2.43
A1B0B4	Pden_0846	Lipopolysaccharide biosynthesis	Cytoplasmic/Membrane	2.42
A1B2V5	Pden_1752	Coproporphyrinogen oxidase (EC 1.3.3.3)	Cytoplasmic	2.41
A1B1G6	Pden_1255	Short-chain dehydrogenase/reductase SDR	Cytoplasmic	2.40
A1B5A3	Pden_2610	TonB-dependent receptor	OuterMembrane	2.37
A1B1W2	Pden_1405	Uncharacterized protein	Cytoplasmic/Membrane	2.36
A1B3I2	Pden_1984	Dihydroxy-acid dehydratase (DAD) (EC 4.2.1.9)	Cytoplasmic	2.36
A1B8W4	Pden_3892	Pyruvate dehydrogenase E1 component subunit alpha (EC 1.2.4.1)	Cytoplasmic	2.36
A1B2L0	Pden_1657	3-methyl-2-oxobutanoate hydroxymethyltransferase (EC 2.1.2.11) (Ketopantoate hydroxymethyltransferase) (KPHMT)	Cytoplasmic	2.34
A1B2M9	Pden_1676	5-dehydro-2-deoxygluconokinase (EC 2.7.1.92)	Cytoplasmic	2.34

A1AZT2	Pden_0664	TRAP transporter solute receptor, TAXI family	Unknown	2.32
A1B622	Pden_2882	5'-Nucleotidase domain protein	Periplasmic	2.30
A1B513	Pden_2520	3-isopropylmalate dehydratase large subunit (EC 4.2.1.33) (Alpha-IPM isomerase) (IPMI) (Isopropylmalate isomerase)	Cytoplasmic	2.28
A1B4B1	Pden_2263	Uncharacterized protein	Unknown	2.27
A1B0E4	Pden_0877	Membrane protein insertase YidC (Foldase YidC) (Membrane integrase YidC) (Membrane protein YidC)	Cytoplasmic/Membrane	2.26
A1B3V1	Pden_2103	Ureidoglycolate lyase (EC 4.3.2.3)	Cytoplasmic	2.25
A1BA25	Pden_4305	Triosephosphate isomerase (TIM) (TPI) (EC 5.3.1.1) (Triose-phosphate isomerase)	Cytoplasmic	2.25
A1AZX5	Pden_0707	Inositol monophosphatase	Cytoplasmic	2.24
A1B3M5	Pden_2027	3-oxoacyl-[acyl-carrier-protein] reductase (EC 1.1.1.100)	Cytoplasmic	2.23
A1B9U9	Pden_4229	Peptidase U32	Unknown	2.22
A1B9A2	Pden_4030	2-isopropylmalate synthase (EC 2.3.3.13) (Alpha-IPM synthase) (Alpha-isopropylmalate synthase)	Cytoplasmic	2.19
A1B932	Pden_3960	Glutathione synthetase (EC 6.3.2.3) (GSH synthetase) (GSH-S) (GSHase) (Glutathione synthase)	Cytoplasmic	2.18
A1B054	Pden_0786	Transcriptional regulator, LuxR family	Unknown	2.18
A1AZB5	Pden_0497	L-threonine dehydratase (EC 4.3.1.19) (Threonine deaminase)	Cytoplasmic	2.14
A1B001	Pden_0733	Uncharacterized protein	Unknown	2.12
A1B2K4	Pden_1651	Enoyl-[acyl-carrier-protein] reductase [NADH] (EC 1.3.1.9)	Cytoplasmic/Membrane	2.11
A1B1J4	Pden_1283	3-dehydroquinate synthase (EC 4.2.3.4)	Cytoplasmic	2.09
A1B1L5	Pden_1304	Dipeptidase AC, Metallo peptidase, MEROPS family M19	Cytoplasmic	2.08
Q51676	Pden_1851	Oxygen-independent coproporphyrinogen III oxidase (CPO) (EC 1.3.98.3) (Coproporphyrinogen III dehydrogenase) (CPDH)	Cytoplasmic	2.07
A1B2K2	Pden_1649	3-hydroxydecanoyl-[acyl-carrier-protein] dehydratase (EC 4.2.1.59)	Cytoplasmic	2.06
A1B890	Pden_3667	Bifunctional enzyme IspD/IspF [Includes: 2-C-methyl-D-erythritol 4-phosphate cytidylyltransferase (EC 2.7.7.60) (4-diphosphocytidyl-2C-methyl-D-erythritol synthase) (MEP cytidylyltransferase) (MCT); 2-C-methyl-D-erythritol 2,4-cyclodiphosphate synthase (MECDP-synthase) (MECPP-synthase) (MECPS) (EC 4.6.1.12)]	Cytoplasmic	2.03
A1B511	Pden_2518	Iojap-like protein	Cytoplasmic	2.02
A1B1V8	Pden_1401	DNA-directed RNA polymerase subunit omega (RNAP omega subunit) (EC 2.7.7.6) (RNA polymerase omega subunit) (Transcriptase subunit omega)	Cytoplasmic	-2.01
A1BAK7	Pden_4487	Cell division protein FtsZ	Cytoplasmic	-2.02
A1B5G6	Pden_2673	DNA translocase FtsK	Cytoplasmic/Membrane	-2.02
A1B021	Pden_0753	30S ribosomal protein S12	Cytoplasmic	-2.03
A1BAB5	Pden_4395	Sulfate adenylyltransferase subunit 2 (EC 2.7.7.4) (ATP-sulfurylase small subunit) (Sulfate adenylate transferase) (SAT)	Cytoplasmic	-2.06
A1BB79	Pden_4712	Site-determining protein	Cytoplasmic	-2.10
A1B037	Pden_0769	50S ribosomal protein L14	Cytoplasmic	-2.13

A1B502	Pden_2509	RNA pyrophosphohydrolase (EC 3.6.1.-) ((Di)nucleoside polyphosphate hydrolase)	Cytoplasmic	-2.14
A1B9K3	Pden_4133	FHA domain containing protein	Unknown	-2.16
A1BB80	Pden_4713	Probable septum site-determining protein MinC	Cytoplasmic	-2.20
A1B044	Pden_0776	30S ribosomal protein S5	Cytoplasmic	-2.22
A1B937	Pden_3965	UPF0145 protein Pden_3965	Unknown	-2.22
A1B6M1	Pden_3084	Lipoprotein, putative	Unknown	-2.22
A1B1T9	Pden_1382	Protease Do	Periplasmic	-2.23
Q51664	Pden_2482	Protein NorQ	Cytoplasmic	-2.23
A1BAL4	Pden_4494	UDP-N-acetylmuramate--L-alanine ligase (EC 6.3.2.8) (UDP-N-acetylmuramoyl-L-alanine synthetase)	Cytoplasmic	-2.24
A1B013	Pden_0745	50S ribosomal protein L10	Cytoplasmic	-2.33
A1B372	Pden_1869	Sporulation domain protein	Unknown	-2.34
A1BAL5	Pden_4495	UDP-N-acetylglucosamine--N-acetylmuramyl-(pentapeptide) pyrophosphoryl-undecaprenol N-acetylglucosamine transferase (EC 2.4.1.227) (Undecaprenyl-PP-MurNAc-pentapeptide-UDPGlcNAc GlcNAc transferase)	Cytoplasmic/Membrane	-2.35
A1B8U4	Pden_3872	Glutamate dehydrogenase	Unknown	-2.40
A1B053	Pden_0785	50S ribosomal protein L17	Cytoplasmic	-2.51
A1AZG3	Pden_0545	Phosphoglucosamine mutase (EC 5.4.2.10)	Cytoplasmic	-2.55
A1BAB3	Pden_4393	Glycerol-3-phosphate dehydrogenase (EC 1.1.5.3)	Cytoplasmic	-2.67
A1AZG5	Pden_0547	RNA polymerase-binding transcription factor DksA	Cytoplasmic	-2.68
A1B5R6	Pden_2776	Uncharacterized protein	Unknown	-2.72
A1B034	Pden_0766	50S ribosomal protein L16	Cytoplasmic	-2.78
A1BA12	Pden_4292	50S ribosomal protein L33	Cytoplasmic	-2.90
A1AZN6	Pden_0618	Bifunctional protein GlmU [Includes: UDP-N-acetylglucosamine pyrophosphorylase (EC 2.7.7.23) (N-acetylglucosamine-1-phosphate uridyltransferase); Glucosamine-1-phosphate N-acetyltransferase (EC 2.3.1.157)]	Cytoplasmic	-3.10
A1B1U8	Pden_1391	BolA family protein	Unknown	-3.33
A1B082	Pden_0814	D-3-phosphoglycerate dehydrogenase (EC 1.1.1.95)	Cytoplasmic	-3.37
A1BAG4	Pden_4444	Uncharacterized protein	Unknown	-3.37
A1BAN4	Pden_4514	30S ribosomal protein S20	Cytoplasmic	-3.44
A1B081	Pden_0813	Phosphoserine aminotransferase apoenzyme (EC 2.6.1.52)	Cytoplasmic	-3.50
A1BAD6	Pden_4416	Succinate semialdehyde dehydrogenase (EC 1.2.1.16)	Cytoplasmic	-3.72
A1B9I1	Pden_4109	Putative sulfonate/nitrate transport system substrate-binding protein	Cytoplasmic	-3.76
A1B683	Pden_2943	RND efflux system, outer membrane lipoprotein, NodT family	Outer Membrane	-3.84
A1BB78	Pden_4711	Cell division topological specificity factor	Cytoplasmic	-4.18
A1B5Q0	Pden_2760	30S ribosomal protein S15	Cytoplasmic	-4.45
A1B869	Pden_3646	Heat shock protein Hsp20	Cytoplasmic	-4.48
A1B5Z6	Pden_2856	Formate dehydrogenase alpha subunit (EC 1.2.1.2)	Cytoplasmic	-4.52
A1B3D1	Pden_1932	Uncharacterized protein	Periplasmic	-4.55
A1BCE1	Pden_5125	Putative monooxygenase protein	Cytoplasmic	-4.79
A1B0F7	Pden_0890	30S ribosomal protein S18	Cytoplasmic	-5.17
A1B5Z7	Pden_2857	Formate dehydrogenase beta subunit (EC 1.2.1.2)	Cytoplasmic	-6.44
A1BC33	Pden_5017	Uncharacterized protein	Unknown	-6.90
A1B0B7	Pden_0849	Putative outer membrane protein	Outer Membrane	-6.91
A1BC29	Pden_5013	Alkanesulfonate monooxygenase (EC 1.14.14.5)	Cytoplasmic	-7.16

A1B040	Pden_0772	30S ribosomal protein S14	Cytoplasmic	-7.32
A1B8A8	Pden_3685	Uncharacterized protein	Unknown	-7.87
A1B2A7	Pden_1551	Uncharacterized protein	Outer Membrane	-9.04
A1BBD2	Pden_4765	C4-dicarboxylate transport protein	Cytoplasmic/Membrane	-24.26
A1BC23	Pden_5007	Monooxygenase, NtaA/SnaA/SoxA family	Cytoplasmic	-32.69
A1AY28	Pden_0055	50S ribosomal protein L36	Unknown	Exclusive pH 6.5
A1AYK1	Pden_0231	Transcriptional regulator, LysR family	Cytoplasmic	Exclusive pH 6.5
A1B5Z4	Pden_2854	Putative NAD-dependent formate dehydrogenase delta subunit protein	Unknown	Exclusive pH 6.5
A1B681	Pden_2941	Efflux transporter, RND family, MFP subunit	Cytoplasmic/Membrane	Exclusive pH 6.5
A1BC26	Pden_5010	ABC transporter related protein	Cytoplasmic/Membrane	Exclusive pH 6.5
P52221	Pden_1410	Heme exporter protein D (Cytochrome c-type biogenesis protein CcmD)	Unknown	Exclusive pH 6.5
A1AZS4	Pden_0656	Phosphoadenylyl-sulfate reductase (thioredoxin) (EC 1.8.4.8)	Cytoplasmic	Exclusive pH 6.5
A1B6M0	Pden_3083	Putative outer membrane protein	Outer Membrane	Exclusive pH 6.5
A1BC85	Pden_5069	Cystathionine gamma-synthase (EC 2.5.1.48)	Cytoplasmic	Exclusive pH 6.5
A1BCE3	Pden_5127	Transcriptional regulator, Fis family	Unknown	Exclusive pH 6.5
A1AXX4	Pden_0001	Uncharacterized protein	Cytoplasmic/Membrane	Exclusive pH 7.2
A1AXX7	Pden_0004	tRNA uridine 5-carboxymethylaminomethyl modification enzyme MnmG (Glucose-inhibited division protein A)	Cytoplasmic	Exclusive pH 7.2
A1AXX9	Pden_0006	Cobyrinic acid a,c-diamide synthase	Cytoplasmic/Membrane	Exclusive pH 7.2
A1AXY2	Pden_0009	Heat-inducible transcription repressor HrcA	Cytoplasmic/Membrane	Exclusive pH 7.2
A1AXY4	Pden_0011	Non-canonical purine NTP pyrophosphatase (EC 3.6.1.9) (Non-standard purine NTP pyrophosphatase) (Nucleoside-triphosphate diphosphatase) (Nucleoside-triphosphate pyrophosphatase) (NTPase) (Nucleotide diphosphatase)	Cytoplasmic	Exclusive pH 7.2
A1AXY8	Pden_0015	Glutathione-dependent formaldehyde-activating enzyme (EC 4.4.1.22) (S-(hydroxymethyl)glutathione synthase)	Cytoplasmic	Exclusive pH 7.2
A1AXZ1	Pden_0018	GCN5-related N-acetyltransferase	Unknown	Exclusive pH 7.2
A1AXZ3	Pden_0020	Pyrrolo-quinoline quinone	Periplasmic	Exclusive pH 7.2
A1AXZ7	Pden_0024	Uncharacterized protein	Unknown	Exclusive pH 7.2
A1AXZ9	Pden_0026	Amino acid/amide ABC transporter substrate-binding protein, HAAT family	Unknown	Exclusive pH 7.2
A1AY00	Pden_0027	40-residue YVTN family beta-propeller repeat protein	Unknown	Exclusive pH 7.2
A1AY01	Pden_0028	Uncharacterized protein	Unknown	Exclusive pH 7.2
A1AY13	Pden_0040	Thiamine biosynthesis protein ThiS	Unknown	Exclusive pH 7.2

A1AY21	Pden_0048	AMP-dependent synthetase and ligase	Cytoplasmic	Exclusive pH 7.2
A1AY22	Pden_0049	Amino acid/amide ABC transporter ATP-binding protein 1, HAAT family	Cytoplasmic/Membrane	Exclusive pH 7.2
A1AY25	Pden_0052	Amino acid/amide ABC transporter substrate-binding protein, HAAT family	Unknown	Exclusive pH 7.2
A1AY26	Pden_0053	Amino acid/amide ABC transporter ATP-binding protein 2, HAAT family	Cytoplasmic/Membrane	Exclusive pH 7.2
A1AY27	Pden_0054	Phenylacetate-CoA ligase, putative	Cytoplasmic	Exclusive pH 7.2
A1AY32	Pden_0059	Glutathione S-transferase, N-terminal domain	Cytoplasmic/Membrane	Exclusive pH 7.2
A1AY34	Pden_0061	Uncharacterized protein	Unknown	Exclusive pH 7.2
A1AY45	Pden_0072	CHAD domain containing protein	Cytoplasmic	Exclusive pH 7.2
A1AY52	Pden_0079	Two component transcriptional regulator, winged helix family	Unknown	Exclusive pH 7.2
A1AY85	Pden_0112	Uncharacterized protein	Cytoplasmic	Exclusive pH 7.2
A1AYF2	Pden_0180	Aldehyde dehydrogenase	Cytoplasmic	Exclusive pH 7.2
A1AYG0	Pden_0188	Regulatory protein, IclR	Cytoplasmic	Exclusive pH 7.2
A1AYG3	Pden_0191	Glyoxalase/bleomycin resistance protein/dioxygenase	Cytoplasmic	Exclusive pH 7.2
A1AYG5	Pden_0193	TRAP dicarboxylate transporter-DctP subunit	Unknown	Exclusive pH 7.2
A1AYG9	Pden_0197	3-hydroxyacyl-CoA dehydrogenase (EC 1.1.1.35)	Unknown	Exclusive pH 7.2
A1AYK9	Pden_0239	Extracellular solute-binding protein, family 5	Periplasmic	Exclusive pH 7.2
A1AYQ1	Pden_0281	Hydroxyectoine-binding protein / ectoine-binding protein	Periplasmic	Exclusive pH 7.2
A1AYS6	Pden_0306	Uncharacterized protein	Cytoplasmic/Membrane	Exclusive pH 7.2
A1AYS8	Pden_0308	Uncharacterized protein UPF0065	Periplasmic	Exclusive pH 7.2
A1AYS9	Pden_0309	Periplasmic iron-binding protein	Unknown	Exclusive pH 7.2
A1AYU6	Pden_0326	Luciferase family protein	Unknown	Exclusive pH 7.2
A1AYV0	Pden_0330	NADPH-dependent FMN reductase	Cytoplasmic	Exclusive pH 7.2
A1AYV2	Pden_0332	Glutathione S-transferase, N-terminal domain	Cytoplasmic	Exclusive pH 7.2
A1AYV3	Pden_0333	Tryptophan synthase alpha chain (EC 4.2.1.20)	Cytoplasmic	Exclusive pH 7.2
A1AZ13	Pden_0393	Uncharacterized protein	Unknown	Exclusive pH 7.2
A1AZ20	Pden_0400	1-deoxy-D-xylulose-5-phosphate synthase (EC 2.2.1.7) (1-deoxyxylulose-5-phosphate synthase) (DXP synthase) (DXPS)	Cytoplasmic	Exclusive pH 7.2
A1AZ21	Pden_0401	SH3, type 3 domain protein	Cytoplasmic/Membrane	Exclusive pH 7.2
A1AZ24	Pden_0404	AMP nucleosidase (EC 3.2.2.4)	Cytoplasmic	Exclusive pH 7.2
A1AZ40	Pden_0420	Signal peptidase I (EC 3.4.21.89)	Cytoplasmic/Membrane	Exclusive pH 7.2
A1AZ56	Pden_0436	UPF0301 protein Pden_0436	Cytoplasmic	Exclusive pH 7.2

A1AZ59	Pden_0439	Uncharacterized protein	Unknown	Exclusive pH 7.2
A1AZ64	Pden_0444	Uncharacterized protein	Cytoplasmic	Exclusive pH 7.2
A1AZ67	Pden_0447	Uncharacterized protein	Cytoplasmic	Exclusive pH 7.2
A1AZ68	Pden_0448	Uncharacterized protein	Cytoplasmic	Exclusive pH 7.2
A1AZA0	Pden_0482	SpoVT/AbrB domain protein	Cytoplasmic	Exclusive pH 7.2
A1AZA7	Pden_0489	Uncharacterized protein	Unknown	Exclusive pH 7.2
A1AZE1	Pden_0523	tRNA 2-selenouridine synthase (EC 2.9.1.-)	Unknown	Exclusive pH 7.2
A1AZE2	Pden_0524	Purine nucleoside phosphorylase (EC 2.4.2.1) (Inosine-guanosine phosphorylase)	Cytoplasmic	Exclusive pH 7.2
A1AZE5	Pden_0527	Nucleoside ABC transporter ATP-binding protein	Cytoplasmic/Membrane	Exclusive pH 7.2
A1AZE6	Pden_0528	Nucleoside-binding protein	Unknown	Exclusive pH 7.2
A1AZF0	Pden_0532	UspA domain protein	Cytoplasmic	Exclusive pH 7.2
A1AZF9	Pden_0541	Pyruvate phosphate dikinase (EC 2.7.9.1)	Cytoplasmic	Exclusive pH 7.2
A1AZG6	Pden_0548	ATPase associated with various cellular activities, AAA_5	Cytoplasmic	Exclusive pH 7.2
A1AZI3	Pden_0565	Uncharacterized protein	Unknown	Exclusive pH 7.2
A1AZK2	Pden_0584	Uncharacterized protein	Unknown	Exclusive pH 7.2
A1AZK6	Pden_0588	Phospho-N-acetylmuramoyl-pentapeptide-transferase (EC 2.7.8.13) (UDP-MurNAc-pentapeptide phosphotransferase)	Cytoplasmic/Membrane	Exclusive pH 7.2
A1AZM7	Pden_0609	ABC transporter related protein	Cytoplasmic/Membrane	Exclusive pH 7.2
A1AZN5	Pden_0617	HAD-superfamily hydrolase, subfamily IA, variant 3 (EC 3.1.3.18)	Cytoplasmic	Exclusive pH 7.2
A1AZP3	Pden_0625	4-hydroxybenzoate octaprenyltransferase (EC 2.5.1.-) (4-HB polyprenyltransferase)	Cytoplasmic/Membrane	Exclusive pH 7.2
A1AZQ0	Pden_0632	SsrA-binding protein (Small protein B)	Cytoplasmic	Exclusive pH 7.2
A1AZQ6	Pden_0638	Uncharacterized protein	Cytoplasmic/Membrane	Exclusive pH 7.2
A1AZR3	Pden_0645	Peptide deformylase (PDF) (EC 3.5.1.88) (Polypeptide deformylase)	Cytoplasmic	Exclusive pH 7.2
A1AZR4	Pden_0646	Peptide deformylase (PDF) (EC 3.5.1.88) (Polypeptide deformylase)	Cytoplasmic	Exclusive pH 7.2
A1AZR5	Pden_0647	Peptide deformylase (PDF) (EC 3.5.1.88) (Polypeptide deformylase)	Cytoplasmic	Exclusive pH 7.2
A1AZR9	Pden_0651	Transcriptional regulator, AsnC family	Unknown	Exclusive pH 7.2
A1AZS0	Pden_0652	Aminotransferase (EC 2.6.1.-)	Cytoplasmic	Exclusive pH 7.2
A1AZS1	Pden_0653	Transcriptional regulator, AsnC family	Cytoplasmic	Exclusive pH 7.2
A1AZS8	Pden_0660	Gamma-glutamyltransferase 1, Threonine peptidase, MEROPS family T03 (EC 2.3.2.2)	Periplasmic	Exclusive pH 7.2
A1AZT7	Pden_0669	3-oxoacyl-[acyl-carrier-protein] synthase III (EC 2.3.1.41)	Cytoplasmic	Exclusive pH 7.2
A1AZU8	Pden_0680	Transcriptional regulator, GntR family	Cytoplasmic	Exclusive pH 7.2

A1AZU9	Pden_0681	Uncharacterized protein	Unknown	Exclusive pH 7.2
A1AZV0	Pden_0682	4-hydroxybenzoyl-CoA thioesterase	Cytoplasmic	Exclusive pH 7.2
A1AZV3	Pden_0685	Cell division and transport-associated protein TolA	Outer Membrane	Exclusive pH 7.2
A1AZW2	Pden_0694	Protease HtpX homolog (EC 3.4.24.-)	Cytoplasmic/Membrane	Exclusive pH 7.2
A1AZW3	Pden_0695	Holliday junction ATP-dependent DNA helicase RuvA (EC 3.6.4.12)	Cytoplasmic	Exclusive pH 7.2
A1AZW5	Pden_0697	Multisubunit potassium/proton antiporter, PhaB subunit / multisubunit potassium/proton antiporter, PhaA subunit (EC 1.6.99.5)	Cytoplasmic/Membrane	Exclusive pH 7.2
A1AZW7	Pden_0699	Multisubunit potassium/proton antiporter, PhaD subunit (EC 1.6.99.5)	Cytoplasmic/Membrane	Exclusive pH 7.2
A1AZY7	Pden_0719	Amino acid ABC transporter ATP-binding protein, PAAT family	CytoplasmicMembrane	Exclusive pH 7.2
A1AZY9	Pden_0721	Patatin	Cytoplasmic	Exclusive pH 7.2
A1AZZ2	Pden_0724	Transcriptional regulator, LysR family	Cytoplasmic	Exclusive pH 7.2
A1B004	Pden_0736	Glyoxalase/bleomycin resistance protein/dioxygenase	Unknown	Exclusive pH 7.2
A1B045	Pden_0777	50S ribosomal protein L30	Unknown	Exclusive pH 7.2
A1B048	Pden_0780	Protein translocase subunit SecY	Cytoplasmic/Membrane	Exclusive pH 7.2
A1B058	Pden_0790	Pseudouridine synthase (EC 5.4.99.-)	Cytoplasmic	Exclusive pH 7.2
A1B059	Pden_0791	HAD-superfamily hydrolase, subfamily IA, variant 3	Cytoplasmic	Exclusive pH 7.2
A1B062	Pden_0794	L-asparagine ABC transporter membrane protein / L-glutamine ABC transporter membrane protein / L-glutamate ABC transporter membrane protein / L-aspartate ABC transporter membrane protein	Cytoplasmic/Membrane	Exclusive pH 7.2
A1B063	Pden_0795	L-glutamate ABC transporter membrane protein / L-asparagine ABC transporter membrane protein / L-glutamine ABC transporter membrane protein / L-aspartate ABC transporter membrane protein	Cytoplasmic/Membrane	Exclusive pH 7.2
A1B078	Pden_0810	Extracellular solute-binding protein, family 5	Periplasmic	Exclusive pH 7.2
A1B080	Pden_0812	Phosphoserine phosphatase (EC 3.1.3.3)	Cytoplasmic	Exclusive pH 7.2
A1B085	Pden_0817	CreA family protein	Unknown	Exclusive pH 7.2
A1B091	Pden_0823	Uncharacterized protein	Cytoplasmic	Exclusive pH 7.2
A1B094	Pden_0826	20S proteasome, A and B subunits	Cytoplasmic	Exclusive pH 7.2
A1B096	Pden_0828	Hydratase/decarboxylase	Cytoplasmic/Membrane	Exclusive pH 7.2
A1B0A0	Pden_0832	ABC transporter related protein	Cytoplasmic/Membrane	Exclusive pH 7.2
A1B0A6	Pden_0838	Amino acid adenylation domain	Cytoplasmic/Membrane	Exclusive pH 7.2
A1B0B5	Pden_0847	Polysaccharide biosynthesis protein	Cytoplasmic/Membrane	Exclusive pH 7.2
A1B0B8	Pden_0850	UPF0176 protein Pden_0850	Cytoplasmic	Exclusive pH 7.2
A1B0C2	Pden_0854	MORN repeat-containing protein	Periplasmic	Exclusive pH 7.2

A1B0C7	Pden_0859	Cold-shock DNA-binding protein family	Cytoplasmic	Exclusive pH 7.2
A1B0E6	Pden_0879	Probable GTP-binding protein EngB	Unknown	Exclusive pH 7.2
A1B0E9	Pden_0882	Phosphofructokinase	Cytoplasmic	Exclusive pH 7.2
A1B0G0	Pden_0893	Cytochrome-c peroxidase (EC 1.11.1.5)	Unknown	Exclusive pH 7.2
A1B0G4	Pden_0897	Ribosomal RNA small subunit methyltransferase A (EC 2.1.1.182) (16S rRNA (adenine(1518)-N(6)/adenine(1519)-N(6))-dimethyltransferase) (16S rRNA dimethyladenosine transferase) (16S rRNA dimethylase) (S-adenosylmethionine-6-N', N'-adenosyl(rRNA) dimethyltransferase)	Cytoplasmic	Exclusive pH 7.2
A1B0G5	Pden_0898	4-hydroxythreonine-4-phosphate dehydrogenase (EC 1.1.1.262) (4-(phosphohydroxy)-L-threonine dehydrogenase)	Cytoplasmic	Exclusive pH 7.2
A1B0I5	Pden_0918	Alpha/beta hydrolase fold protein	Cytoplasmic	Exclusive pH 7.2
A1B0J4	Pden_0927	Deoxyguanosinetriphosphate triphosphohydrolase-like protein	Cytoplasmic	Exclusive pH 7.2
A1B0J7	Pden_0930	Uncharacterized protein	Cytoplasmic	Exclusive pH 7.2
A1B0J8	Pden_0931	Extracellular solute-binding protein, family 5	Unknown	Exclusive pH 7.2
A1B0K2	Pden_0935	Glyoxalase/bleomycin resistance protein/dioxygenase	Cytoplasmic	Exclusive pH 7.2
A1B0K4	Pden_0937	Uncharacterized protein	Unknown	Exclusive pH 7.2
A1B0L0	Pden_0943	Bifunctional protein PutA	Cytoplasmic	Exclusive pH 7.2
A1B0L2	Pden_0945	Substrate-binding region of ABC-type glycine betaine transport system	Periplasmic	Exclusive pH 7.2
A1B0L7	Pden_0950	Fervidolysin, Serine peptidase, MEROPS family S08A	Cytoplasmic	Exclusive pH 7.2
A1B0L9	Pden_0952	DSBA oxidoreductase	Cytoplasmic	Exclusive pH 7.2
A1B0P2	Pden_0975	Putative phage repressor	Cytoplasmic	Exclusive pH 7.2
A1B0W3	Pden_1046	Transcriptional regulator, LacI family	Cytoplasmic	Exclusive pH 7.2
A1B0W4	Pden_1047	Carbohydrate ABC transporter substrate-binding protein, CUT1 family	Unknown	Exclusive pH 7.2
A1B0X5	Pden_1060	Glyceraldehyde-3-phosphate dehydrogenase, type I (EC 1.2.1.12)	Cytoplasmic	Exclusive pH 7.2
A1B0Y2	Pden_1067	OsmC family protein	Cytoplasmic	Exclusive pH 7.2
A1B121	Pden_1107	Alcohol dehydrogenase GroES domain protein	Cytoplasmic	Exclusive pH 7.2
A1B122	Pden_1108	AMP-dependent synthetase and ligase	Cytoplasmic	Exclusive pH 7.2
A1B194	Pden_1183	Transcriptional regulator, GntR family	Cytoplasmic	Exclusive pH 7.2
A1B1C0	Pden_1209	Urease subunit beta (EC 3.5.1.5) (Urea amidohydrolase subunit beta)	Cytoplasmic	Exclusive pH 7.2
A1B1C7	Pden_1216	Activator of Hsp90 ATPase 1 family protein	Unknown	Exclusive pH 7.2
A1B1E8	Pden_1237	Uncharacterized protein	Unknown	Exclusive pH 7.2
A1B1F1	Pden_1240	Peptidoglycan-binding domain 1 protein	Unknown	Exclusive pH 7.2

A1B1F4	Pden_1243	3-oxoacyl-[acyl-carrier-protein] synthase II (EC 2.3.1.41)	Cytoplasmic/Membrane	Exclusive pH 7.2
A1B1G1	Pden_1250	Binding-protein-dependent transport systems inner membrane component	Cytoplasmic/Membrane	Exclusive pH 7.2
A1B1G2	Pden_1251	ABC transporter related protein	Cytoplasmic/Membrane	Exclusive pH 7.2
A1B1G5	Pden_1254	Aldehyde dehydrogenase	Cytoplasmic	Exclusive pH 7.2
A1B1G9	Pden_1258	ABC transporter related protein	Cytoplasmic/Membrane	Exclusive pH 7.2
A1B1H0	Pden_1259	Periplasmic solute binding protein	Periplasmic	Exclusive pH 7.2
A1B1I4	Pden_1273	Lipid A biosynthesis acyltransferase	Unknown	Exclusive pH 7.2
A1B1I8	Pden_1277	Uracil-xanthine permease	Cytoplasmic/Membrane	Exclusive pH 7.2
A1B1I9	Pden_1278	Uncharacterized protein	Cytoplasmic/Membrane	Exclusive pH 7.2
A1B1J7	Pden_1286	Multisubunit potassium/proton antiporter, PhaE subunit	Cytoplasmic/Membrane	Exclusive pH 7.2
A1B1J8	Pden_1287	Multisubunit potassium/proton antiporter, PhaD subunit	Cytoplasmic/Membrane	Exclusive pH 7.2
A1B1K0	Pden_1289	Multisubunit potassium/proton antiporter, PhaB subunit / multisubunit potassium/proton antiporter, PhaA subunit (EC 1.6.99.5)	Cytoplasmic/Membrane	Exclusive pH 7.2
A1B1K1	Pden_1290	Orn/DAP/Arg decarboxylase 2	Cytoplasmic	Exclusive pH 7.2
A1B1K3	Pden_1292	AMP-dependent synthetase and ligase	Cytoplasmic	Exclusive pH 7.2
A1B1K5	Pden_1294	Uncharacterized protein	Cytoplasmic	Exclusive pH 7.2
A1B1L3	Pden_1302	Polyphosphate kinase (EC 2.7.4.1) (ATP-polyphosphate phosphotransferase) (Polyphosphoric acid kinase)	Cytoplasmic/Membrane	Exclusive pH 7.2
A1B1L8	Pden_1307	Uncharacterized protein	Unknown	Exclusive pH 7.2
A1B1M4	Pden_1313	Uncharacterized protein	Unknown	Exclusive pH 7.2
A1B1N7	Pden_1326	Binding-protein-dependent transport systems inner membrane component	Cytoplasmic/Membrane	Exclusive pH 7.2
A1B1R9	Pden_1362	Periplasmic binding protein	Unknown	Exclusive pH 7.2
A1B1S0	Pden_1363	Isocitrate lyase (EC 4.1.3.1)	Cytoplasmic	Exclusive pH 7.2
A1B1S1	Pden_1364	Malate synthase (EC 2.3.3.9)	Cytoplasmic	Exclusive pH 7.2
A1B1U4	Pden_1387	Inositol monophosphatase	Cytoplasmic	Exclusive pH 7.2
A1B1U7	Pden_1390	Uncharacterized protein	Cytoplasmic/Membrane	Exclusive pH 7.2
A1B1V4	Pden_1397	L-glutamine synthetase (EC 6.3.1.2)	Cytoplasmic	Exclusive pH 7.2
A1B1V7	Pden_1400	(P)ppGpp synthetase I, SpoT/RelA (EC 2.7.6.5)	Cytoplasmic	Exclusive pH 7.2
A1B1Z0	Pden_1433	Biotin synthase 1 (EC 2.8.1.6)	Cytoplasmic	Exclusive pH 7.2
A1B1Z5	Pden_1438	Pathogenesis-related protein	Cytoplasmic	Exclusive pH 7.2
A1B1Z6	Pden_1439	Uncharacterized protein	Cytoplasmic	Exclusive pH 7.2
A1B280	Pden_1524	Putative chromosome segregation SMC protein	Cytoplasmic	Exclusive pH 7.2

A1B281	Pden_1525	Uncharacterized protein	Cytoplasmic/Membrane	Exclusive pH 7.2
A1B282	Pden_1526	Helicase domain protein	Cytoplasmic	Exclusive pH 7.2
A1B283	Pden_1527	DEAD/DEAH box helicase domain protein	Cytoplasmic/Membrane	Exclusive pH 7.2
A1B284	Pden_1528	Uncharacterized protein	Cytoplasmic	Exclusive pH 7.2
A1B2A1	Pden_1545	Histone family protein nucleoid-structuring protein H-NS	Unknown	Exclusive pH 7.2
A1B2B3	Pden_1557	Riboflavin synthase, alpha subunit	Cytoplasmic	Exclusive pH 7.2
A1B2B7	Pden_1561	Diaminohydroxyphosphoribosylaminopyrimidine deaminase / 5-amino-6-(5-phosphoribosylamino)uracil reductase (EC 1.1.1.193) (EC 3.5.4.26)	Cytoplasmic	Exclusive pH 7.2
A1B2C0	Pden_1564	TRAP dicarboxylate transporter, DctM subunit	Cytoplasmic/Membrane	Exclusive pH 7.2
A1B2C1	Pden_1565	Tripartite ATP-independent periplasmic transporter, DctQ component	Cytoplasmic/Membrane	Exclusive pH 7.2
A1B2L1	Pden_1658	Pantothenate synthetase (PS) (EC 6.3.2.1) (Pantoate--beta-alanine ligase) (Pantoate-activating enzyme)	Cytoplasmic	Exclusive pH 7.2
A1B2M3	Pden_1670	Binding-protein-dependent transport systems inner membrane component	Cytoplasmic/Membrane	Exclusive pH 7.2
A1B2M5	Pden_1672	5-deoxyglucuronate isomerase (EC 5.3.1.-)	Cytoplasmic	Exclusive pH 7.2
A1B2M7	Pden_1674	2-keto-myo-inositol dehydratase (EC 4.2.1.44)	Cytoplasmic	Exclusive pH 7.2
A1B2N4	Pden_1681	Monosaccharide ABC transporter substrate-binding protein, CUT2 family	Periplasmic	Exclusive pH 7.2
A1B2S0	Pden_1717	Amino acid/amide ABC transporter substrate-binding protein, HAAT family	Unknown	Exclusive pH 7.2
A1B2S2	Pden_1719	AMP-dependent synthetase and ligase	Cytoplasmic	Exclusive pH 7.2
A1B2S6	Pden_1723	Uncharacterized protein	Unknown	Exclusive pH 7.2
A1B2T2	Pden_1729	Alanine dehydrogenase (EC 1.4.1.1)	Cytoplasmic	Exclusive pH 7.2
A1B2T6	Pden_1733	Iron permease FTR1	Cytoplasmic/Membrane	Exclusive pH 7.2
A1B2T7	Pden_1734	Uncharacterized protein	Periplasmic	Exclusive pH 7.2
A1B2V3	Pden_1750	Transferase hexapeptide repeat containing protein	Cytoplasmic	Exclusive pH 7.2
A1B2V6	Pden_1753	Uncharacterized protein	Unknown	Exclusive pH 7.2
A1B2V8	Pden_1755	16S rRNA m(2)G 1207 methyltransferase (EC 2.1.1.171)	Cytoplasmic	Exclusive pH 7.2
A1B2W5	Pden_1762	DNA-directed DNA polymerase (EC 2.7.7.7)	Cytoplasmic	Exclusive pH 7.2
A1B2W6	Pden_1763	Nucleoside ABC transporter ATP-binding protein	Cytoplasmic/Membrane	Exclusive pH 7.2
A1B2X5	Pden_1772	Beta-lactamase domain protein	Unknown	Exclusive pH 7.2
A1B2Y4	Pden_1781	Transporter, hydrophobe/amphiphile efflux-1 (HAE1) family	Cytoplasmic/Membrane	Exclusive pH 7.2
A1B306	Pden_1803	Glycosyl transferase, group 1	Cytoplasmic	Exclusive pH 7.2
A1B309	Pden_1806	Binding-protein-dependent transport systems inner membrane component	Cytoplasmic/Membrane	Exclusive pH 7.2

A1B313	Pden_1810	Uncharacterized protein	Unknown	Exclusive pH 7.2
A1B324	Pden_1821	Uncharacterized protein	Cytoplasmic	Exclusive pH 7.2
A1B336	Pden_1833	Peptidase M19, renal dipeptidase	Unknown	Exclusive pH 7.2
A1B337	Pden_1834	Glutathione S-transferase, C-terminal domain	Cytoplasmic	Exclusive pH 7.2
A1B343	Pden_1840	Lipoprotein releasing system, transmembrane protein, LolC/E family	Cytoplasmic/Membrane	Exclusive pH 7.2
A1B345	Pden_1842	Heavy metal translocating P-type ATPase	Cytoplasmic/Membrane	Exclusive pH 7.2
A1B346	Pden_1843	FixH family protein	Cytoplasmic/Membrane	Exclusive pH 7.2
A1B351	Pden_1848	Cytochrome c oxidase, cbb3-type, subunit I	Cytoplasmic/Membrane	Exclusive pH 7.2
A1B360	Pden_1857	Glycosyl transferase, family 14	Cytoplasmic	Exclusive pH 7.2
A1B361	Pden_1858	Uncharacterized protein	Cytoplasmic	Exclusive pH 7.2
A1B362	Pden_1859	50S ribosomal protein L32	Cytoplasmic	Exclusive pH 7.2
A1B368	Pden_1865	2-deoxycytidine 5-triphosphate deaminase	Cytoplasmic	Exclusive pH 7.2
A1B369	Pden_1866	Segregation and condensation protein B	Cytoplasmic	Exclusive pH 7.2
A1B385	Pden_1884	Imidazole glycerol phosphate synthase subunit HisH (EC 2.4.2.-) (IGP synthase glutamine amidotransferase subunit) (IGP synthase subunit HisH) (ImGP synthase subunit HisH) (IGPS subunit HisH)	Cytoplasmic	Exclusive pH 7.2
A1B388	Pden_1887	Imidazole glycerol phosphate synthase subunit HisF (EC 4.1.3.-) (IGP synthase cyclase subunit) (IGP synthase subunit HisF) (ImGP synthase subunit HisF) (IGPS subunit HisF)	Cytoplasmic	Exclusive pH 7.2
A1B391	Pden_1890	Ribonuclease D (RNase D) (EC 3.1.13.5)	Cytoplasmic	Exclusive pH 7.2
A1B395	Pden_1894	Uncharacterized protein	Unknown	Exclusive pH 7.2
A1B3A0	Pden_1899	Substrate-binding region of ABC-type glycine betaine transport system	Unknown	Exclusive pH 7.2
A1B3A2	Pden_1901	ABC transporter related protein	Cytoplasmic/Membrane	Exclusive pH 7.2
A1B3B2	Pden_1912	Uncharacterized protein	Unknown	Exclusive pH 7.2
A1B3C4	Pden_1924	ATP phosphoribosyltransferase regulatory subunit (EC 2.4.2.17)	Cytoplasmic	Exclusive pH 7.2
A1B3D6	Pden_1937	Cytochrome c, class I	Periplasmic	Exclusive pH 7.2
A1B3D7	Pden_1938	Cytochrome c oxidase subunit 1 (EC 1.9.3.1)	Cytoplasmic/Membrane	Exclusive pH 7.2
A1B3E7	Pden_1949	Putative L-sorbosone dehydrogenase	Cytoplasmic/Membrane	Exclusive pH 7.2
A1B3E8	Pden_1950	Glucose-6-phosphate isomerase (GPI) (EC 5.3.1.9) (Phosphoglucose isomerase) (PGI) (Phosphohexose isomerase) (PHI)	Cytoplasmic	Exclusive pH 7.2
A1B3E9	Pden_1951	6-phosphogluconolactonase (EC 3.1.1.31)	Cytoplasmic	Exclusive pH 7.2
A1B3F0	Pden_1952	Glucose-6-phosphate 1-dehydrogenase (G6PD) (EC 1.1.1.49)	Cytoplasmic	Exclusive pH 7.2

A1B3F6	Pden_1958	UPF0262 protein Pden_1958	Cytoplasmic	Exclusive pH 7.2
A1B3F8	Pden_1960	Uncharacterized protein	Cytoplasmic	Exclusive pH 7.2
A1B3G1	Pden_1963	Plasmid maintenance system antidote protein, XRE family	Unknown	Exclusive pH 7.2
A1B3I0	Pden_1982	Uncharacterized protein	Unknown	Exclusive pH 7.2
A1B3I6	Pden_1988	FolC bifunctional protein (EC 6.3.2.17)	Cytoplasmic	Exclusive pH 7.2
A1B3I7	Pden_1989	AFG1-family ATPase	Cytoplasmic	Exclusive pH 7.2
A1B3J6	Pden_1998	tRNA N6-adenosine threonylcarbamoyltransferase (EC 2.3.1.234) (N6-L-threonylcarbamoyladenine synthase) (t(6)A synthase) (t(6)A37 threonylcarbamoyladenosine biosynthesis protein TsaD) (tRNA threonylcarbamoyladenosine biosynthesis protein TsaD)	Extracellular	Exclusive pH 7.2
A1B3J7	Pden_1999	Glycerol-3-phosphate dehydrogenase [NAD(P)+] (EC 1.1.1.94) (NAD(P)H-dependent glycerol-3-phosphate dehydrogenase)	Unknown	Exclusive pH 7.2
A1B3J8	Pden_2000	YCII-related protein	Unknown	Exclusive pH 7.2
A1B3K7	Pden_2009	Sulfotransferase	Cytoplasmic	Exclusive pH 7.2
A1B3K8	Pden_2010	Ribosomal protein S12 methylthiotransferase RimO (S12 MTTase) (S12 methylthiotransferase) (EC 2.8.4.4) (Ribosomal protein S12 (aspartate-C(3))-methylthiotransferase) (Ribosome maturation factor RimO)	Cytoplasmic	Exclusive pH 7.2
A1B3L3	Pden_2015	1-acyl-sn-glycerol-3-phosphate acyltransferase (EC 2.3.1.51)	Cytoplasmic/Membrane	Exclusive pH 7.2
A1B3L6	Pden_2018	MJ0042 family finger-like protein	Unknown	Exclusive pH 7.2
A1B3M1	Pden_2023	Redoxin domain protein	Cytoplasmic/Membrane	Exclusive pH 7.2
A1B3M6	Pden_2028	Uncharacterized protein	Unknown	Exclusive pH 7.2
A1B3N2	Pden_2034	Toluene tolerance family protein	Unknown	Exclusive pH 7.2
A1B3N4	Pden_2036	Type I secretion system ATPase	Cytoplasmic/Membrane	Exclusive pH 7.2
A1B3N5	Pden_2037	Type I secretion membrane fusion protein, HlyD family	Cytoplasmic/Membrane	Exclusive pH 7.2
A1B3T3	Pden_2085	Uncharacterized protein	Cytoplasmic	Exclusive pH 7.2
A1B3T7	Pden_2089	Uncharacterized protein	Cytoplasmic	Exclusive pH 7.2
A1B3U6	Pden_2098	Alkane 1-monooxygenase (EC 1.14.15.3)	Cytoplasmic/Membrane	Exclusive pH 7.2
A1B3W7	Pden_2119	Glucose-1-phosphate thymidylyltransferase (EC 2.7.7.24)	Cytoplasmic	Exclusive pH 7.2
A1B3X6	Pden_2128	Uncharacterized protein	Unknown	Exclusive pH 7.2
A1B3Y2	Pden_2134	Aminotransferase (EC 2.6.1.-)	Cytoplasmic	Exclusive pH 7.2
A1B3Y8	Pden_2140	Cyclic pyranopterin monophosphate synthase accessory protein (Molybdenum cofactor biosynthesis protein C)	Cytoplasmic	Exclusive pH 7.2
A1B3Y9	Pden_2141	Molybdopterin molybdochelatase	Cytoplasmic	Exclusive pH 7.2

A1B3Z2	Pden_2144	Thymidylate kinase (EC 2.7.4.9) (dTMP kinase)	Cytoplasmic	Exclusive pH 7.2
A1B3Z7	Pden_2149	Binding-protein-dependent transport systems inner membrane component	Cytoplasmic/Membrane	Exclusive pH 7.2
A1B411	Pden_2163	Alcohol dehydrogenase GroES domain protein	Cytoplasmic	Exclusive pH 7.2
A1B412	Pden_2164	GatB/Yqey domain protein	Cytoplasmic	Exclusive pH 7.2
A1B415	Pden_2167	Glutamate-ammonia-ligase adenylyltransferase (EC 2.7.7.42)	Cytoplasmic	Exclusive pH 7.2
A1B423	Pden_2175	Nitroreductase	Cytoplasmic	Exclusive pH 7.2
A1B428	Pden_2180	GCN5-related N-acetyltransferase	Unknown	Exclusive pH 7.2
A1B436	Pden_2188	Uncharacterized protein	Cytoplasmic/Membrane	Exclusive pH 7.2
A1B440	Pden_2192	Glycosyl transferase, group 1	Cytoplasmic	Exclusive pH 7.2
A1B445	Pden_2197	Export-related chaperone CsaA	Cytoplasmic	Exclusive pH 7.2
A1B453	Pden_2205	Heat shock protein Hsp20	Cytoplasmic	Exclusive pH 7.2
A1B457	Pden_2209	Transcriptional regulator, AsnC family	Cytoplasmic	Exclusive pH 7.2
A1B463	Pden_2215	Ribosomal large subunit pseudouridine synthase D (EC 5.4.99.-)	Cytoplasmic	Exclusive pH 7.2
A1B465	Pden_2217	Uncharacterized protein	Cytoplasmic	Exclusive pH 7.2
A1B470	Pden_2222	Thymidine phosphorylase (EC 2.4.2.4)	Unknown	Exclusive pH 7.2
A1B471	Pden_2223	Cytidine deaminase (EC 3.5.4.5)	Cytoplasmic	Exclusive pH 7.2
A1B478	Pden_2230	Biotin--acetyl-CoA-carboxylase ligase (EC 6.3.4.15)	Cytoplasmic	Exclusive pH 7.2
A1B479	Pden_2231	NADH-quinone oxidoreductase subunit N (EC 1.6.5.11) (NADH dehydrogenase I subunit 14)	Cytoplasmic/Membrane	Exclusive pH 7.2
A1B480	Pden_2232	NADH dehydrogenase subunit M (EC 1.6.5.3)	Cytoplasmic/Membrane	Exclusive pH 7.2
A1B482	Pden_2234	NADH-quinone oxidoreductase subunit K (EC 1.6.5.11) (NADH dehydrogenase I subunit K)	Cytoplasmic/Membrane	Exclusive pH 7.2
A1B485	Pden_2237	Carboxymuconolactone decarboxylase	Unknown	Exclusive pH 7.2
A1B487	Pden_2239	NADH-quinone oxidoreductase subunit H (EC 1.6.5.11) (NADH dehydrogenase I subunit 8)	Cytoplasmic/Membrane	Exclusive pH 7.2
A1B492	Pden_2244	Uncharacterized protein	Cytoplasmic/Membrane	Exclusive pH 7.2
A1B4A1	Pden_2253	Glutathione-dependent formaldehyde-activating, GFA	Unknown	Exclusive pH 7.2
A1B4B7	Pden_2269	Alcohol dehydrogenase, zinc-binding domain protein	Cytoplasmic	Exclusive pH 7.2
A1B4D3	Pden_2286	Sulfatase	Cytoplasmic	Exclusive pH 7.2

A1B4D4	Pden_2287	Membrane protein of uknown function UCP014873	Cytoplasmic/Membrane	Exclusive pH 7.2
A1B4D6	Pden_2289	Glutathione S-transferase, N-terminal domain	Cytoplasmic	Exclusive pH 7.2
A1B4E3	Pden_2296	UDP-galactose 4-epimerase (EC 5.1.3.2)	Cytoplasmic	Exclusive pH 7.2
A1B4F3	Pden_2306	Cytochrome b	Cytoplasmic/Membrane	Exclusive pH 7.2
A1B4F5	Pden_2308	Ribokinase (RK) (EC 2.7.1.15)	Cytoplasmic	Exclusive pH 7.2
A1B4F6	Pden_2309	Peptidase S49	Cytoplasmic/Membrane	Exclusive pH 7.2
A1B4G2	Pden_2315	Mammalian cell entry related domain protein	Unknown	Exclusive pH 7.2
A1B4G8	Pden_2321	Orn/DAP/Arg decarboxylase 2	Cytoplasmic	Exclusive pH 7.2
A1B4G9	Pden_2322	Transcriptional regulator, AsnC family	Cytoplasmic	Exclusive pH 7.2
A1B4H9	Pden_2332	Cobalamin (Vitamin B12) biosynthesis CbiX protein	Cytoplasmic	Exclusive pH 7.2
A1B4J4	Pden_2348	Aminomethyltransferase (EC 2.1.2.10)	Cytoplasmic	Exclusive pH 7.2
A1B4J9	Pden_2353	Uncharacterized protein	Cytoplasmic	Exclusive pH 7.2
A1B4K0	Pden_2354	Two component transcriptional regulator, LuxR family	Cytoplasmic	Exclusive pH 7.2
A1B4M9	Pden_2383	Isochorismatase (EC 3.3.2.1)	Cytoplasmic	Exclusive pH 7.2
A1B4S4	Pden_2431	LAO/AO transport system ATPase	Cytoplasmic/Membrane	Exclusive pH 7.2
A1B4T4	Pden_2441	Serine/threonine protein kinase	Cytoplasmic	Exclusive pH 7.2
A1B4T7	Pden_2444	Uncharacterized protein	Cytoplasmic	Exclusive pH 7.2
A1B4W2	Pden_2469	Carbohydrate ABC transporter ATP-binding protein, CUT1 family	Cytoplasmic/Membrane	Exclusive pH 7.2
A1B4W6	Pden_2473	Uncharacterized protein	Unknown	Exclusive pH 7.2
A1B4W9	Pden_2476	Glutamine--fructose-6-phosphate transaminase (EC 2.6.1.16)	Cytoplasmic	Exclusive pH 7.2
A1B4X1	Pden_2478	Putative transcriptional regulator, Crp/Fnr family	Cytoplasmic	Exclusive pH 7.2
A1B4Z3	Pden_2500	Uncharacterized protein	Cytoplasmic/Membrane	Exclusive pH 7.2
A1B4Z8	Pden_2505	Dual-specificity RNA methyltransferase RlmN (EC 2.1.1.192) (23S rRNA (adenine(2503)-C(2))-methyltransferase) (23S rRNA m2A2503 methyltransferase) (Ribosomal RNA large subunit methyltransferase N) (tRNA (adenine(37)-C(2))-methyltransferase) (tRNA m2A37 methyltransferase)	Cytoplasmic	Exclusive pH 7.2
A1B505	Pden_2512	YbaK/prolyl-tRNA synthetase associated region	Unknown	Exclusive pH 7.2
A1B522	Pden_2529	Cob(I)yrinic acid a,c-diamide adenosyltransferase (EC 2.5.1.17)	Cytoplasmic	Exclusive pH 7.2
A1B525	Pden_2532	Cobaltochelatase CobN subunit (EC 6.6.1.2)	Cytoplasmic	Exclusive pH 7.2
A1B529	Pden_2536	Precorrin-3 methyltransferase (EC 2.1.1.131)	Cytoplasmic	Exclusive pH 7.2
A1B530	Pden_2537	Precorrin-6A reductase (EC 1.3.1.54)	Cytoplasmic	Exclusive pH 7.2
A1B533	Pden_2540	Precorrin-4 C11-methyltransferase (EC 2.1.1.133)	Cytoplasmic	Exclusive pH 7.2

A1B537	Pden_2544	Cytochrome c, class II	Periplasmic	Exclusive pH 7.2
A1B548	Pden_2555	Aspartate carbamoyltransferase (EC 2.1.3.2) (Aspartate transcarbamylase) (ATCase)	Cytoplasmic	Exclusive pH 7.2
A1B560	Pden_2567	Periplasmic binding protein	Unknown	Exclusive pH 7.2
A1B571	Pden_2578	Uncharacterized protein	Unknown	Exclusive pH 7.2
A1B586	Pden_2593	Uncharacterized protein	Unknown	Exclusive pH 7.2
A1B593	Pden_2600	Two component transcriptional regulator, winged helix family	Cytoplasmic	Exclusive pH 7.2
A1B599	Pden_2606	Lipopolysaccharide export system protein LptA	Unknown	Exclusive pH 7.2
A1B5A1	Pden_2608	KpsF/GutQ family protein (EC 5.3.1.13)	Unknown	Exclusive pH 7.2
A1B5B3	Pden_2620	Kynureninase (EC 3.7.1.3) (L-kynurenine hydrolase)	Cytoplasmic	Exclusive pH 7.2
A1B5B5	Pden_2622	Uncharacterized protein	Unknown	Exclusive pH 7.2
A1B5B6	Pden_2623	Uncharacterized protein	Unknown	Exclusive pH 7.2
A1B5E6	Pden_2653	Efflux transporter, RND family, MFP subunit	Unknown	Exclusive pH 7.2
A1B5E7	Pden_2654	Efflux transporter, RND family, MFP subunit	Unknown	Exclusive pH 7.2
A1B5E8	Pden_2655	Acriflavin resistance protein	Cytoplasmic/Membrane	Exclusive pH 7.2
A1B5F0	Pden_2657	Uncharacterized protein	Cytoplasmic	Exclusive pH 7.2
A1B5F2	Pden_2659	Murein endopeptidase, Metallo peptidase, MEROPS family M74	Periplasmic	Exclusive pH 7.2
A1B5F7	Pden_2664	Putative transcriptional regulator, ModE family	Unknown	Exclusive pH 7.2
A1B5G2	Pden_2669	Outer-membrane lipoprotein carrier protein	Unknown	Exclusive pH 7.2
A1B5G3	Pden_2670	2-octaprenyl-3-methyl-6-methoxy-1,4-benzoquinol hydroxylase / 2-octaprenyl-6-methoxyphenol hydroxylase (EC 1.14.13.-)	Cytoplasmic/Membrane	Exclusive pH 7.2
A1B5G8	Pden_2675	Secretion protein HlyD family protein	Cytoplasmic/Membrane	Exclusive pH 7.2
A1B5I2	Pden_2689	Flavin reductase domain protein, FMN-binding protein	Unknown	Exclusive pH 7.2
A1B5I5	Pden_2692	Mammalian cell entry related domain protein	Unknown	Exclusive pH 7.2
A1B5K9	Pden_2719	Heparinase II/III family protein	Cytoplasmic	Exclusive pH 7.2
A1B5L0	Pden_2720	Fmu (Sun) domain protein	Cytoplasmic	Exclusive pH 7.2
A1B5L1	Pden_2721	Efflux transporter, RND family, MFP subunit	Cytoplasmic/Membrane	Exclusive pH 7.2
A1B5L2	Pden_2722	Transporter, hydrophobe/amphiphile efflux-1 (HAE1) family	Cytoplasmic/Membrane	Exclusive pH 7.2
A1B5L5	Pden_2725	Uncharacterized protein	Cytoplasmic	Exclusive pH 7.2
A1B5M0	Pden_2730	Protein-L-isoaspartate O-methyltransferase (EC 2.1.1.77) (L-isoaspartyl protein carboxyl methyltransferase) (Protein L-isoaspartyl methyltransferase) (Protein-beta-aspartate methyltransferase) (PIMT)	Cytoplasmic	Exclusive pH 7.2
A1B5M1	Pden_2731	5'-nucleotidase SurE (EC 3.1.3.5) (Nucleoside 5'-monophosphate phosphohydrolase)	Cytoplasmic	Exclusive pH 7.2

A1B5M5	Pden_2735	Glycerophosphoryl diester phosphodiesterase	Cytoplasmic	Exclusive pH 7.2
A1B5N2	Pden_2742	Uncharacterized protein	Unknown	Exclusive pH 7.2
A1B5N5	Pden_2745	Multiple monosaccharide-binding protein	Periplasmic	Exclusive pH 7.2
A1B5Q2	Pden_2762	Alanine racemase domain protein	Cytoplasmic	Exclusive pH 7.2
A1B5Q9	Pden_2769	Glutathione S-transferase, N-terminal domain	Cytoplasmic	Exclusive pH 7.2
A1B5R1	Pden_2771	5'-nucleotidase, lipoprotein e(P4) family	Outer Membrane	Exclusive pH 7.2
A1B5S5	Pden_2785	FxsA cytoplasmic membrane protein	Cytoplasmic/Membrane	Exclusive pH 7.2
A1B5T1	Pden_2791	ATP-dependent protease subunit HslV (EC 3.4.25.2)	Cytoplasmic	Exclusive pH 7.2
A1B5T7	Pden_2797	Uncharacterized protein	Cytoplasmic	Exclusive pH 7.2
A1B5U2	Pden_2802	Uncharacterized protein	Unknown	Exclusive pH 7.2
A1B5V2	Pden_2812	Transcriptional regulator, BadM/Rrf2 family	Cytoplasmic	Exclusive pH 7.2
A1B5W1	Pden_2821	Uncharacterized protein	Unknown	Exclusive pH 7.2
A1B5W6	Pden_2826	Protein FdhE homolog	Cytoplasmic	Exclusive pH 7.2
A1B5X3	Pden_2833	Chromosomal replication initiator protein DnaA	Cytoplasmic	Exclusive pH 7.2
A1B5Z0	Pden_2850	Nucleotide-binding protein Pden_2850	Cytoplasmic	Exclusive pH 7.2
A1B5Z3	Pden_2853	Ribonuclease R (RNase R) (EC 3.1.13.1)	Cytoplasmic	Exclusive pH 7.2
A1B607	Pden_2867	Transcriptional regulator, MerR family	Cytoplasmic	Exclusive pH 7.2
A1B619	Pden_2879	ATP synthase subunit a (ATP synthase F0 sector subunit a) (F-ATPase subunit 6)	Cytoplasmic/Membrane	Exclusive pH 7.2
A1B645	Pden_2905	Short chain enoyl-CoA hydratase (EC 4.2.1.17)	Cytoplasmic	Exclusive pH 7.2
A1B647	Pden_2907	Acetyl-CoA acetyltransferase (EC 2.3.1.9)	Cytoplasmic	Exclusive pH 7.2
A1B648	Pden_2908	Short-chain dehydrogenase/reductase SDR	Unknown	Exclusive pH 7.2
A1B649	Pden_2909	AMP-dependent synthetase and ligase	Cytoplasmic	Exclusive pH 7.2
A1B653	Pden_2913	Cobyrinic acid a,c-diamide synthase	Cytoplasmic	Exclusive pH 7.2
A1B658	Pden_2918	ATP-dependent dethiobiotin synthetase BioD (EC 6.3.3.3) (DTB synthetase) (DTBS) (Dethiobiotin synthase)	Cytoplasmic	Exclusive pH 7.2
A1B690	Pden_2950	Uncharacterized protein	Unknown	Exclusive pH 7.2
A1B693	Pden_2953	Transcriptional regulator, AraC family with amidase-like domain	Cytoplasmic/Membrane	Exclusive pH 7.2
A1B696	Pden_2956	Carboxynorspermidine/carboxyspermidine decarboxylase (CANS DC/CAS DC) (CANSDC/CASDC) (EC 4.1.1.96)	Cytoplasmic	Exclusive pH 7.2
A1B699	Pden_2959	Uncharacterized protein	Unknown	Exclusive pH 7.2
A1B6A3	Pden_2963	Integral membrane sensor signal transduction histidine kinase	Cytoplasmic/Membrane	Exclusive pH 7.2
A1B6A7	Pden_2967	Enoyl-CoA hydratase/isomerase	Cytoplasmic	Exclusive pH 7.2

A1B6B9	Pden_2980	Periplasmic binding protein	Unknown	Exclusive pH 7.2
A1B6C8	Pden_2989	Anaerobic ribonucleoside-triphosphate reductase	Cytoplasmic	Exclusive pH 7.2
A1B6E4	Pden_3005	Uncharacterized protein	Unknown	Exclusive pH 7.2
A1B6I4	Pden_3047	Transcriptional regulator, MarR family	Cytoplasmic	Exclusive pH 7.2
A1B718	Pden_3231	Putative transcriptional regulator, IclR family	Cytoplasmic	Exclusive pH 7.2
A1B745	Pden_3260	Aldehyde dehydrogenase	Cytoplasmic	Exclusive pH 7.2
A1B750	Pden_3266	Uncharacterized protein UPF0065	Unknown	Exclusive pH 7.2
A1B751	Pden_3267	Amidohydrolase 2	Cytoplasmic	Exclusive pH 7.2
A1B7G8	Pden_3389	Transcriptional regulator, GntR family	Cytoplasmic	Exclusive pH 7.2
A1B7I1	Pden_3404	Carbohydrate ABC transporter substrate-binding protein, CUT1 family	Periplasmic	Exclusive pH 7.2
A1B7L4	Pden_3437	Fumarylacetoacetate (FAA) hydrolase	Cytoplasmic	Exclusive pH 7.2
A1B7R6	Pden_3490	4-hydroxybenzoate 3-monooxygenase (EC 1.14.13.2)	Cytoplasmic	Exclusive pH 7.2
A1B7S3	Pden_3497	TRAP dicarboxylate transporter-DctP subunit	Periplasmic	Exclusive pH 7.2
A1B7V3	Pden_3527	TonB-dependent receptor	Outer Membrane	Exclusive pH 7.2
A1B7V4	Pden_3528	Transcriptional regulator, IclR family	Cytoplasmic	Exclusive pH 7.2
A1B7V6	Pden_3530	Siderophore-interacting protein	Cytoplasmic	Exclusive pH 7.2
A1B7V7	Pden_3531	Periplasmic binding protein	Unknown	Exclusive pH 7.2
A1B7Z3	Pden_3569	Formamidase (EC 3.5.1.49)	Cytoplasmic	Exclusive pH 7.2
A1B810	Pden_3586	Transglutaminase domain protein	Unknown	Exclusive pH 7.2
A1B812	Pden_3588	Amidohydrolase	Cytoplasmic	Exclusive pH 7.2
A1B815	Pden_3591	Addiction module antitoxin, RelB/DinJ family	Unknown	Exclusive pH 7.2
A1B822	Pden_3599	Uncharacterized protein	Unknown	Exclusive pH 7.2
A1B843	Pden_3620	Adenine phosphoribosyltransferase (APRT) (EC 2.4.2.7)	Cytoplasmic	Exclusive pH 7.2
A1B857	Pden_3634	Uncharacterized protein	Unknown	Exclusive pH 7.2
A1B859	Pden_3636	OmpW family protein	Outer Membrane	Exclusive pH 7.2
A1B860	Pden_3637	Propionyl-CoA carboxylase (EC 6.4.1.3)	Cytoplasmic	Exclusive pH 7.2
A1B861	Pden_3638	Appr-1-p processing domain protein	Cytoplasmic	Exclusive pH 7.2
A1B864	Pden_3641	Hydroxymethylglutaryl-CoA lyase (EC 4.1.3.4)	Cytoplasmic	Exclusive pH 7.2
A1B865	Pden_3642	Methylglutaconyl-CoA hydratase (EC 4.2.1.18)	Cytoplasmic	Exclusive pH 7.2
A1B873	Pden_3650	Uncharacterized protein	Cytoplasmic	Exclusive pH 7.2
A1B879	Pden_3656	GumN family protein	Unknown	Exclusive pH 7.2
A1B884	Pden_3661	MaoC domain protein dehydratase	Cytoplasmic	Exclusive pH 7.2

A1B885	Pden_3662	Riboflavin biosynthesis protein (EC 2.7.1.26) (EC 2.7.7.2)	Cytoplasmic	Exclusive pH 7.2
A1B898	Pden_3675	Uncharacterized protein	Cytoplasmic	Exclusive pH 7.2
A1B8A9	Pden_3686	Uncharacterized protein	Unknown	Exclusive pH 7.2
A1B8B7	Pden_3694	CBS domain containing protein	Cytoplasmic/Membrane	Exclusive pH 7.2
A1B8C0	Pden_3697	Thiamine monophosphate synthase	Cytoplasmic	Exclusive pH 7.2
A1B8C2	Pden_3699	Heme A synthase (HAS) (EC 1.3.-.-) (Cytochrome aa3-controlling protein) (ctaA)	Cytoplasmic/Membrane	Exclusive pH 7.2
A1B8C4	Pden_3701	tRNA-2-methylthio-N(6)-dimethylallyladenosine synthase (EC 2.8.4.3) ((Dimethylallyl)adenosine tRNA methylthiotransferase MiaB) (tRNA-i(6)A37 methylthiotransferase)	Cytoplasmic	Exclusive pH 7.2
A1B8D0	Pden_3707	Uncharacterized protein	Unknown	Exclusive pH 7.2
A1B8D1	Pden_3708	Uncharacterized protein	Unknown	Exclusive pH 7.2
A1B8E4	Pden_3721	Uncharacterized protein	Cytoplasmic	Exclusive pH 7.2
A1B8G6	Pden_3743	Uncharacterized protein	Cytoplasmic	Exclusive pH 7.2
A1B8G9	Pden_3746	Putative transcriptional regulator, XRE family	Unknown	Exclusive pH 7.2
A1B8K7	Pden_3785	2-nitropropane dioxygenase, NPD	Unknown	Exclusive pH 7.2
A1B8L1	Pden_3789	N-(5'-phosphoribosyl)anthranilate isomerase (PRAI) (EC 5.3.1.24)	Unknown	Exclusive pH 7.2
A1B8L6	Pden_3794	Uncharacterized protein	Cytoplasmic	Exclusive pH 7.2
A1B8L9	Pden_3797	Transcriptional regulator, RpiR family	Cytoplasmic	Exclusive pH 7.2
A1B8N3	Pden_3811	Transcription-repair-coupling factor (TRCF) (EC 3.6.4.-)	Cytoplasmic	Exclusive pH 7.2
A1B8P6	Pden_3824	CoA-binding domain protein	Cytoplasmic	Exclusive pH 7.2
A1B8R1	Pden_3839	Uncharacterized protein	Cytoplasmic	Exclusive pH 7.2
A1B8S2	Pden_3850	Uncharacterized protein	Unknown	Exclusive pH 7.2
A1B8T4	Pden_3862	MscS Mechanosensitive ion channel	Cytoplasmic/Membrane	Exclusive pH 7.2
A1B8U3	Pden_3871	Uncharacterized protein	Cytoplasmic	Exclusive pH 7.2
A1B8U7	Pden_3875	Methylmalonyl-CoA mutase (EC 5.4.99.2)	Cytoplasmic	Exclusive pH 7.2
A1B8V2	Pden_3880	Chorismate mutase (EC 5.4.99.5)	Cytoplasmic	Exclusive pH 7.2
A1B8V9	Pden_3887	Chromosome partitioning protein	Cytoplasmic	Exclusive pH 7.2
A1B8W5	Pden_3893	Septum formation initiator	Cytoplasmic/Membrane	Exclusive pH 7.2
A1B8X2	Pden_3900	Uncharacterized protein	Cytoplasmic	Exclusive pH 7.2
A1B8X8	Pden_3906	3-hydroxyacyl-[acyl-carrier-protein] dehydratase FabZ (EC 4.2.1.59) ((3R)-hydroxymyristoyl-[acyl-carrier-protein] dehydratase) ((3R)-hydroxymyristoyl-ACP dehydrase) (Beta-hydroxyacyl-ACP dehydratase)	Cytoplasmic	Exclusive pH 7.2

A1B8Y0	Pden_3908	Uncharacterized protein	Unknown	Exclusive pH 7.2
A1B8Y7	Pden_3915	5-hydroxyisourate hydrolase (HIU hydrolase) (HIUHase) (EC 3.5.2.17)	Unknown	Exclusive pH 7.2
A1B8Z9	Pden_3927	Phosphoribosylglycinamide formyltransferase (EC 2.1.2.2) (5'-phosphoribosylglycinamide transformylase) (GAR transformylase) (GART)	Cytoplasmic	Exclusive pH 7.2
A1B900	Pden_3928	Amino acid/amide ABC transporter membrane protein 2, HAAT family	Cytoplasmic/Membrane	Exclusive pH 7.2
A1B901	Pden_3929	Amino acid/amide ABC transporter membrane protein 1, HAAT family	Cytoplasmic/Membrane	Exclusive pH 7.2
A1B909	Pden_3937	Transferase hexapeptide protein	Cytoplasmic	Exclusive pH 7.2
A1B910	Pden_3938	PAS/PAC sensor signal transduction histidine kinase (EC 2.7.13.3)	Cytoplasmic/Membrane	Exclusive pH 7.2
A1B915	Pden_3943	Pyrimidine 5'-nucleotidase	Cytoplasmic	Exclusive pH 7.2
A1B935	Pden_3963	Cysteine desulfurase (EC 2.8.1.7)	Unknown	Exclusive pH 7.2
A1B949	Pden_3977	Cytochrome c-type biogenesis protein CcmF	Cytoplasmic/Membrane	Exclusive pH 7.2
A1B951	Pden_3979	Enoyl-CoA hydratase (EC 4.2.1.17)	Cytoplasmic	Exclusive pH 7.2
A1B953	Pden_3981	Uncharacterized protein	Cytoplasmic/Membrane	Exclusive pH 7.2
A1B970	Pden_3998	Zinc metalloprotease (EC 3.4.24.-)	Cytoplasmic/Membrane	Exclusive pH 7.2
A1B972	Pden_4000	Outer membrane chaperone Skp (OmpH)	Unknown	Exclusive pH 7.2
A1B973	Pden_4001	Purine nucleosidase (EC 3.2.2.1)	Cytoplasmic	Exclusive pH 7.2
A1B986	Pden_4014	Dyp-type peroxidase family	Unknown	Exclusive pH 7.2
A1B987	Pden_4015	Linocin_M18 bacteriocin protein	Unknown	Exclusive pH 7.2
A1B990	Pden_4018	Amino acid/amide ABC transporter membrane protein 1, HAAT family	Cytoplasmic/Membrane	Exclusive pH 7.2
A1B993	Pden_4021	Amino acid/amide ABC transporter ATP-binding protein 2, HAAT family	Cytoplasmic/Membrane	Exclusive pH 7.2
A1B994	Pden_4022	Redoxin domain protein	Unknown	Exclusive pH 7.2
A1B9B4	Pden_4042	Uncharacterized protein	Cytoplasmic	Exclusive pH 7.2
A1B9B5	Pden_4043	ErfK/YbiS/YcfS/YnhG family protein	Unknown	Exclusive pH 7.2
A1B9C7	Pden_4055	Glutamate 5-kinase (EC 2.7.2.11) (Gamma-glutamyl kinase) (GK)	Cytoplasmic	Exclusive pH 7.2
A1B9D3	Pden_4061	Uncharacterized protein	Unknown	Exclusive pH 7.2
A1B9D5	Pden_4063	Cysteine--tRNA ligase (EC 6.1.1.16) (Cysteinyl-tRNA synthetase) (CysRS)	Cytoplasmic	Exclusive pH 7.2
A1B9E5	Pden_4073	DNA primase (EC 2.7.7.-)	Cytoplasmic	Exclusive pH 7.2
A1B9F3	Pden_4081	NifU-related protein involved in Fe-S cluster formation	Unknown	Exclusive pH 7.2
A1B9F5	Pden_4083	DNA ligase (EC 6.5.1.2) (Polydeoxyribonucleotide synthase [NAD(+)])	Cytoplasmic	Exclusive pH 7.2
A1B9H0	Pden_4098	tRNA-specific 2-thiouridylase MnmA (EC 2.8.1.13)	Cytoplasmic	Exclusive pH 7.2
A1B9H2	Pden_4100	Uncharacterized protein	Unknown	Exclusive pH 7.2

A1B9H6	Pden_4104	L-carnitine dehydratase/bile acid-inducible protein F	Cytoplasmic	Exclusive pH 7.2
A1B9I2	Pden_4110	HpcH/HpaI aldolase	Cytoplasmic	Exclusive pH 7.2
A1B9I3	Pden_4111	Uncharacterized protein	Cytoplasmic	Exclusive pH 7.2
A1B9I4	Pden_4112	L-carnitine dehydratase/bile acid-inducible protein F	Cytoplasmic	Exclusive pH 7.2
A1B9I6	Pden_4116	Uncharacterized protein	Cytoplasmic/Membrane	Exclusive pH 7.2
A1B9I7	Pden_4117	Pimeloyl-CoA biosynthesis protein BioC	Cytoplasmic	Exclusive pH 7.2
A1B9I8	Pden_4118	Transcriptional regulator, LysR family	Cytoplasmic	Exclusive pH 7.2
A1B9I9	Pden_4119	Fumarate hydratase class I (EC 4.2.1.2)	Cytoplasmic	Exclusive pH 7.2
A1B9J0	Pden_4120	TRAP dicarboxylate transporter, DctM subunit	Cytoplasmic/Membrane	Exclusive pH 7.2
A1B9J1	Pden_4121	Tripartite ATP-independent periplasmic transporter, DctQ component	Cytoplasmic/Membrane	Exclusive pH 7.2
A1B9J2	Pden_4122	TRAP dicarboxylate transporter, DctP subunit	Periplasmic	Exclusive pH 7.2
A1B9K9	Pden_4139	Putative ferric uptake regulator, Fur family	Cytoplasmic	Exclusive pH 7.2
A1B9P1	Pden_4171	Uncharacterized protein	Unknown	Exclusive pH 7.2
A1B9P4	Pden_4174	Transcriptional regulator, DeoR family	Unknown	Exclusive pH 7.2
A1B9P8	Pden_4178	Mannitol ABC transporter ATP-binding protein / sorbitol ABC transporter ATP-binding protein	Cytoplasmic/Membrane	Exclusive pH 7.2
A1B9Q7	Pden_4187	sn-glycerol-3-phosphate import ATP-binding protein UgpC (EC 3.6.3.20)	Cytoplasmic/Membrane	Exclusive pH 7.2
A1B9S3	Pden_4203	Periplasmic binding protein	Unknown	Exclusive pH 7.2
A1B9S8	Pden_4208	Acetate kinase (EC 2.7.2.1) (Acetokinase)	Cytoplasmic	Exclusive pH 7.2
A1B9T2	Pden_4212	Uncharacterized protein	Cytoplasmic/Membrane	Exclusive pH 7.2
A1B9T4	Pden_4214	FAD:protein FMN transferase (EC 2.7.1.180)	Unknown	Exclusive pH 7.2
A1B9V3	Pden_4233	Respiratory nitrate reductase gamma subunit (NarI)	Cytoplasmic/Membrane	Exclusive pH 7.2
A1B9V4	Pden_4234	Respiratory nitrate reductase chaperone NarJ	Cytoplasmic	Exclusive pH 7.2
A1B9V7	Pden_4237	Nitrite transporter	Cytoplasmic/Membrane	Exclusive pH 7.2
A1B9V8	Pden_4238	Putative transcriptional regulator, Crp/Fnr family	Cytoplasmic	Exclusive pH 7.2
A1B9W9	Pden_4249	Sodium:dicarboxylate symporter	Cytoplasmic/Membrane	Exclusive pH 7.2
A1B9X6	Pden_4256	Transcriptional regulator, LysR family	Cytoplasmic	Exclusive pH 7.2
A1BA14	Pden_4294	NmrA family protein	Unknown	Exclusive pH 7.2
A1BA15	Pden_4295	DNA helicase (EC 3.6.4.12)	Cytoplasmic	Exclusive pH 7.2
A1BA29	Pden_4309	Acetyl-CoA hydrolase (EC 3.1.2.1)	Cytoplasmic	Exclusive pH 7.2
A1BA50	Pden_4330	Phosphate ABC transporter substrate-binding protein, PhoT family	Cytoplasmic/Membrane	Exclusive pH 7.2
A1BAA5	Pden_4385	ABC transporter related protein	Cytoplasmic/Membrane	Exclusive pH 7.2

A1BAB7	Pden_4397	Glycolate oxidase iron-sulfur subunit	Cytoplasmic/Membrane	Exclusive pH 7.2
A1BAB8	Pden_4398	FAD linked oxidase domain protein	Cytoplasmic	Exclusive pH 7.2
A1BAC1	Pden_4401	Uncharacterized protein UPF0065	Unknown	Exclusive pH 7.2
A1BAC2	Pden_4402	Uncharacterized protein	Cytoplasmic/Membrane	Exclusive pH 7.2
A1BAC4	Pden_4404	Uncharacterized protein UPF0065	Unknown	Exclusive pH 7.2
A1BAD2	Pden_4412	Acyl-CoA dehydrogenase domain protein	Cytoplasmic	Exclusive pH 7.2
A1BAE6	Pden_4426	Glycogen synthase (EC 2.4.1.21) (Starch [bacterial glycogen] synthase)	Cytoplasmic	Exclusive pH 7.2
A1BAE7	Pden_4427	Glucose-1-phosphate adenylyltransferase (EC 2.7.7.27) (ADP-glucose pyrophosphorylase) (ADPGlc PPase) (ADP-glucose synthase)	Cytoplasmic	Exclusive pH 7.2
A1BAF0	Pden_4430	Nicotinate-nucleotide pyrophosphorylase (Carboxylating) (EC 2.4.2.19)	Cytoplasmic	Exclusive pH 7.2
A1BAF1	Pden_4431	L-aspartate oxidase (EC 1.4.3.16)	Cytoplasmic	Exclusive pH 7.2
A1BAH3	Pden_4453	Major facilitator superfamily MFS_1	Cytoplasmic/Membrane	Exclusive pH 7.2
A1BAH5	Pden_4455	Response regulator receiver and ANTAR domain protein	Cytoplasmic	Exclusive pH 7.2
A1BAI0	Pden_4460	ADP-dependent (S)-NAD(P)H-hydrate dehydratase (EC 4.2.1.136) (EC 5.1.99.6) (ADP-dependent NAD(P)HX dehydratase) (NAD(P)H-hydrate epimerase) (NAD(P)HX epimerase)	Cytoplasmic	Exclusive pH 7.2
A1BAI6	Pden_4466	Antifreeze protein, type I	Unknown	Exclusive pH 7.2
A1BAI7	Pden_4467	Dihydroorotase (EC 3.5.2.3)	Cytoplasmic	Exclusive pH 7.2
A1BAK0	Pden_4480	Acyl-CoA dehydrogenase domain protein	Cytoplasmic	Exclusive pH 7.2
A1BAK1	Pden_4481	Enoyl-CoA hydratase/isomerase	Cytoplasmic	Exclusive pH 7.2
A1BAK4	Pden_4484	DNA repair protein RecN (Recombination protein N)	Cytoplasmic	Exclusive pH 7.2
A1BAL1	Pden_4491	UDP-N-acetylenolpyruvoylglucosamine reductase (EC 1.3.1.98) (UDP-N-acetylmuramate dehydrogenase)	Cytoplasmic	Exclusive pH 7.2
A1BAL8	Pden_4498	Uncharacterized protein	Cytoplasmic	Exclusive pH 7.2
A1BAM2	Pden_4502	ABC polyamine transporter, periplasmic substrate-binding protein	Periplasmic	Exclusive pH 7.2
A1BAM9	Pden_4509	Uncharacterized protein	Cytoplasmic	Exclusive pH 7.2
A1BAN1	Pden_4511	Ubiquinone/menaquinone biosynthesis C-methyltransferase UbiE (EC 2.1.1.163) (EC 2.1.1.201) (2-methoxy-6-polyprenyl-1,4-benzoquinol methylase) (Demethylmenaquinone methyltransferase)	Cytoplasmic	Exclusive pH 7.2
A1BAN5	Pden_4515	3-demethylubiquinone-9 3-methyltransferase	Unknown	Exclusive pH 7.2
A1BAN9	Pden_4519	Activator of Hsp90 ATPase 1 family protein	Cytoplasmic	Exclusive pH 7.2
A1BAR5	Pden_4546	TRAP dicarboxylate transporter-DctP subunit	Unknown	Exclusive pH 7.2
A1BAR6	Pden_4547	L-glutamine synthetase (EC 6.3.1.2)	Cytoplasmic	Exclusive pH 7.2

A1BAR8	Pden_4549	Iron-containing alcohol dehydrogenase	Cytoplasmic	Exclusive pH 7.2
A1BAS1	Pden_4552	Na+/solute symporter	Cytoplasmic/Membrane	Exclusive pH 7.2
A1BAU3	Pden_4574	Aldo/keto reductase	Cytoplasmic	Exclusive pH 7.2
A1BAX2	Pden_4603	Putative ABC transporter binding protein component	Periplasmic	Exclusive pH 7.2
A1BAY5	Pden_4616	Dihydropyrimidinase (EC 3.5.2.2)	Cytoplasmic	Exclusive pH 7.2
A1BB09	Pden_4642	Uncharacterized protein	Unknown	Exclusive pH 7.2
A1BB12	Pden_4645	Transcriptional regulator, TetR family	Cytoplasmic	Exclusive pH 7.2
A1BB21	Pden_4654	HAD superfamily (Subfamily IIIB) phosphatase, TIGR01672 (EC 3.1.3.2)	Unknown	Exclusive pH 7.2
A1BB44	Pden_4677	PKHD-type hydroxylase Pden_4677 (EC 1.14.11.-)	Cytoplasmic	Exclusive pH 7.2
A1BB59	Pden_4692	Phosphatidylserine decarboxylase proenzyme (EC 4.1.1.65) [Cleaved into: Phosphatidylserine decarboxylase beta chain; Phosphatidylserine decarboxylase alpha chain]	Cytoplasmic	Exclusive pH 7.2
A1BB61	Pden_4694	FAD-dependent pyridine nucleotide-disulfide oxidoreductase	Periplasmic	Exclusive pH 7.2
A1BB68	Pden_4701	Transcriptional regulator, ArsR family	Unknown	Exclusive pH 7.2
A1BB73	Pden_4706	Beta-lactamase domain protein	Cytoplasmic	Exclusive pH 7.2
A1BB77	Pden_4710	Glucose-methanol-choline oxidoreductase	Cytoplasmic	Exclusive pH 7.2
A1BB94	Pden_4727	Glucose sorbosone dehydrogenase	Unknown	Exclusive pH 7.2
A1BBC5	Pden_4758	Branched-chain alpha-keto acid dehydrogenase E1 component (EC 1.2.4.4)	Cytoplasmic	Exclusive pH 7.2
A1BBC6	Pden_4759	Dihydrolipoamide acetyltransferase component of pyruvate dehydrogenase complex (EC 2.3.1.-)	Cytoplasmic	Exclusive pH 7.2
A1BBC7	Pden_4760	Dihydrolipoyl dehydrogenase (EC 1.8.1.4)	Cytoplasmic	Exclusive pH 7.2
A1BBD8	Pden_4771	Monosaccharide ABC transporter substrate-binding protein, CUT2 family	Periplasmic	Exclusive pH 7.2
A1BBF9	Pden_4792	3-hydroxyacyl-CoA dehydrogenase (EC 1.1.1.35)	Cytoplasmic	Exclusive pH 7.2
A1BBG4	Pden_4797	Amino acid/amide ABC transporter substrate-binding protein, HAAT family	Periplasmic	Exclusive pH 7.2
A1BBG8	Pden_4801	Phenylacetate-coenzyme A ligase (EC 6.2.1.30) (Phenylacetyl-CoA ligase)	Cytoplasmic	Exclusive pH 7.2
A1BBI7	Pden_4820	AMP-dependent synthetase and ligase	Cytoplasmic	Exclusive pH 7.2
A1BBI9	Pden_4822	Short-chain dehydrogenase/reductase SDR	Cytoplasmic	Exclusive pH 7.2
A1BBJ1	Pden_4824	Phosphoglucomutase/phosphomannomutase alpha/beta/alpha domain I	Cytoplasmic	Exclusive pH 7.2
A1BBL2	Pden_4846	Periplasmic binding protein/LacI transcriptional regulator	Cytoplasmic	Exclusive pH 7.2
A1BBN9	Pden_4873	Putative spermidine/putrescine transport system substrate-binding protein	Periplasmic	Exclusive pH 7.2
A1BBQ4	Pden_4888	Substrate-binding region of ABC-type glycine betaine transport system	Cytoplasmic/Membrane	Exclusive pH 7.2
A1BBZ8	Pden_4982	Short-chain dehydrogenase/reductase SDR	Unknown	Exclusive pH 7.2

A1BBZ9	Pden_4983	Alpha/beta hydrolase fold protein	Cytoplasmic	Exclusive pH 7.2
A1BC00	Pden_4984	Transketolase, central region	Cytoplasmic	Exclusive pH 7.2
A1BC11	Pden_4995	Transcriptional regulator, GntR family	Cytoplasmic	Exclusive pH 7.2
A1BC41	Pden_5025	Glycerol-3-phosphate cytidylyltransferase (EC 2.7.7.39)	Cytoplasmic	Exclusive pH 7.2
A1BC45	Pden_5029	Uncharacterized protein	Cytoplasmic	Exclusive pH 7.2
A1BC46	Pden_5030	Uncharacterized protein	Cytoplasmic	Exclusive pH 7.2
A1BC83	Pden_5067	Transcriptional regulator, GntR family	Cytoplasmic	Exclusive pH 7.2
A1BCB8	Pden_5102	ATPase, P-type (Transporting), HAD superfamily, subfamily IC	Cytoplasmic/Membrane	Exclusive pH 7.2
A1BCB9	Pden_5103	Poly-beta-hydroxybutyrate polymerase domain protein	Cytoplasmic	Exclusive pH 7.2
A1BCC6	Pden_5110	Putative signal-transduction protein with CBS domains	Cytoplasmic	Exclusive pH 7.2
A1BCC8	Pden_5112	Integral membrane sensor signal transduction histidine kinase	Cytoplasmic/Membrane	Exclusive pH 7.2
A1BCC9	Pden_5113	Response regulator receiver protein	Cytoplasmic	Exclusive pH 7.2
A1BCD0	Pden_5114	DNA polymerase III, epsilon subunit (EC 2.7.7.7)	Unknown	Exclusive pH 7.2
A1BCD2	Pden_5116	Agmatinase (EC 3.5.3.11)	Cytoplasmic	Exclusive pH 7.2
A1BCD5	Pden_5119	NADPH-dependent FMN reductase	Unknown	Exclusive pH 7.2
A1BCF0	Pden_5134	Uncharacterized protein	Unknown	Exclusive pH 7.2
Q51699	Pden_2486	Protein NirI	Cytoplasmic/Membrane	Exclusive pH 7.2
A1AY04	Pden_0031	Uncharacterized protein	Unknown	Exclusive pH 7.2
A1AY05	Pden_0032	Molybdenum cofactor guanylyltransferase (MoCo guanylyltransferase) (EC 2.7.7.77) (GTP:molybdopterin guanylyltransferase) (Mo-MPT guanylyltransferase) (Molybdopterin guanylyltransferase) (Molybdopterin-guanine dinucleotide synthase) (MGD synthase)	Cytoplasmic	Exclusive pH 7.2
A1AY06	Pden_0033	Binding-protein-dependent transport systems inner membrane component	Cytoplasmic/Membrane	Exclusive pH 7.2
A1AY84	Pden_0111	Uncharacterized protein	Cytoplasmic/Membrane	Exclusive pH 7.2
A1AYI0	Pden_0210	Uncharacterized protein	Unknown	Exclusive pH 7.2
A1AYS1	Pden_0301	L-seryl-tRNA(Sec) selenium transferase (EC 2.9.1.1) (Selenocysteine synthase) (Sec synthase) (Selenocysteinyl-tRNA(Sec) synthase)	Cytoplasmic	Exclusive pH 7.2
A1AYT3	Pden_0313	Uncharacterized protein	Unknown	Exclusive pH 7.2
A1AZ18	Pden_0398	Exodeoxyribonuclease 7 small subunit (EC 3.1.11.6) (Exodeoxyribonuclease VII small subunit) (Exonuclease VII small subunit)	Cytoplasmic	Exclusive pH 7.2
A1AZ58	Pden_0438	Phage SPO1 DNA polymerase-related protein	Unknown	Exclusive pH 7.2
A1AZC1	Pden_0503	Ribose ABC transporter ATP-binding protein / fructose ABC transporter ATP-binding protein / mannose ABC transporter ATP-binding protein	Cytoplasmic/Membrane	Exclusive pH 7.2

A1AZD5	Pden_0517	Uncharacterized protein	Unknown	Exclusive pH 7.2
A1AZE3	Pden_0525	Nucleoside ABC transporter membrane protein	Cytoplasmic/Membrane	Exclusive pH 7.2
A1AZG2	Pden_0544	Dihydropteroate synthase (DHPS) (EC 2.5.1.15) (Dihydropteroate pyrophosphorylase)	Cytoplasmic	Exclusive pH 7.2
A1AZH1	Pden_0553	Uncharacterized protein	Unknown	Exclusive pH 7.2
A1AZJ7	Pden_0579	Ribosomal RNA large subunit methyltransferase J (EC 2.1.1.266) (23S rRNA (adenine(2030)-N6)-methyltransferase) (23S rRNA m6A2030 methyltransferase)	Cytoplasmic	Exclusive pH 7.2
A1AZP6	Pden_0628	Uncharacterized protein	Cytoplasmic/Membrane	Exclusive pH 7.2
A1AZQ7	Pden_0639	Uncharacterized protein	Cytoplasmic	Exclusive pH 7.2
A1AZR2	Pden_0644	Uncharacterized protein	Cytoplasmic/Membrane	Exclusive pH 7.2
A1AZV7	Pden_0689	tRNA(Ile)-lysidine synthase (EC 6.3.4.19) (tRNA(Ile)-2-lysyl-cytidine synthase) (tRNA(Ile)-lysidine synthetase)	Cytoplasmic	Exclusive pH 7.2
A1AZV9	Pden_0691	Chorismate mutase (EC 5.4.99.5)	Unknown	Exclusive pH 7.2
A1AZW6	Pden_0698	Multisubunit potassium/proton antiporter, PhaC subunit	Cytoplasmic/Membrane	Exclusive pH 7.2
A1B056	Pden_0788	Recombination protein MgsA	Cytoplasmic	Exclusive pH 7.2
A1B0B9	Pden_0851	Nicotinamidase (EC 3.5.1.19)	Cytoplasmic	Exclusive pH 7.2
A1B0C6	Pden_0858	Pyridoxine/pyridoxamine 5'-phosphate oxidase (EC 1.4.3.5) (PNP/PMP oxidase) (PNPOx) (Pyridoxal 5'-phosphate synthase)	Cytoplasmic	Exclusive pH 7.2
A1B0E5	Pden_0878	MOSC domain containing protein	Cytoplasmic	Exclusive pH 7.2
A1B0I3	Pden_0916	Transcriptional regulator, TraR/DksA family	Cytoplasmic	Exclusive pH 7.2
A1B0I8	Pden_0921	NAD kinase (EC 2.7.1.23) (ATP-dependent NAD kinase)	Cytoplasmic	Exclusive pH 7.2
A1B0L4	Pden_0947	Glycine betaine/L-proline ABC transporter, ATPase subunit	Cytoplasmic/Membrane	Exclusive pH 7.2
A1B0P0	Pden_0973	Uncharacterized protein	Unknown	Exclusive pH 7.2
A1B1A4	Pden_1193	Glycine betaine/L-proline ABC transporter, ATPase subunit	Cytoplasmic/Membrane	Exclusive pH 7.2
A1B1C6	Pden_1215	Activator of Hsp90 ATPase 1 family protein	Unknown	Exclusive pH 7.2
A1B1G0	Pden_1249	Binding-protein-dependent transport systems inner membrane component	Cytoplasmic/Membrane	Exclusive pH 7.2
A1B1J1	Pden_1280	Uncharacterized protein	Unknown	Exclusive pH 7.2
A1B1L1	Pden_1300	Uncharacterized protein	Cytoplasmic/Membrane	Exclusive pH 7.2
A1B1M9	Pden_1318	Probable nicotinate-nucleotide adenylyltransferase (EC 2.7.7.18) (Deamido-NAD(+) diphosphorylase) (Deamido-NAD(+) pyrophosphorylase) (Nicotinate mononucleotide adenylyltransferase) (NaMN adenylyltransferase)	Unknown	Exclusive pH 7.2
A1B1N8	Pden_1327	Binding-protein-dependent transport systems inner membrane component	Cytoplasmic/Membrane	Exclusive pH 7.2

A1B1X5	Pden_1418	MazG family protein	Cytoplasmic	Exclusive pH 7.2
A1B2A5	Pden_1549	Uncharacterized protein	Unknown	Exclusive pH 7.2
A1B2K7	Pden_1654	Lysine--tRNA ligase (EC 6.1.1.6)	Cytoplasmic	Exclusive pH 7.2
A1B2N3	Pden_1680	Oxidoreductase domain protein	Unknown	Exclusive pH 7.2
A1B2N5	Pden_1682	Monosaccharide ABC transporter membrane protein, CUT2 family	Cytoplasmic/Membrane	Exclusive pH 7.2
A1B2R8	Pden_1715	Amino acid/amide ABC transporter ATP-binding protein 2, HAAT family	Cytoplasmic	Exclusive pH 7.2
A1B2X4	Pden_1771	Integral membrane sensor signal transduction histidine kinase	Cytoplasmic/Membrane	Exclusive pH 7.2
A1B2Y2	Pden_1779	Uncharacterized conserved protein UCP032025	Unknown	Exclusive pH 7.2
A1B2Y9	Pden_1786	Response regulator receiver protein	Cytoplasmic	Exclusive pH 7.2
A1B2Z5	Pden_1792	Ribonuclease	Cytoplasmic	Exclusive pH 7.2
A1B316	Pden_1813	Pseudouridine-5'-phosphate glycosidase (PsiMP glycosidase) (EC 4.2.1.70)	Unknown	Exclusive pH 7.2
A1B349	Pden_1846	Cbb3-type cytochrome oxidase component	Unknown	Exclusive pH 7.2
A1B353	Pden_1850	Putative transcriptional regulator, Crp/Fnr family	Cytoplasmic/Membrane	Exclusive pH 7.2
A1B3C3	Pden_1923	ATP phosphoribosyltransferase (EC 2.4.2.17)	Cytoplasmic	Exclusive pH 7.2
A1B3H9	Pden_1981	GCN5-related N-acetyltransferase	Periplasmic	Exclusive pH 7.2
A1B3I3	Pden_1985	Putative pterin-4-alpha-carbinolamine dehydratase (PHS) (EC 4.2.1.96) (4-alpha-hydroxy-tetrahydropterin dehydratase) (Pterin carbinolamine dehydratase) (PCD)	Cytoplasmic	Exclusive pH 7.2
A1B3L2	Pden_2014	ABC transporter related protein	Cytoplasmic/Membrane	Exclusive pH 7.2
A1B3T5	Pden_2087	Uncharacterized protein	Cytoplasmic	Exclusive pH 7.2
A1B3W8	Pden_2120	dTDP-4-dehydrorhamnose reductase (EC 1.1.1.133)	Unknown	Exclusive pH 7.2
A1B3X5	Pden_2127	ABC transporter related protein	Cytoplasmic	Exclusive pH 7.2
A1B461	Pden_2213	Prolipoprotein diacylglyceryl transferase (EC 2.4.99.-)	Cytoplasmic/Membrane	Exclusive pH 7.2
A1B4B4	Pden_2266	Transcriptional regulator, AsnC family	Cytoplasmic	Exclusive pH 7.2
A1B4G6	Pden_2319	Methylenetetrahydrofolate reductase (EC 1.5.1.20)	Cytoplasmic	Exclusive pH 7.2
A1B4I2	Pden_2335	FAD dependent oxidoreductase	Unknown	Exclusive pH 7.2
A1B4I7	Pden_2340	Molybdopterin synthase subunit MoaE	Cytoplasmic	Exclusive pH 7.2
A1B4J8	Pden_2352	Histidine kinase	Cytoplasmic/Membrane	Exclusive pH 7.2
A1B4L3	Pden_2367	Alcohol dehydrogenase GroES domain protein	Cytoplasmic	Exclusive pH 7.2
A1B4L6	Pden_2370	Peptidase S16, lon domain protein	Cytoplasmic	Exclusive pH 7.2
A1B4M1	Pden_2375	GTP cyclohydrolase-2 (EC 3.5.4.25) (GTP cyclohydrolase II)	Cytoplasmic	Exclusive pH 7.2
A1B4M4	Pden_2378	Putative RNA methylase	Cytoplasmic	Exclusive pH 7.2

A1B4Q6	Pden_2412	Type I secretion membrane fusion protein, HlyD family	Cytoplasmic/Membrane	Exclusive pH 7.2
A1B4T6	Pden_2443	Uncharacterized conserved protein UCP028301	Cytoplasmic	Exclusive pH 7.2
A1B4Y5	Pden_2492	Transcriptional regulator, AsnC family	Unknown	Exclusive pH 7.2
A1B500	Pden_2507	Peptidase M23B	Unknown	Exclusive pH 7.2
A1B504	Pden_2511	Uncharacterized protein	Cytoplasmic	Exclusive pH 7.2
A1B523	Pden_2530	Uncharacterized protein	Unknown	Exclusive pH 7.2
A1B535	Pden_2542	Precorrin-6A synthase (Deacetylating) (EC 2.1.1.152)	Cytoplasmic	Exclusive pH 7.2
A1B546	Pden_2553	Glycerol-3-phosphate acyltransferase (Acyl-PO4 G3P acyltransferase) (Acyl-phosphate--glycerol-3-phosphate acyltransferase) (G3P acyltransferase) (GPAT) (EC 2.3.1.n3) (Lysophosphatidic acid synthase) (LPA synthase)	Cytoplasmic/Membrane	Exclusive pH 7.2
A1B550	Pden_2557	Uncharacterized protein	Cytoplasmic	Exclusive pH 7.2
A1B557	Pden_2564	Amino acid adenylation domain	Cytoplasmic	Exclusive pH 7.2
A1B564	Pden_2571	Three-deoxy-D-manno-octulosonic-acid transferase domain protein	Unknown	Exclusive pH 7.2
A1B581	Pden_2588	Transcriptional regulator, MarR family	Cytoplasmic	Exclusive pH 7.2
A1B584	Pden_2591	Ribosome maturation factor RimP	Cytoplasmic	Exclusive pH 7.2
A1B589	Pden_2596	Mutator MutT protein	Unknown	Exclusive pH 7.2
A1B5B0	Pden_2617	Iron-containing alcohol dehydrogenase	Cytoplasmic	Exclusive pH 7.2
A1B5E9	Pden_2656	ATP-dependent DNA helicase RecQ (EC 3.6.1.-)	Cytoplasmic	Exclusive pH 7.2
A1B5N3	Pden_2743	Elongation factor P (EF-P)	Cytoplasmic	Exclusive pH 7.2
A1B5P8	Pden_2758	Uncharacterized protein	Cytoplasmic	Exclusive pH 7.2
A1B5Q8	Pden_2768	HI0933 family protein	Cytoplasmic	Exclusive pH 7.2
A1B5R7	Pden_2777	5'-methylthioadenosine/S-adenosylhomocysteine nucleosidase	Cytoplasmic	Exclusive pH 7.2
A1B5U7	Pden_2807	Uncharacterized protein	Cytoplasmic	Exclusive pH 7.2
A1B5V3	Pden_2813	Shikimate dehydrogenase (NADP(+)) (SDH) (EC 1.1.1.25)	Cytoplasmic	Exclusive pH 7.2
A1B5Y9	Pden_2849	PTS system fructose subfamily IIA component	Unknown	Exclusive pH 7.2
A1B604	Pden_2864	Thioesterase superfamily protein	Unknown	Exclusive pH 7.2
A1B624	Pden_2884	Dihydroorotate dehydrogenase (quinone) (EC 1.3.5.2)	Cytoplasmic/Membrane	Exclusive pH 7.2
A1B625	Pden_2885	Serine O-acetyltransferase (EC 2.3.1.30)	Cytoplasmic	Exclusive pH 7.2
A1B659	Pden_2919	Adenosylmethionine-8-amino-7-oxononanoate aminotransferase apoenzyme (EC 2.6.1.62)	Unknown	Exclusive pH 7.2
A1B669	Pden_2929	Transcriptional regulator, GntR family	Cytoplasmic	Exclusive pH 7.2
A1B671	Pden_2931	Gluconate transporter	Cytoplasmic/Membrane	Exclusive pH 7.2

A1B6A8	Pden_2968	Uncharacterized protein	Cytoplasmic	Exclusive pH 7.2
A1B6D2	Pden_2993	Pyrrolo-quinoline quinone	Periplasmic	Exclusive pH 7.2
A1B6G3	Pden_3024	Transcriptional regulator, BadM/Rrf2 family	Cytoplasmic	Exclusive pH 7.2
A1B6H7	Pden_3038	Choline/carnitine/betaine transporter	Cytoplasmic/Membrane	Exclusive pH 7.2
A1B722	Pden_3235	Uncharacterized protein	Unknown	Exclusive pH 7.2
A1B735	Pden_3248	Regulatory protein, IclR	Unknown	Exclusive pH 7.2
A1B7G6	Pden_3387	TRAP dicarboxylate transporter, DctP subunit	Periplasmic	Exclusive pH 7.2
A1B7P5	Pden_3468	Putative transcriptional regulator, Crp/Fnr family	Cytoplasmic	Exclusive pH 7.2
A1B7Q6	Pden_3480	TRAP dicarboxylate transporter, DctP subunit	Periplasmic	Exclusive pH 7.2
A1B7W1	Pden_3535	Glutathione S-transferase, N-terminal domain	Cytoplasmic	Exclusive pH 7.2
A1B7Y0	Pden_3554	Uncharacterized protein	Cytoplasmic	Exclusive pH 7.2
A1B848	Pden_3625	AAA ATPase	Cytoplasmic	Exclusive pH 7.2
A1B8A0	Pden_3677	Transcriptional regulator, GntR family	Cytoplasmic	Exclusive pH 7.2
A1B8A2	Pden_3679	Uncharacterized protein	Cytoplasmic	Exclusive pH 7.2
A1B8G8	Pden_3745	Uncharacterized protein	Unknown	Exclusive pH 7.2
A1B8P3	Pden_3821	Alpha/beta hydrolase fold protein	Cytoplasmic	Exclusive pH 7.2
A1B8V1	Pden_3879	GCN5-related N-acetyltransferase	Unknown	Exclusive pH 7.2
A1B8V4	Pden_3882	Ribosome maturation factor RimM	Cytoplasmic	Exclusive pH 7.2
A1B8W1	Pden_3889	Acriflavin resistance protein	Cytoplasmic/Membrane	Exclusive pH 7.2
A1B8Y6	Pden_3914	Uncharacterized protein	Cytoplasmic/Membrane	Exclusive pH 7.2
A1B8Z1	Pden_3919	D-3-hydroxyaspartate aldolase (EC 4.1.3.-)	Cytoplasmic	Exclusive pH 7.2
A1B8Z2	Pden_3920	Pyridoxal-5'-phosphate-dependent enzyme, beta subunit	Cytoplasmic	Exclusive pH 7.2
A1B905	Pden_3933	Transcriptional regulator, AraC family with amidase-like domain	Cytoplasmic	Exclusive pH 7.2
A1B908	Pden_3936	Guanylate kinase (EC 2.7.4.8) (GMP kinase)	Cytoplasmic	Exclusive pH 7.2
A1B962	Pden_3990	Ribosomal RNA large subunit methyltransferase E (EC 2.1.1.166) (23S rRNA Um2552 methyltransferase) (rRNA (uridine-2'-O-)-methyltransferase)	Cytoplasmic	Exclusive pH 7.2
A1B998	Pden_4026	Uncharacterized protein	Cytoplasmic	Exclusive pH 7.2
A1B9I5	Pden_4115	Glyoxalase/bleomycin resistance protein/dioxygenase	Cytoplasmic	Exclusive pH 7.2
A1B9K2	Pden_4132	L-serine ammonia-lyase (EC 4.3.1.17)	Cytoplasmic	Exclusive pH 7.2
A1B9T6	Pden_4216	Nitrous oxide maturation protein NosY	Cytoplasmic/Membrane	Exclusive pH 7.2
A1B9X7	Pden_4257	Molybdopterin dehydrogenase, FAD-binding protein	Cytoplasmic	Exclusive pH 7.2

A1B9Y0	Pden_4260	Guanine deaminase (EC 3.5.4.3)	Cytoplasmic	Exclusive pH 7.2
A1B9Y5	Pden_4265	Transcriptional regulator, GntR family	Cytoplasmic	Exclusive pH 7.2
A1B9Z2	Pden_4272	Amino acid/amide ABC transporter substrate-binding protein, HAAT family	Unknown	Exclusive pH 7.2
A1BA24	Pden_4304	Iron-sulfur cluster assembly accessory protein	Cytoplasmic	Exclusive pH 7.2
A1BA32	Pden_4312	FAD linked oxidase domain protein	Cytoplasmic	Exclusive pH 7.2
A1BA36	Pden_4316	SURF1-like protein	Unknown	Exclusive pH 7.2
A1BA93	Pden_4373	TonB-dependent siderophore receptor	Outer Membrane	Exclusive pH 7.2
A1BAA9	Pden_4389	Carbohydrate ABC transporter membrane protein 2, CUT1 family	Cytoplasmic/Membrane	Exclusive pH 7.2
A1BAB9	Pden_4399	FAD linked oxidase domain protein	Cytoplasmic	Exclusive pH 7.2
A1BAD5	Pden_4415	Transcriptional regulator, XRE family	Cytoplasmic	Exclusive pH 7.2
A1BAE1	Pden_4421	Phosphonate metabolism protein PhnM	Cytoplasmic	Exclusive pH 7.2
A1BAF4	Pden_4434	Putative aminotransferase, class IV	Unknown	Exclusive pH 7.2
A1BAK6	Pden_4486	UDP-3-O-acyl-N-acetylglucosamine deacetylase (UDP-3-O-acyl-GlcNAc deacetylase) (EC 3.5.1.108) (UDP-3-O-[R-3-hydroxymyristoyl]-N-acetylglucosamine deacetylase)	Cytoplasmic	Exclusive pH 7.2
A1BAS2	Pden_4553	Cyclic nucleotide-binding protein	Cytoplasmic	Exclusive pH 7.2
A1BAZ9	Pden_4630	Transcriptional regulator, TetR family	Unknown	Exclusive pH 7.2
A1BB76	Pden_4709	Uncharacterized protein	Unknown	Exclusive pH 7.2
A1BBC4	Pden_4757	Branched-chain alpha-keto acid dehydrogenase E1 component (EC 1.2.4.4)	Cytoplasmic	Exclusive pH 7.2
A1BBH8	Pden_4811	Beta-ketoadipyl CoA thiolase (EC 2.3.1.9)	Cytoplasmic	Exclusive pH 7.2
A1BBN0	Pden_4864	Amino acid ABC transporter substrate-binding protein, PAAT family	Periplasmic	Exclusive pH 7.2
A1BBT1	Pden_4915	Transcriptional regulator, DeoR family	Cytoplasmic	Exclusive pH 7.2
A1BBW5	Pden_4949	Uncharacterized protein	Cytoplasmic	Exclusive pH 7.2
A1BC01	Pden_4985	Pyruvate dehydrogenase (Acetyl-transferring) (EC 1.2.4.1)	Cytoplasmic	Exclusive pH 7.2
A1BC12	Pden_4996	CoA-binding domain protein	Cytoplasmic	Exclusive pH 7.2
A1BC53	Pden_5037	ABC transporter related protein	Cytoplasmic	Exclusive pH 7.2
A1BC63	Pden_5047	Extracellular solute-binding protein, family 1	Periplasmic	Exclusive pH 7.2
A1BC82	Pden_5066	TRAP dicarboxylate transporter, DctP subunit	Periplasmic	Exclusive pH 7.2
P52220	Pden_1411	Heme exporter protein C (Cytochrome c-type biogenesis protein CcmC)	Cytoplasmic/Membrane	Exclusive pH 7.2
Q51703	Pden_2491	Protein NirD	Cytoplasmic	Exclusive pH 7.2

[1]Protein annotated from UniProt (UP000000361). [2]Genes annotated from GeneBank (T00440). [3]Subcellular location according with PSOTb v3.0.2.[4]Fold change calculated as the ratio protein expression at pH 7.2/pH 6.5 (positive values indicate proteins over-

represented at pH 7.2 and negative values correspond to proteins over-represented at pH 6.5). Proteins showing an absolute value of fold change >100 were considered exclusive at the indicated pH.

Table S2. Differential analysis of the *P. denitrificans* proteome by LC-MS/MS at pH 7.2 *versus* pH 7.0 (pH 7.2 was used as reference).

Protein ID[1]	Gene ID[2]	Name	Location[3]	FC[4]
A1AY25	Pden_0052	Amino acid/amide ABC transporter substrate-binding protein, HAAT family	Unknown	1239.26
A1BAC1	Pden_4401	Uncharacterized protein UPF0065	Unknown	81.81
A1B646	Pden_2906	Acyl-CoA dehydrogenase domain protein	Cytoplasmic	56.71
A1B862	Pden_3639	3-methylcrotonoyl-CoA carboxylase, alpha subunit (EC 6.4.1.4)	Cytoplasmic	42.75
A1BBI4	Pden_4817	TRAP dicarboxylate transporter, DctP subunit	Periplasmic	37.41
A1B2S0	Pden_1717	Amino acid/amide ABC transporter substrate-binding protein, HAAT family	Unknown	26.91
A1B2N7	Pden_1684	Monosaccharide ABC transporter substrate-binding protein, CUT2 family	Periplasmic	24.82
A1B856	Pden_3633	Isovaleryl-CoA dehydrogenase (EC 1.3.8.4)	Cytoplasmic	20.62
A1B647	Pden_2907	Acetyl-CoA acetyltransferase (EC 2.3.1.9)	Cytoplasmic	19.27
A1B9T1	Pden_4211	SSS sodium solute transporter superfamily	Cytoplasmic/Membrane	17.90
A1AZC3	Pden_0505	Mannose-binding protein / fructose-binding protein / ribose-binding protein	Periplasmic	16.09
A1B078	Pden_0810	Extracellular solute-binding protein, family 5	Periplasmic	15.72
A1B9T3	Pden_4213	Acetyl-coenzyme A synthetase (AcCoA synthetase) (Acs) (EC 6.2.1.1) (Acetate--CoA ligase) (Acyl-activating enzyme)	Cytoplasmic	13.12
A1BAR9	Pden_4550	Acetyl-coenzyme A synthetase (AcCoA synthetase) (Acs) (EC 6.2.1.1) (Acetate--CoA ligase) (Acyl-activating enzyme)	Cytoplasmic	13.12
A1BAG7	Pden_4447	Extracellular solute-binding protein, family 1	Periplasmic	11.53
A1B8B1	Pden_3688	Propionyl-CoA carboxylase carboxyltransferase subunit	Cytoplasmic	11.42
A1AZE6	Pden_0528	Nucleoside-binding protein	Unknown	11.07
A1B8A7	Pden_3684	Biotin carboxyl carrier protein / biotin carboxylase (EC 6.3.4.14)	Cytoplasmic	10.12
A1B4W5	Pden_2472	Carbohydrate ABC transporter substrate-binding protein, CUT1 family	Periplasmic	10.08
A1B3K3	Pden_2005	AMP-dependent synthetase and ligase	Cytoplasmic	9.31
A1B2M4	Pden_1671	Extracellular solute-binding protein, family 5	Periplasmic	9.24
A1B5F6	Pden_2663	Acetyl-CoA acetyltransferase (EC 2.3.1.9)	Cytoplasmic	8.67
A1B860	Pden_3637	Propionyl-CoA carboxylase (EC 6.4.1.3)	Cytoplasmic	8.23
A1B3B6	Pden_1916	Peptidyl-prolyl cis-trans isomerase (PPIase) (EC 5.2.1.8)	Unknown	8.05
A1B4D2	Pden_2285	Substrate-binding region of ABC-type glycine betaine transport system	Periplasmic	7.93
A1B2T8	Pden_1735	Dyp-type peroxidase family	Periplasmic	7.76
A1B097	Pden_0829	Extracellular solute-binding protein, family 1	Unknown	6.82
A1B2Y3	Pden_1780	Efflux transporter, RND family, MFP subunit	Cytoplasmic/Membrane	6.82
A1B9S9	Pden_4209	Phosphate butyryltransferase (EC 2.3.1.19)	Cytoplasmic	6.76
A1B067	Pden_0799	Citrate (Pro-3S)-lyase (EC 4.1.3.6)	Cytoplasmic	6.63
A1B8A4	Pden_3681	Methylmalonyl-CoA mutase (EC 5.4.99.2)	Cytoplasmic	6.61
A1B9R0	Pden_4190	Carbohydrate ABC transporter substrate-binding protein, CUT1 family	Periplasmic	6.52
A1B537	Pden_2544	Cytochrome c, class II	Periplasmic	6.51

A1BC00	Pden_4984	Transketolase, central region	Cytoplasmic	6.43
A1B8C8	Pden_3705	Amino acid ABC transporter substrate-binding protein, PAAT family	Periplasmic	6.07
A1BCB1	Pden_5095	Amino acid/amide ABC transporter substrate-binding protein, HAAT family	Periplasmic	5.44
A1AZY4	Pden_0716	Amino acid ABC transporter substrate-binding protein, PAAT family	Periplasmic	5.36
A1B8U5	Pden_3873	Crotonyl-CoA reductase	Cytoplasmic	5.34
A1B5Y0	Pden_2840	Acyl-CoA dehydrogenase (EC 1.3.8.-)	Cytoplasmic	5.08
A1B2B9	Pden_1563	TRAP dicarboxylate transporter-DctP subunit	Periplasmic	5.00
A1B3D6	Pden_1937	Cytochrome c, class I	Periplasmic	4.95
A1BAK2	Pden_4482	3-hydroxyisobutyrate dehydrogenase (HIBADH) (EC 1.1.1.31)	Cytoplasmic	4.90
A1BAH4	Pden_4454	Putative nitrate transport protein	Cytoplasmic/Membrane	4.74
A1BAK0	Pden_4480	Acyl-CoA dehydrogenase domain protein	Cytoplasmic	4.72
A1BA29	Pden_4309	Acetyl-CoA hydrolase (EC 3.1.2.1)	Cytoplasmic	4.63
A1B904	Pden_3932	Amino acid/amide ABC transporter substrate-binding protein, HAAT family	Periplasmic	4.61
A1BB82	Pden_4715	TRAP dicarboxylate transporter, DctP subunit	Periplasmic	4.60
A1B608	Pden_2868	Acyl-CoA dehydrogenase domain protein	Cytoplasmic	4.57
A1B0P5	Pden_0978	Uncharacterized protein	Unknown	4.53
A1AZS8	Pden_0660	Gamma-glutamyltransferase 1, Threonine peptidase, MEROPS family T03 (EC 2.3.2.2)	Periplasmic	4.40
A1B4Y8	Pden_2495	Cytochrome d1, heme region	Periplasmic	4.39
A1B0L2	Pden_0945	Substrate-binding region of ABC-type glycine betaine transport system	Periplasmic	4.32
A1BA28	Pden_4308	Lipoprotein, YaeC family	Cytoplasmic/Membrane	4.31
A1B473	Pden_2225	Propionyl-CoA synthetase (EC 6.2.1.17)	Cytoplasmic	4.27
A1AZD0	Pden_0512	Sarcosine oxidase, beta subunit family	Cytoplasmic	4.18
A1B5W3	Pden_2823	2-nitropropane dioxygenase, NPD	Unknown	4.17
A1B350	Pden_1847	Cytochrome c oxidase, cbb3-type, subunit II	Cytoplasmic	4.16
A1B1R9	Pden_1362	Periplasmic binding protein	Unknown	3.98
A1B2T9	Pden_1736	Uncharacterized protein	Periplasmic	3.93
A1B283	Pden_1527	DEAD/DEAH box helicase domain protein	Cytoplasmic/Membrane	3.90
A1AZY9	Pden_0721	Patatin	Cytoplasmic	3.86
A1B595	Pden_2602	Branched chain amino acid aminotransferase apoenzyme (EC 2.6.1.42)	Cytoplasmic	3.86
A1AYG3	Pden_0191	Glyoxalase/bleomycin resistance protein/dioxygenase	Cytoplasmic	3.86
A1B4W9	Pden_2476	Glutamine--fructose-6-phosphate transaminase (EC 2.6.1.16)	Cytoplasmic	3.84
A1B9S2	Pden_4202	Hemin-degrading family protein	Cytoplasmic	3.72
A1B2N6	Pden_1683	Putative ribose/galactose/methyl galactoside import ATP-binding protein (EC 3.6.3.17)	Cytoplasmic/Membrane	3.70
A1BB77	Pden_4710	Glucose-methanol-choline oxidoreductase	Cytoplasmic	3.70
A1AZK8	Pden_0590	Membrane protein involved in aromatic hydrocarbon degradation	Outer Membrane	3.68
A1B4K0	Pden_2354	Two component transcriptional regulator, LuxR family	Cytoplasmic	3.65
A1B2C5	Pden_1569	Extracellular solute-binding protein, family 5	Periplasmic	3.63
A1B3I8	Pden_1990	Uncharacterized protein	Unknown	3.53
A1B7P7	Pden_3470	Uncharacterized protein	Unknown	3.43

A1AZI1	Pden_0563	Citryl-CoA lyase (EC 4.1.3.34)	Cytoplasmic	3.39
A1B282	Pden_1526	Helicase domain protein	Cytoplasmic	3.13
A1B2W9	Pden_1766	Nucleoside-binding protein	Unknown	3.12
A1B5R1	Pden_2771	5'-nucleotidase, lipoprotein e(P4) family	Outer Membrane	3.10
A1B2M1	Pden_1668	ABC transporter related protein	Cytoplasmic/Membrane	3.09
A1B348	Pden_1845	Cbb3-type cytochrome c oxidase subunit CcoP (Cbb3-Cox subunit CcoP) (C-type cytochrome CcoP) (Cyt c(P)) (Cytochrome c oxidase subunit III)	Cytoplasmic	3.07
A1AZT2	Pden_0664	TRAP transporter solute receptor, TAXI family	Unknown	3.06
A1B4Z6	Pden_2503	Asparaginase (EC 3.5.1.1)	Cytoplasmic	3.02
A1B5B6	Pden_2623	Uncharacterized protein	Unknown	2.99
A1AZ54	Pden_0434	Acyl-CoA dehydrogenase domain protein	Cytoplasmic	2.97
A1B951	Pden_3979	Enoyl-CoA hydratase (EC 4.2.1.17)	Cytoplasmic	2.97
A1B5G4	Pden_2671	Amidase	Cytoplasmic	2.92
A1B7V3	Pden_3527	TonB-dependent receptor	Outer Membrane	2.91
A1AYH6	Pden_0206	Short-chain dehydrogenase/reductase SDR	Cytoplasmic	2.90
A1B1F9	Pden_1248	Extracellular solute-binding protein, family 5	Periplasmic	2.88
A1AZF5	Pden_0537	Peptidoglycan-binding domain 1 protein	Cytoplasmic	2.88
A1B6B9	Pden_2980	Periplasmic binding protein	Unknown	2.88
A1AY09	Pden_0036	NlpA lipoprotein	Unknown	2.83
A1AZM6	Pden_0608	Extracellular solute-binding protein, family 5	Periplasmic	2.82
A1AZI4	Pden_0566	L-erythro-3-methylmalyl-CoA dehydratase (EC 4.2.1.-)	Cytoplasmic	2.82
A1B878	Pden_3655	Glycosyl transferase, group 1	Cytoplasmic	2.81
A1B622	Pden_2882	5'-Nucleotidase domain protein	Periplasmic	2.79
A1B400	Pden_2152	Extracellular solute-binding protein, family 1	Periplasmic	2.78
A1B0J3	Pden_0926	Uncharacterized protein	Unknown	2.74
A1B593	Pden_2600	Two component transcriptional regulator, winged helix family	Cytoplasmic	2.73
A1B9V3	Pden_4233	Respiratory nitrate reductase gamma subunit	Cytoplasmic/Membrane	2.67
A1B0L7	Pden_0950	Fervidolysin, Serine peptidase, MEROPS family S08A	Cytoplasmic	2.65
A1B452	Pden_2204	Uncharacterized protein	Unknown	2.63
A1B2M0	Pden_1667	ABC transporter related protein	Cytoplasmic/Membrane	2.63
A1B9V6	Pden_4236	Respiratory nitrate reductase alpha subunit apoprotein (NarI)	Cytoplasmic/Membrane	2.61
A1B284	Pden_1528	Uncharacterized protein	Cytoplasmic	2.59
A1B1L6	Pden_1305	Antibiotic biosynthesis monooxygenase	Unknown	2.58
A1BB44	Pden_4677	PKHD-type hydroxylase Pden_4677 (EC 1.14.11.-)	Cytoplasmic	2.57
A1BAM2	Pden_4502	ABC polyamine transporter, periplasmic substrate-binding protein	Periplasmic	2.57
A1B859	Pden_3636	OmpW family protein	Outer Membrane	2.56
A1B2B3	Pden_1557	Riboflavin synthase, alpha subunit	Cytoplasmic	2.56
A1B2S2	Pden_1719	AMP-dependent synthetase and ligase	Cytoplasmic	2.55
A1B9W4	Pden_4244	Asparaginase (EC 3.5.1.1)	Unknown	2.55
A1BCB9	Pden_5103	Poly-beta-hydroxybutyrate polymerase domain protein	Cytoplasmic	2.48
A1B8A9	Pden_3686	Uncharacterized protein	Unknown	2.47

A1B061	Pden_0793	L-aspartate-binding protein / L-glutamate-binding protein / L-glutamine-binding protein / L-asparagine-binding protein	Unknown	2.44
A1B607	Pden_2867	Transcriptional regulator, MerR family	Cytoplasmic	2.44
A1B0M5	Pden_0958	Poly(R)-hydroxyalkanoic acid synthase, class I	Cytoplasmic	2.44
A1B1N5	Pden_1324	Oligopeptide/dipeptide ABC transporter, ATPase subunit	Cytoplasmic/Membrane	2.43
A1B357	Pden_1854	Uncharacterized protein	Unknown	2.42
A1B9P3	Pden_4173	TonB-dependent receptor	Outer Membrane	2.39
A1B368	Pden_1865	2-deoxycytidine 5-triphosphate deaminase	Cytoplasmic	2.39
A1B1G6	Pden_1255	Short-chain dehydrogenase/reductase SDR	Cytoplasmic	2.38
A1B347	Pden_1844	4Fe-4S ferredoxin, iron-sulfur binding domain protein	Cytoplasmic/Membrane	2.35
A1B8Y2	Pden_3910	Endoribonuclease L-PSP	Cytoplasmic	2.33
A1AZV4	Pden_0686	Protein TolB	Periplasmic	2.33
A1B9V5	Pden_4235	Respiratory nitrate reductase beta subunit (NarH)	Cytoplasmic/Membrane	2.31
A1B3V3	Pden_2105	Transcriptional regulator, GntR family	Cytoplasmic	2.29
A1B9V7	Pden_4237	Nitrite transporter	Cytoplasmic/Membrane	2.28
A1B9C1	Pden_4049	Uncharacterized protein	Cytoplasmic	2.27
A1B611	Pden_2871	3-hydroxyacyl-CoA dehydrogenase (EC 1.1.1.35)	Cytoplasmic	2.24
A1B559	Pden_2566	Uncharacterized protein	Unknown	2.24
A1B8D0	Pden_3707	Uncharacterized protein	Unknown	2.23
A1BCF0	Pden_5134	Uncharacterized protein	Unknown	2.22
A1B9S1	Pden_4201	TonB-dependent heme/hemoglobin receptor family protein	OuterMembrane	2.20
A1B2W6	Pden_1763	Nucleoside ABC transporter ATP-binding protein	CytoplasmicMembrane	2.19
A1B004	Pden_0736	Glyoxalase/bleomycin resistance protein/dioxygenase	Unknown	2.17
A1B428	Pden_2180	GCN5-related N-acetyltransferase	Unknown	2.16
A1B973	Pden_4001	Purine nucleosidase (EC 3.2.2.1)	Cytoplasmic	2.15
A1B3Z9	Pden_2151	Polyamine ABC trasnporter, periplasmic polyamine-binding protein	Periplasmic	2.15
A1B054	Pden_0786	Transcriptional regulator, LuxR family	Unknown	2.12
A1B574	Pden_2581	ErfK/YbiS/YcfS/YnhG family protein	Unknown	2.08
A1BAE7	Pden_4427	Glucose-1-phosphate adenylyltransferase (EC 2.7.7.27) (ADP-glucose pyrophosphorylase) (ADPGlc PPase) (ADP-glucose synthase)	Cytoplasmic	2.08
A1B076	Pden_0808	Uncharacterized protein	Unknown	2.06
A1B3U3	Pden_2095	DSBA oxidoreductase	Unknown	2.06
Q51700	Pden_2487	Nitrite reductase (EC 1.7.2.1) (Cytochrome cd1) (Cytochrome oxidase) (Hydroxylamine reductase) (EC 1.7.99.1)	Periplasmic	2.05
A1B6E6	Pden_3007	TonB-dependent siderophore receptor	Outer Membrane	2.05
A1B1N7	Pden_1326	Binding-protein-dependent transport systems inner membrane component	Cytoplasmic/Membrane	2.05
A1B2T2	Pden_1729	Alanine dehydrogenase (EC 1.4.1.1)	Cytoplasmic	2.05
A1B3M5	Pden_2027	3-oxoacyl-[acyl-carrier-protein] reductase (EC 1.1.1.100)	Cytoplasmic	2.03
A1B9P1	Pden_4171	Uncharacterized protein	Unknown	2.00

A1B1X2	Pden_1415	Protein-export membrane protein SecF	Cytoplasmic/Membrane	-2.00
A1B8K8	Pden_3786	30S ribosomal protein S1	Cytoplasmic	-2.00
A1B4X4	Pden_2481	von Willebrand factor, type A	Unknown	-2.01
A1B035	Pden_0767	50S ribosomal protein L29	Cytoplasmic	-2.01
A1B0E5	Pden_0878	MOSC domain containing protein	Cytoplasmic	-2.01
A1B8V6	Pden_3884	50S ribosomal protein L19	Cytoplasmic	-2.01
A1B5N2	Pden_2742	Uncharacterized protein	Unknown	-2.02
A1B042	Pden_0774	50S ribosomal protein L6	Cytoplasmic	-2.02
A1AZH7	Pden_0559	Succinate--CoA ligase [ADP-forming] subunit beta (EC 6.2.1.5) (Succinyl-CoA synthetase subunit beta) (SCS-beta)	Cytoplasmic	-2.02
A1B5W1	Pden_2821	Uncharacterized protein	Unknown	-2.02
A1B684	Pden_2944	Phosphomethylpyrimidine synthase (EC 4.1.99.17) (Hydroxymethylpyrimidine phosphate synthase) (HMP-P synthase) (HMP-phosphate synthase) (HMPP synthase) (Thiamine biosynthesis protein ThiC)	Cytoplasmic	-2.03
A1B9I1	Pden_4109	Putative sulfonate/nitrate transport system substrate-binding protein	Cytoplasmic	-2.03
A1B4B5	Pden_2267	30S ribosomal protein S21	Unknown	-2.07
A1B080	Pden_0812	Phosphoserine phosphatase (EC 3.1.3.3)	Cytoplasmic	-2.10
A1B2U3	Pden_1740	Uncharacterized protein	Cytoplasmic	-2.10
A1B2X1	Pden_1768	Acyl carrier protein (ACP)	Cytoplasmic	-2.12
A1B013	Pden_0745	50S ribosomal protein L10	Cytoplasmic	-2.14
A1AZS5	Pden_0657	Uncharacterized protein	Unknown	-2.15
A1B4S6	Pden_2433	50S ribosomal protein L28	Cytoplasmic	-2.15
A1B011	Pden_0743	50S ribosomal protein L11	Cytoplasmic	-2.15
A1B0F4	Pden_0887	Trigger factor (TF) (EC 5.2.1.8) (PPIase)	Cytoplasmic	-2.17
A1AZP4	Pden_0626	OmpA/MotB domain protein	Cytoplasmic/Membrane	-2.17
A1B021	Pden_0753	30S ribosomal protein S12	Cytoplasmic	-2.17
A1BC28	Pden_5012	ABC transporter substrate-binding protein	Unknown	-2.18
A1B877	Pden_3654	60 kDa chaperonin (GroEL protein) (Protein Cpn60)	Cytoplasmic	-2.18
A1BAG4	Pden_4444	Uncharacterized protein	Unknown	-2.20
A1AZY8	Pden_0720	NADH:flavin oxidoreductase/NADH oxidase	Cytoplasmic	-2.20
A1B5S5	Pden_2785	FxsA cytoplasmic membrane protein	Cytoplasmic/Membrane	-2.20
A1B032	Pden_0764	50S ribosomal protein L22	Cytoplasmic	-2.21
A1B044	Pden_0776	30S ribosomal protein S5	Cytoplasmic	-2.22
A1B045	Pden_0777	50S ribosomal protein L30	Unknown	-2.24
A1BAH8	Pden_4458	RNA binding S1 domain protein	Cytoplasmic	-2.24
A1B358	Pden_1855	ABC transporter related protein	Cytoplasmic/Membrane	-2.28
A1AZ38	Pden_0418	GTPase Era	Cytoplasmic/Membrane	-2.28
A1BAB3	Pden_4393	Glycerol-3-phosphate dehydrogenase (EC 1.1.5.3)	Cytoplasmic	-2.30
A1AZN6	Pden_0618	Bifunctional protein GlmU [Includes: UDP-N-acetylglucosamine pyrophosphorylase (EC 2.7.7.23) (N-acetylglucosamine-1-phosphate uridyltransferase); Glucosamine-1-phosphate N-acetyltransferase (EC 2.3.1.157)]	Cytoplasmic	-2.30
A1B050	Pden_0782	30S ribosomal protein S13	Cytoplasmic	-2.31

A1B3D4	Pden_1935	Methionine synthase (B12-dependent) (EC 2.1.1.13)	Cytoplasmic	-2.35
A1B5G6	Pden_2673	DNA translocase FtsK	Cytoplasmic/Membrane	-2.36
A1B8E8	Pden_3725	Elongation factor Ts (EF-Ts)	Cytoplasmic	-2.37
A1BC44	Pden_5028	Uncharacterized protein	Cytoplasmic	-2.38
A1B5W0	Pden_2820	RNA polymerase sigma factor	Cytoplasmic	-2.39
Q51703	Pden_2491	Protein NirD	Cytoplasmic	-2.39
A1BA97	Pden_4377	Ribonucleoside-diphosphate reductase (EC 1.17.4.1)	Cytoplasmic	-2.43
A1BB80	Pden_4713	Probable septum site-determining protein MinC	Cytoplasmic	-2.48
A1B5T0	Pden_2790	ATP-dependent protease ATPase subunit HslU (Unfoldase HslU)	Cytoplasmic	-2.49
A1B053	Pden_0785	50S ribosomal protein L17	Cytoplasmic	-2.54
A1B339	Pden_1836	DNA topoisomerase 4 subunit B (EC 5.99.1.3) (Topoisomerase IV subunit B)	Cytoplasmic	-2.55
A1B371	Pden_1868	Beta-N-acetylhexosaminidase (EC 3.2.1.52)	Cytoplasmic	-2.60
A1B082	Pden_0814	D-3-phosphoglycerate dehydrogenase (EC 1.1.1.95)	Cytoplasmic	-2.69
A1B6B0	Pden_2970	30S ribosomal protein S9	Cytoplasmic	-2.74
A1BAK7	Pden_4487	Cell division protein FtsZ	Cytoplasmic	-2.78
Q51664	Pden_2482	Protein NorQ	Cytoplasmic	-2.82
A1B716	Pden_3229	Uncharacterized protein	Cytoplasmic	-2.83
A1B034	Pden_0766	50S ribosomal protein L16	Cytoplasmic	-2.84
A1B1T9	Pden_1382	Protease Do	Periplasmic	-2.87
A1B1W0	Pden_1403	Uncharacterized protein	Cytoplasmic	-2.89
A1B0J7	Pden_0930	Uncharacterized protein	Cytoplasmic	-2.90
A1BAD6	Pden_4416	Succinate semialdehyde dehydrogenase (EC 1.2.1.16)	Cytoplasmic	-2.96
A1B038	Pden_0770	50S ribosomal protein L24	Cytoplasmic	-2.96
A1B014	Pden_0746	50S ribosomal protein L7/L12	Unknown	-2.99
A1BAN4	Pden_4514	30S ribosomal protein S20	Cytoplasmic	-3.00
A1BAL4	Pden_4494	UDP-N-acetylmuramate--L-alanine ligase (EC 6.3.2.8) (UDP-N-acetylmuramoyl-L-alanine synthetase)	Cytoplasmic	-3.05
A1B3D1	Pden_1932	Uncharacterized protein	Periplasmic	-3.12
A1B4Q7	Pden_2413	ABC transporter related protein	Cytoplasmic/Membrane	-3.15
A1B4Q6	Pden_2412	Type I secretion membrane fusion protein, HlyD family	Cytoplasmic/Membrane	-3.30
A1B869	Pden_3646	Heat shock protein Hsp20	Cytoplasmic	-3.43
Q51701	Pden_2488	Uroporphyrinogen-III C-methyltransferase (Urogen III methylase) (EC 2.1.1.107) (SUMT) (Uroporphyrinogen III methylase) (UROM)	Cytoplasmic	-3.51
A1B040	Pden_0772	30S ribosomal protein S14	Cytoplasmic	-3.53
A1B0F7	Pden_0890	30S ribosomal protein S18	Cytoplasmic	-3.68
A1B4Q1	Pden_2407	Mur ligase, middle domain protein	Cytoplasmic/Membrane	-4.08
A1B683	Pden_2943	RND efflux system, outer membrane lipoprotein, NodT family	Outer Membrane	-4.13
A1BCD5	Pden_5119	NADPH-dependent FMN reductase	Unknown	-4.16
A1B4Q5	Pden_2411	Outer membrane efflux protein	Cytoplasmic	-4.19
A1BB78	Pden_4711	Cell division topological specificity factor	Cytoplasmic	-4.21
A1BC33	Pden_5017	Uncharacterized protein	Unknown	-4.22
A1B372	Pden_1869	Sporulation domain protein	Unknown	-4.76
A1B5Q0	Pden_2760	30S ribosomal protein S15	Cytoplasmic	-4.83
A1BA98	Pden_4378	Ribonucleoside-diphosphate reductase subunit beta (EC 1.17.4.1)	Cytoplasmic	-4.87

A1BC29	Pden_5013	Alkanesulfonate monooxygenase (EC 1.14.14.5)	Cytoplasmic	-5.20
A1B8A8	Pden_3685	Uncharacterized protein	Unknown	-5.39
A1BAG3	Pden_4443	Electron transport protein SCO1/SenC	Unknown	-6.08
A1BCE1	Pden_5125	Putative monooxygenase protein	Cytoplasmic	-7.14
A1B0B7	Pden_0849	Putative outer membrane protein	Outer Membrane	-7.73
A1B2A7	Pden_1551	Uncharacterized protein	Outer Membrane	-7.75
A1AZG3	Pden_0545	Phosphoglucosamine mutase (EC 5.4.2.10)	Cytoplasmic	-9.58
A1B4Q4	Pden_2410	Uncharacterized protein	Cytoplasmic	-10.24
A1BBD2	Pden_4765	C4-dicarboxylate transport protein	Cytoplasmic/Membrane	-32.28
A1BC23	Pden_5007	Monooxygenase, NtaA/SnaA/SoxA family	Cytoplasmic	-39.93
A1AXY8	Pden_0015	Glutathione-dependent formaldehyde-activating enzyme (EC 4.4.1.22) (S-(hydroxymethyl)glutathione synthase)	Cytoplasmic	Exclusive pH 7.2
A1AXZ7	Pden_0024	Uncharacterized protein	Unknown	Exclusive pH 7.2
A1AXZ9	Pden_0026	Amino acid/amide ABC transporter substrate-binding protein, HAAT family	Unknown	Exclusive pH 7.2
A1AY00	Pden_0027	40-residue YVTN family beta-propeller repeat protein	Unknown	Exclusive pH 7.2
A1AY21	Pden_0048	AMP-dependent synthetase and ligase	Cytoplasmic	Exclusive pH 7.2
A1AY22	Pden_0049	Amino acid/amide ABC transporter ATP-binding protein 1, HAAT family	Cytoplasmic/Membrane	Exclusive pH 7.2
A1AY26	Pden_0053	Amino acid/amide ABC transporter ATP-binding protein 2, HAAT family	Cytoplasmic/Membrane	Exclusive pH 7.2
A1AY27	Pden_0054	Phenylacetate-CoA ligase, putative	Cytoplasmic	Exclusive pH 7.2
A1AY32	Pden_0059	Glutathione S-transferase, N-terminal domain	Cytoplasmic/Membrane	Exclusive pH 7.2
A1AY34	Pden_0061	Uncharacterized protein	Unknown	Exclusive pH 7.2
A1AY52	Pden_0079	Two component transcriptional regulator, winged helix family	Unknown	Exclusive pH 7.2
A1AYG0	Pden_0188	Regulatory protein, IclR	Cytoplasmic	Exclusive pH 7.2
A1AYG5	Pden_0193	TRAP dicarboxylate transporter-DctP subunit	Unknown	Exclusive pH 7.2
A1AYK9	Pden_0239	Extracellular solute-binding protein, family 5	Periplasmic	Exclusive pH 7.2
A1AYQ1	Pden_0281	Hydroxyectoine-binding protein / ectoine-binding protein	Periplasmic	Exclusive pH 7.2
A1AYS6	Pden_0306	Uncharacterized protein	Cytoplasmic/Membrane	Exclusive pH 7.2
A1AYS8	Pden_0308	Uncharacterized protein UPF0065	Periplasmic	Exclusive pH 7.2
A1AYS9	Pden_0309	Periplasmic iron-binding protein	Unknown	Exclusive pH 7.2
A1AYV2	Pden_0332	Glutathione S-transferase, N-terminal domain	Cytoplasmic	Exclusive pH 7.2
A1AZ21	Pden_0401	SH3, type 3 domain protein	Cytoplasmic/Membrane	Exclusive pH 7.2
A1AZ64	Pden_0444	Uncharacterized protein	Cytoplasmic	Exclusive pH 7.2
A1AZ67	Pden_0447	Uncharacterized protein	Cytoplasmic	Exclusive pH 7.2

A1AZ68	Pden_0448	Uncharacterized protein	Cytoplasmic	Exclusive pH 7.2
A1AZA7	Pden_0489	Uncharacterized protein	Unknown	Exclusive pH 7.2
A1AZE2	Pden_0524	Purine nucleoside phosphorylase (EC 2.4.2.1) (Inosine-guanosine phosphorylase)	Cytoplasmic	Exclusive pH 7.2
A1AZE5	Pden_0527	Nucleoside ABC transporter ATP-binding protein	Cytoplasmic/Membrane	Exclusive pH 7.2
A1AZI3	Pden_0565	Uncharacterized protein	Unknown	Exclusive pH 7.2
A1AZQ0	Pden_0632	SsrA-binding protein (Small protein B)	Cytoplasmic	Exclusive pH 7.2
A1AZQ6	Pden_0638	Uncharacterized protein	Cytoplasmic/Membrane	Exclusive pH 7.2
A1AZT7	Pden_0669	3-oxoacyl-[acyl-carrier-protein] synthase III (EC 2.3.1.41)	Cytoplasmic	Exclusive pH 7.2
A1AZU8	Pden_0680	Transcriptional regulator, GntR family	Cytoplasmic	Exclusive pH 7.2
A1AZU9	Pden_0681	Uncharacterized protein	Unknown	Exclusive pH 7.2
A1AZW5	Pden_0697	Multisubunit potassium/proton antiporter, PhaB subunit / multisubunit potassium/proton antiporter, PhaA subunit (EC 1.6.99.5)	Cytoplasmic/Membrane	Exclusive pH 7.2
A1B085	Pden_0817	CreA family protein	Unknown	Exclusive pH 7.2
A1B096	Pden_0828	Hydratase/decarboxylase	Cytoplasmic/Membrane	Exclusive pH 7.2
A1B0B5	Pden_0847	Polysaccharide biosynthesis protein	Cytoplasmic/Membrane	Exclusive pH 7.2
A1B0L0	Pden_0943	Bifunctional protein PutA	Cytoplasmic	Exclusive pH 7.2
A1B0L9	Pden_0952	DSBA oxidoreductase	Cytoplasmic	Exclusive pH 7.2
A1B0W3	Pden_1046	Transcriptional regulator, LacI family	Cytoplasmic	Exclusive pH 7.2
A1B0W4	Pden_1047	Carbohydrate ABC transporter substrate-binding protein, CUT1 family	Unknown	Exclusive pH 7.2
A1B0Y2	Pden_1067	OsmC family protein	Cytoplasmic	Exclusive pH 7.2
A1B1F1	Pden_1240	Peptidoglycan-binding domain 1 protein	Unknown	Exclusive pH 7.2
A1B1F4	Pden_1243	3-oxoacyl-[acyl-carrier-protein] synthase II (EC 2.3.1.41)	Cytoplasmic/Membrane	Exclusive pH 7.2
A1B1G2	Pden_1251	ABC transporter related protein	Cytoplasmic/Membrane	Exclusive pH 7.2
A1B1H0	Pden_1259	Periplasmic solute binding protein	Periplasmic	Exclusive pH 7.2
A1B1I8	Pden_1277	Uracil-xanthine permease	Cytoplasmic/Membrane	Exclusive pH 7.2
A1B1I9	Pden_1278	Uncharacterized protein	Cytoplasmic/Membrane	Exclusive pH 7.2
A1B1S0	Pden_1363	Isocitrate lyase (EC 4.1.3.1)	Cytoplasmic	Exclusive pH 7.2
A1B1S1	Pden_1364	Malate synthase (EC 2.3.3.9)	Cytoplasmic	Exclusive pH 7.2
A1B1U4	Pden_1387	Inositol monophosphatase	Cytoplasmic	Exclusive pH 7.2
A1B1Z5	Pden_1438	Pathogenesis-related protein	Cytoplasmic	Exclusive pH 7.2

A1B1Z6	Pden_1439	Uncharacterized protein	Cytoplasmic	Exclusive pH 7.2
A1B280	Pden_1524	Putative chromosome segregation SMC protein	Cytoplasmic	Exclusive pH 7.2
A1B2C1	Pden_1565	Tripartite ATP-independent periplasmic transporter, DctQ component	Cytoplasmic/Membrane	Exclusive pH 7.2
A1B2M3	Pden_1670	Binding-protein-dependent transport systems inner membrane component	Cytoplasmic/Membrane	Exclusive pH 7.2
A1B2N4	Pden_1681	Monosaccharide ABC transporter substrate-binding protein, CUT2 family	Periplasmic	Exclusive pH 7.2
A1B2S6	Pden_1723	Uncharacterized protein	Unknown	Exclusive pH 7.2
A1B2T6	Pden_1733	Iron permease FTR1	Cytoplasmic/Membrane	Exclusive pH 7.2
A1B336	Pden_1833	Peptidase M19, renal dipeptidase	Unknown	Exclusive pH 7.2
A1B3J8	Pden_2000	YCII-related protein	Unknown	Exclusive pH 7.2
A1B3M1	Pden_2023	Redoxin domain protein	Cytoplasmic/Membrane	Exclusive pH 7.2
A1B3N4	Pden_2036	Type I secretion system ATPase	Cytoplasmic/Membrane	Exclusive pH 7.2
A1B3U6	Pden_2098	Alkane 1-monooxygenase (EC 1.14.15.3)	Cytoplasmic/Membrane	Exclusive pH 7.2
A1B3Y2	Pden_2134	Aminotransferase (EC 2.6.1.-)	Cytoplasmic	Exclusive pH 7.2
A1B445	Pden_2197	Export-related chaperone CsaA	Cytoplasmic	Exclusive pH 7.2
A1B470	Pden_2222	Thymidine phosphorylase (EC 2.4.2.4)	Unknown	Exclusive pH 7.2
A1B471	Pden_2223	Cytidine deaminase (EC 3.5.4.5)	Cytoplasmic	Exclusive pH 7.2
A1B482	Pden_2234	NADH-quinone oxidoreductase subunit K (EC 1.6.5.11) (NADH dehydrogenase I subunit K) (NADH dehydrogenase I, subunit 11) (NADH-quinone oxidoreductase subunit 11) (NQO11) (NDH-1 subunit K) (NDH-1, subunit 11)	Cytoplasmic/Membrane	Exclusive pH 7.2
A1B4A1	Pden_2253	Glutathione-dependent formaldehyde-activating, GFA	Unknown	Exclusive pH 7.2
A1B4D4	Pden_2287	Membrane protein of uknown function UCP014873	Cytoplasmic/Membrane	Exclusive pH 7.2
A1B4F5	Pden_2308	Ribokinase (RK) (EC 2.7.1.15)	Cytoplasmic	Exclusive pH 7.2
A1B4G9	Pden_2322	Transcriptional regulator, AsnC family	Cytoplasmic	Exclusive pH 7.2
A1B4T4	Pden_2441	Serine/threonine protein kinase	Cytoplasmic	Exclusive pH 7.2
A1B4T7	Pden_2444	Uncharacterized protein	Cytoplasmic	Exclusive pH 7.2
A1B522	Pden_2529	Cob(I)yrinic acid a,c-diamide adenosyltransferase (EC 2.5.1.17)	Cytoplasmic	Exclusive pH 7.2
A1B530	Pden_2537	Precorrin-6A reductase (EC 1.3.1.54)	Cytoplasmic	Exclusive pH 7.2
A1B560	Pden_2567	Periplasmic binding protein	Unknown	Exclusive pH 7.2
A1B5B5	Pden_2622	Uncharacterized protein	Unknown	Exclusive pH 7.2
A1B5E6	Pden_2653	Efflux transporter, RND family, MFP subunit	Unknown	Exclusive pH 7.2

A1B5E7	Pden_2654	Efflux transporter, RND family, MFP subunit	Unknown	Exclusive pH 7.2
A1B5E8	Pden_2655	Acriflavin resistance protein	Cytoplasmic/Membrane	Exclusive pH 7.2
A1B5F2	Pden_2659	Murein endopeptidase, Metallo peptidase, MEROPS family M74	Periplasmic	Exclusive pH 7.2
A1B5F7	Pden_2664	Putative transcriptional regulator, ModE family	Unknown	Exclusive pH 7.2
A1B5I2	Pden_2689	Flavin reductase domain protein, FMN-binding protein	Unknown	Exclusive pH 7.2
A1B5M0	Pden_2730	Protein-L-isoaspartate O-methyltransferase (EC 2.1.1.77) (L-isoaspartyl protein carboxyl methyltransferase) (Protein L-isoaspartyl methyltransferase) (Protein-beta-aspartate methyltransferase) (PIMT)	Cytoplasmic	Exclusive pH 7.2
A1B5N5	Pden_2745	Multiple monosaccharide-binding protein	Periplasmic	Exclusive pH 7.2
A1B5Q9	Pden_2769	Glutathione S-transferase, N-terminal domain	Cytoplasmic	Exclusive pH 7.2
A1B5U2	Pden_2802	Uncharacterized protein	Unknown	Exclusive pH 7.2
A1B5W6	Pden_2826	Protein FdhE homolog	Cytoplasmic	Exclusive pH 7.2
A1B645	Pden_2905	Short chain enoyl-CoA hydratase (EC 4.2.1.17)	Cytoplasmic	Exclusive pH 7.2
A1B649	Pden_2909	AMP-dependent synthetase and ligase	Cytoplasmic	Exclusive pH 7.2
A1B690	Pden_2950	Uncharacterized protein	Unknown	Exclusive pH 7.2
A1B693	Pden_2953	Transcriptional regulator, AraC family with amidase-like domain	Cytoplasmic/Membrane	Exclusive pH 7.2
A1B6A7	Pden_2967	Enoyl-CoA hydratase/isomerase	Cytoplasmic	Exclusive pH 7.2
A1B750	Pden_3266	Uncharacterized protein UPF0065	Unknown	Exclusive pH 7.2
A1B751	Pden_3267	Amidohydrolase 2	Cytoplasmic	Exclusive pH 7.2
A1B7G8	Pden_3389	Transcriptional regulator, GntR family	Cytoplasmic	Exclusive pH 7.2
A1B7I1	Pden_3404	Carbohydrate ABC transporter substrate-binding protein, CUT1 family	Periplasmic	Exclusive pH 7.2
A1B7L4	Pden_3437	Fumarylacetoacetate (FAA) hydrolase	Cytoplasmic	Exclusive pH 7.2
A1B7R6	Pden_3490	4-hydroxybenzoate 3-monooxygenase (EC 1.14.13.2)	Cytoplasmic	Exclusive pH 7.2
A1B7S3	Pden_3497	TRAP dicarboxylate transporter-DctP subunit	Periplasmic	Exclusive pH 7.2
A1B7V7	Pden_3531	Periplasmic binding protein	Unknown	Exclusive pH 7.2
A1B7Z3	Pden_3569	Formamidase (EC 3.5.1.49)	Cytoplasmic	Exclusive pH 7.2
A1B810	Pden_3586	Transglutaminase domain protein	Unknown	Exclusive pH 7.2
A1B822	Pden_3599	Uncharacterized protein	Unknown	Exclusive pH 7.2
A1B857	Pden_3634	Uncharacterized protein	Unknown	Exclusive pH 7.2
A1B861	Pden_3638	Appr-1-p processing domain protein	Cytoplasmic	Exclusive pH 7.2
A1B864	Pden_3641	Hydroxymethylglutaryl-CoA lyase (EC 4.1.3.4)	Cytoplasmic	Exclusive pH 7.2

A1B879	Pden_3656	GumN family protein	Unknown	Exclusive pH 7.2
A1B884	Pden_3661	MaoC domain protein dehydratase	Cytoplasmic	Exclusive pH 7.2
A1B8C2	Pden_3699	Heme A synthase (HAS) (EC 1.3.-.-) (Cytochrome aa3-controlling protein) (ctaA)	Cytoplasmic/Membrane	Exclusive pH 7.2
A1B8D1	Pden_3708	Uncharacterized protein	Unknown	Exclusive pH 7.2
A1B8G6	Pden_3743	Uncharacterized protein	Cytoplasmic	Exclusive pH 7.2
A1B8L6	Pden_3794	Uncharacterized protein	Cytoplasmic	Exclusive pH 7.2
A1B8L9	Pden_3797	Transcriptional regulator, RpiR family	Cytoplasmic	Exclusive pH 7.2
A1B8P6	Pden_3824	CoA-binding domain protein	Cytoplasmic	Exclusive pH 7.2
A1B8S2	Pden_3850	Uncharacterized protein	Unknown	Exclusive pH 7.2
A1B8U3	Pden_3871	Uncharacterized protein	Cytoplasmic	Exclusive pH 7.2
A1B8W0	Pden_3888	Transcriptional regulator, Fis family	Unknown	Exclusive pH 7.2
A1B949	Pden_3977	Cytochrome c-type biogenesis protein CcmF	Cytoplasmic/Membrane	Exclusive pH 7.2
A1B9D3	Pden_4061	Uncharacterized protein	Unknown	Exclusive pH 7.2
A1B9E5	Pden_4073	DNA primase (EC 2.7.7.-)	Cytoplasmic	Exclusive pH 7.2
A1B9H6	Pden_4104	L-carnitine dehydratase/bile acid-inducible protein F	Cytoplasmic	Exclusive pH 7.2
A1B9I2	Pden_4110	HpcH/HpaI aldolase	Cytoplasmic	Exclusive pH 7.2
A1B9I3	Pden_4111	Uncharacterized protein	Cytoplasmic	Exclusive pH 7.2
A1B9I4	Pden_4112	L-carnitine dehydratase/bile acid-inducible protein F	Cytoplasmic	Exclusive pH 7.2
A1B9I6	Pden_4116	Uncharacterized protein	Cytoplasmic/Membrane	Exclusive pH 7.2
A1B9I7	Pden_4117	Pimeloyl-CoA biosynthesis protein BioC	Cytoplasmic	Exclusive pH 7.2
A1B9I8	Pden_4118	Transcriptional regulator, LysR family	Cytoplasmic	Exclusive pH 7.2
A1B9I9	Pden_4119	Fumarate hydratase class I (EC 4.2.1.2)	Cytoplasmic	Exclusive pH 7.2
A1B9J0	Pden_4120	TRAP dicarboxylate transporter, DctM subunit	Cytoplasmic/Membrane	Exclusive pH 7.2
A1B9J1	Pden_4121	Tripartite ATP-independent periplasmic transporter, DctQ component	Cytoplasmic/Membrane	Exclusive pH 7.2
A1B9J2	Pden_4122	TRAP dicarboxylate transporter, DctP subunit	Periplasmic	Exclusive pH 7.2
A1B9S3	Pden_4203	Periplasmic binding protein	Unknown	Exclusive pH 7.2
A1B9S8	Pden_4208	Acetate kinase (EC 2.7.2.1) (Acetokinase)	Cytoplasmic	Exclusive pH 7.2
A1B9T2	Pden_4212	Uncharacterized protein	Cytoplasmic/Membrane	Exclusive pH 7.2
A1BA20	Pden_4300	Uncharacterized protein	Unknown	Exclusive pH 7.2
A1BAB8	Pden_4398	FAD linked oxidase domain protein	Cytoplasmic	Exclusive pH 7.2
A1BAC2	Pden_4402	Uncharacterized protein	Cytoplasmic/Membrane	Exclusive pH 7.2

A1BAC4	Pden_4404	Uncharacterized protein UPF0065	Unknown	Exclusive pH 7.2
A1BAD2	Pden_4412	Acyl-CoA dehydrogenase domain protein	Cytoplasmic	Exclusive pH 7.2
A1BAH0	Pden_4450	Formate/nitrite transporter	Cytoplasmic/Membrane	Exclusive pH 7.2
A1BAS1	Pden_4552	Na+/solute symporter	Cytoplasmic/Membrane	Exclusive pH 7.2
A1BAX2	Pden_4603	Putative ABC transporter binding protein component	Periplasmic	Exclusive pH 7.2
A1BB09	Pden_4642	Uncharacterized protein	Unknown	Exclusive pH 7.2
A1BB12	Pden_4645	Transcriptional regulator, TetR family	Cytoplasmic	Exclusive pH 7.2
A1BB21	Pden_4654	HAD superfamily (Subfamily IIIB) phosphatase, TIGR01672 (EC 3.1.3.2)	Unknown	Exclusive pH 7.2
A1BB52	Pden_4685	Secretion protein HlyD family protein	Cytoplasmic/Membrane	Exclusive pH 7.2
A1BB61	Pden_4694	FAD-dependent pyridine nucleotide-disulfide oxidoreductase	Periplasmic	Exclusive pH 7.2
A1BB94	Pden_4727	Glucose sorbosone dehydrogenase	Unknown	Exclusive pH 7.2
A1BBC5	Pden_4758	Branched-chain alpha-keto acid dehydrogenase E1 component (EC 1.2.4.4)	Cytoplasmic	Exclusive pH 7.2
A1BBD8	Pden_4771	Monosaccharide ABC transporter substrate-binding protein, CUT2 family	Periplasmic	Exclusive pH 7.2
A1BBG4	Pden_4797	Amino acid/amide ABC transporter substrate-binding protein, HAAT family	Periplasmic	Exclusive pH 7.2
A1BBG8	Pden_4801	Phenylacetate-coenzyme A ligase (EC 6.2.1.30) (Phenylacetyl-CoA ligase)	Cytoplasmic	Exclusive pH 7.2
A1BBI9	Pden_4822	Short-chain dehydrogenase/reductase SDR	Cytoplasmic	Exclusive pH 7.2
A1BBL2	Pden_4846	Periplasmic binding protein/LacI transcriptional regulator	Cytoplasmic	Exclusive pH 7.2
A1BBN9	Pden_4873	Putative spermidine/putrescine transport system substrate-binding protein	Periplasmic	Exclusive pH 7.2
A1BBQ4	Pden_4888	Substrate-binding region of ABC-type glycine betaine transport system	Cytoplasmic/Membrane	Exclusive pH 7.2
A1BBZ8	Pden_4982	Short-chain dehydrogenase/reductase SDR	Unknown	Exclusive pH 7.2
A1BBZ9	Pden_4983	Alpha/beta hydrolase fold protein	Cytoplasmic	Exclusive pH 7.2
A1BCB8	Pden_5102	ATPase, P-type (Transporting), HAD superfamily, subfamily IC	Cytoplasmic/Membrane	Exclusive pH 7.2
A1BCC6	Pden_5110	Putative signal-transduction protein with CBS domains	Cytoplasmic	Exclusive pH 7.2
A1BCC8	Pden_5112	Integral membrane sensor signal transduction histidine kinase	CytoplasmicMembrane	Exclusive pH 7.2
A1BCC9	Pden_5113	Response regulator receiver protein	Cytoplasmic	Exclusive pH 7.2
A1BCD0	Pden_5114	DNA polymerase III, epsilon subunit (EC 2.7.7.7)	Unknown	Exclusive pH 7.2
Q51699	Pden_2486	Protein NirI	Cytoplasmic/Membrane	Exclusive pH 7.2
A1AY04	Pden_0031	Uncharacterized protein	Unknown	Exclusive pH 7.2

A1AY05	Pden_0032	Molybdenum cofactor guanylyltransferase (MoCo guanylyltransferase) (EC 2.7.7.77) (GTP:molybdopterin guanylyltransferase) (Mo-MPT guanylyltransferase) (Molybdopterin guanylyltransferase) (Molybdopterin-guanine dinucleotide synthase) (MGD synthase)	Cytoplasmic	Exclusive pH 7.2
A1AY84	Pden_0111	Uncharacterized protein	Cytoplasmic/Membrane	Exclusive pH 7.2
A1AYI0	Pden_0210	Uncharacterized protein	Unknown	Exclusive pH 7.2
A1AYS1	Pden_0301	L-seryl-tRNA(Sec) selenium transferase (EC 2.9.1.1) (Selenocysteine synthase) (Sec synthase) (Selenocysteinyl-tRNA(Sec) synthase)	Cytoplasmic	Exclusive pH 7.2
A1AYW7	Pden_0347	Uncharacterized protein	Unknown	Exclusive pH 7.2
A1AZ18	Pden_0398	Exodeoxyribonuclease 7 small subunit (EC 3.1.11.6) (Exodeoxyribonuclease VII small subunit) (Exonuclease VII small subunit)	Cytoplasmic	Exclusive pH 7.2
A1AZC1	Pden_0503	Ribose ABC transporter ATP-binding protein / fructose ABC transporter ATP-binding protein / mannose ABC transporter ATP-binding protein	Cytoplasmic/Membrane	Exclusive pH 7.2
A1AZD5	Pden_0517	Uncharacterized protein	Unknown	Exclusive pH 7.2
A1AZE3	Pden_0525	Nucleoside ABC transporter membrane protein	Cytoplasmic/Membrane	Exclusive pH 7.2
A1AZJ7	Pden_0579	Ribosomal RNA large subunit methyltransferase J (EC 2.1.1.266) (23S rRNA (adenine(2030)-N6)-methyltransferase) (23S rRNA m6A2030 methyltransferase)	Cytoplasmic	Exclusive pH 7.2
A1AZR2	Pden_0644	Uncharacterized protein	CytoplasmicMembrane	Exclusive pH 7.2
A1AZV7	Pden_0689	tRNA(Ile)-lysidine synthase (EC 6.3.4.19) (tRNA(Ile)-2-lysyl-cytidine synthase) (tRNA(Ile)-lysidine synthetase)	Cytoplasmic	Exclusive pH 7.2
A1AZW6	Pden_0698	Multisubunit potassium/proton antiporter, PhaC subunit	Cytoplasmic/Membrane	Exclusive pH 7.2
A1B056	Pden_0788	Recombination protein MgsA	Cytoplasmic	Exclusive pH 7.2
A1B0B9	Pden_0851	Nicotinamidase (EC 3.5.1.19)	Cytoplasmic	Exclusive pH 7.2
A1B0C6	Pden_0858	Pyridoxine/pyridoxamine 5'-phosphate oxidase (EC 1.4.3.5) (PNP/PMP oxidase) (PNPOx) (Pyridoxal 5'-phosphate synthase)	Cytoplasmic	Exclusive pH 7.2
A1B0L4	Pden_0947	Glycine betaine/L-proline ABC transporter, ATPase subunit	Cytoplasmic/Membrane	Exclusive pH 7.2
A1B1A4	Pden_1193	Glycine betaine/L-proline ABC transporter, ATPase subunit	CytoplasmicMembrane	Exclusive pH 7.2
A1B1C6	Pden_1215	Activator of Hsp90 ATPase 1 family protein	Unknown	Exclusive pH 7.2
A1B1G0	Pden_1249	Binding-protein-dependent transport systems inner membrane component	Cytoplasmic/Membrane	Exclusive pH 7.2
A1B1J1	Pden_1280	Uncharacterized protein	Unknown	Exclusive pH 7.2
A1B1L1	Pden_1300	Uncharacterized protein	Cytoplasmic/Membrane	Exclusive pH 7.2

A1B1N8	Pden_1327	Binding-protein-dependent transport systems inner membrane component	Cytoplasmic/Membrane	Exclusive pH 7.2
A1B1N9	Pden_1328	Extracellular solute-binding protein, family 5	Periplasmic	Exclusive pH 7.2
A1B2A5	Pden_1549	Uncharacterized protein	Unknown	Exclusive pH 7.2
A1B2K7	Pden_1654	Lysine--tRNA ligase (EC 6.1.1.6)	Cytoplasmic	Exclusive pH 7.2
A1B2R8	Pden_1715	Amino acid/amide ABC transporter ATP-binding protein 2, HAAT family	Cytoplasmic	Exclusive pH 7.2
A1B2X4	Pden_1771	Integral membrane sensor signal transduction histidine kinase	Cytoplasmic/Membrane	Exclusive pH 7.2
A1B2Y2	Pden_1779	Uncharacterized conserved protein UCP032025	Unknown	Exclusive pH 7.2
A1B2Y9	Pden_1786	Response regulator receiver protein	Cytoplasmic	Exclusive pH 7.2
A1B316	Pden_1813	Pseudouridine-5'-phosphate glycosidase (PsiMP glycosidase) (EC 4.2.1.70)	Unknown	Exclusive pH 7.2
A1B353	Pden_1850	Putative transcriptional regulator, Crp/Fnr family	Cytoplasmic/Membrane	Exclusive pH 7.2
A1B3C3	Pden_1923	ATP phosphoribosyltransferase (EC 2.4.2.17)	Cytoplasmic	Exclusive pH 7.2
A1B3I3	Pden_1985	Putative pterin-4-alpha-carbinolamine dehydratase (PHS) (EC 4.2.1.96) (4-alpha-hydroxy-tetrahydropterin dehydratase) (Pterin carbinolamine dehydratase) (PCD)	Cytoplasmic	Exclusive pH 7.2
A1B3N0	Pden_2032	Ammonium transporter	/	Exclusive pH 7.2
A1B3T5	Pden_2087	Uncharacterized protein	Cytoplasmic	Exclusive pH 7.2
A1B3X5	Pden_2127	ABC transporter related protein	Cytoplasmic	Exclusive pH 7.2
A1B4B4	Pden_2266	Transcriptional regulator, AsnC family	Cytoplasmic	Exclusive pH 7.2
A1B4I2	Pden_2335	FAD dependent oxidoreductase	Unknown	Exclusive pH 7.2
A1B4I7	Pden_2340	Molybdopterin synthase subunit MoaE	Cytoplasmic	Exclusive pH 7.2
A1B4J8	Pden_2352	Histidine kinase	Cytoplasmic/Membrane	Exclusive pH 7.2
A1B4M4	Pden_2378	Putative RNA methylase	Cytoplasmic	Exclusive pH 7.2
A1B4T6	Pden_2443	Uncharacterized conserved protein UCP028301	Cytoplasmic	Exclusive pH 7.2
A1B504	Pden_2511	Uncharacterized protein	Cytoplasmic	Exclusive pH 7.2
A1B564	Pden_2571	Three-deoxy-D-manno-octulosonic-acid transferase domain protein	Unknown	Exclusive pH 7.2
A1B577	Pden_2584	SAM-dependent methyltransferase	Cytoplasmic	Exclusive pH 7.2
A1B581	Pden_2588	Transcriptional regulator, MarR family	Cytoplasmic	Exclusive pH 7.2
A1B5B0	Pden_2617	Iron-containing alcohol dehydrogenase	Cytoplasmic	Exclusive pH 7.2
A1B5P8	Pden_2758	Uncharacterized protein	Cytoplasmic	Exclusive pH 7.2
A1B5Q8	Pden_2768	HI0933 family protein	Cytoplasmic	Exclusive pH 7.2
A1B5R7	Pden_2777	5'-methylthioadenosine/S-adenosylhomocysteine nucleosidase	Cytoplasmic	Exclusive pH 7.2

A1B659	Pden_2919	Adenosylmethionine-8-amino-7-oxononanoate aminotransferase apoenzyme (EC 2.6.1.62)	Unknown	Exclusive pH 7.2
A1B669	Pden_2929	Transcriptional regulator, GntR family	Cytoplasmic	Exclusive pH 7.2
A1B671	Pden_2931	Gluconate transporter	Cytoplasmic/Membrane	Exclusive pH 7.2
A1B6A8	Pden_2968	Uncharacterized protein	Cytoplasmic	Exclusive pH 7.2
A1B6D2	Pden_2993	Pyrrolo-quinoline quinone	Periplasmic	Exclusive pH 7.2
A1B722	Pden_3235	Uncharacterized protein	Unknown	Exclusive pH 7.2
A1B735	Pden_3248	Regulatory protein, IclR	Unknown	Exclusive pH 7.2
A1B7G6	Pden_3387	TRAP dicarboxylate transporter, DctP subunit	Periplasmic	Exclusive pH 7.2
A1B7P5	Pden_3468	Putative transcriptional regulator, Crp/Fnr family	Cytoplasmic	Exclusive pH 7.2
A1B7Q6	Pden_3480	TRAP dicarboxylate transporter, DctP subunit	Periplasmic	Exclusive pH 7.2
A1B7Y0	Pden_3554	Uncharacterized protein	Cytoplasmic	Exclusive pH 7.2
A1B8A0	Pden_3677	Transcriptional regulator, GntR family	Cytoplasmic	Exclusive pH 7.2
A1B8A2	Pden_3679	Uncharacterized protein	Cytoplasmic	Exclusive pH 7.2
A1B8G8	Pden_3745	Uncharacterized protein	Unknown	Exclusive pH 7.2
A1B8P3	Pden_3821	Alpha/beta hydrolase fold protein	Cytoplasmic	Exclusive pH 7.2
A1B8W1	Pden_3889	Acriflavin resistance protein	Cytoplasmic/Membrane	Exclusive pH 7.2
A1B8Y6	Pden_3914	Uncharacterized protein	Cytoplasmic/Membrane	Exclusive pH 7.2
A1B8Z1	Pden_3919	D-3-hydroxyaspartate aldolase (EC 4.1.3.-)	Cytoplasmic	Exclusive pH 7.2
A1B8Z2	Pden_3920	Pyridoxal-5'-phosphate-dependent enzyme, beta subunit	Cytoplasmic	Exclusive pH 7.2
A1B905	Pden_3933	Transcriptional regulator, AraC family with amidase-like domain	Cytoplasmic	Exclusive pH 7.2
A1B998	Pden_4026	Uncharacterized protein	Cytoplasmic	Exclusive pH 7.2
A1B9I5	Pden_4115	Glyoxalase/bleomycin resistance protein/dioxygenase	Cytoplasmic	Exclusive pH 7.2
A1B9K2	Pden_4132	L-serine ammonia-lyase (EC 4.3.1.17)	Cytoplasmic	Exclusive pH 7.2
A1B9X7	Pden_4257	Molybdopterin dehydrogenase, FAD-binding protein	Cytoplasmic	Exclusive pH 7.2
A1B9Y0	Pden_4260	Guanine deaminase (EC 3.5.4.3)	Cytoplasmic	Exclusive pH 7.2
A1B9Y5	Pden_4265	Transcriptional regulator, GntR family	Cytoplasmic	Exclusive pH 7.2
A1B9Z2	Pden_4272	Amino acid/amide ABC transporter substrate-binding protein, HAAT family	Unknown	Exclusive pH 7.2
A1BAB9	Pden_4399	FAD linked oxidase domain protein	Cytoplasmic	Exclusive pH 7.2
A1BAD5	Pden_4415	Transcriptional regulator, XRE family	Cytoplasmic	Exclusive pH 7.2
A1BAF4	Pden_4434	Putative aminotransferase, class IV	Unknown	Exclusive pH 7.2

A1BAN2	Pden_4512	Formamidopyrimidine-DNA glycosylase (Fapy-DNA glycosylase) (EC 3.2.2.23) (DNA-(apurinic or apyrimidinic site) lyase MutM) (AP lyase MutM) (EC 4.2.99.18)	Cytoplasmic	Exclusive pH 7.2
A1BAS2	Pden_4553	Cyclic nucleotide-binding protein	Cytoplasmic	Exclusive pH 7.2
A1BAZ9	Pden_4630	Transcriptional regulator, TetR family	Unknown	Exclusive pH 7.2
A1BB76	Pden_4709	Uncharacterized protein	Unknown	Exclusive pH 7.2
A1BBC4	Pden_4757	Branched-chain alpha-keto acid dehydrogenase E1 component (EC 1.2.4.4)	Cytoplasmic	Exclusive pH 7.2
A1BBH8	Pden_4811	Beta-ketoadipyl CoA thiolase (EC 2.3.1.9)	Cytoplasmic	Exclusive pH 7.2
A1BBT1	Pden_4915	Transcriptional regulator, DeoR family	Cytoplasmic	Exclusive pH 7.2
A1BBW5	Pden_4949	Uncharacterized protein	Cytoplasmic	Exclusive pH 7.2
A1BC01	Pden_4985	Pyruvate dehydrogenase (Acetyl-transferring) (EC 1.2.4.1)	Cytoplasmic	Exclusive pH 7.2
A1BC12	Pden_4996	CoA-binding domain protein	Cytoplasmic	Exclusive pH 7.2
A1BC53	Pden_5037	ABC transporter related protein	Cytoplasmic	Exclusive pH 7.2
A1BC63	Pden_5047	Extracellular solute-binding protein, family 1	Periplasmic	Exclusive pH 7.2
A1BC82	Pden_5066	TRAP dicarboxylate transporter, DctP subunit	Periplasmic	Exclusive pH 7.2
P52220	Pden_1411	Heme exporter protein C (Cytochrome c-type biogenesis protein CcmC)	Cytoplasmic/Membrane	Exclusive pH 7.2
A1AXX6	Pden_0003	tRNA modification GTPase MnmE (EC 3.6.-.-)	Cytoplasmic	Exclusive pH 7.0
A1AYL3	Pden_0243	Succinate semialdehyde dehydrogenase (EC 1.2.1.16)	Cytoplasmic	Exclusive pH 7.0
A1AYM0	Pden_0250	Transcriptional regulator, RpiR family	Cytoplasmic	Exclusive pH 7.0
A1AZC6	Pden_0508	Uncharacterized protein	Cytoplasmic	Exclusive pH 7.0
A1AZG8	Pden_0550	Lytic transglycosylase, catalytic	Unknown	Exclusive pH 7.0
A1B1E6	Pden_1235	ABC transporter related protein	Cytoplasmic/Membrane	Exclusive pH 7.0
A1B1X1	Pden_1414	Uncharacterized protein	Cytoplasmic/Membrane	Exclusive pH 7.0
A1B290	Pden_1534	Filamentation induced by cAMP protein Fic	Cytoplasmic	Exclusive pH 7.0
A1B2T5	Pden_1732	Putative glutathione S-transferase	Cytoplasmic	Exclusive pH 7.0
A1B392	Pden_1891	Queuine tRNA-ribosyltransferase (EC 2.4.2.29) (Guanine insertion enzyme) (tRNA-guanine transglycosylase)	Cytoplasmic	Exclusive pH 7.0
A1B399	Pden_1898	HTH-type transcriptional regulator BetI	Unknown	Exclusive pH 7.0
A1B3F2	Pden_1954	SAM-dependent methyltransferase (EC 2.1.1.-)	Cytoplasmic	Exclusive pH 7.0
A1B3L7	Pden_2019	Uncharacterized protein	Cytoplasmic/Membrane	Exclusive pH 7.0
A1B4C5	Pden_2277	N-formylglutamate amidohydrolase	Cytoplasmic	Exclusive pH 7.0
A1B4I8	Pden_2341	Molybdopterin synthase sulfur carrier subunit	Unknown	Exclusive pH 7.0

A1B4X0	Pden_2477	N-acetylglucosamine-6-phosphate deacetylase (EC 3.5.1.25)	Cytoplasmic	Exclusive pH 7.0
A1B569	Pden_2576	ABC transporter related protein	Cytoplasmic/Membrane	Exclusive pH 7.0
A1B5P4	Pden_2754	Short-chain dehydrogenase/reductase SDR	Cytoplasmic	Exclusive pH 7.0
A1B5U1	Pden_2801	tRNA pseudouridine synthase B (EC 5.4.99.25) (tRNA pseudouridine(55) synthase) (Psi55 synthase) (tRNA pseudouridylate synthase) (tRNA-uridine isomerase)	Cytoplasmic	Exclusive pH 7.0
A1B613	Pden_2873	Endonuclease III (EC 4.2.99.18) (DNA-(apurinic or apyrimidinic site) lyase)	Cytoplasmic	Exclusive pH 7.0
A1B662	Pden_2922	Transcriptional regulator, GntR family	Cytoplasmic	Exclusive pH 7.0
A1B6P9	Pden_3112	Hydrogenase expression/formation protein HypE	Cytoplasmic	Exclusive pH 7.0
A1B6Z8	Pden_3211	Aldehyde dehydrogenase	Cytoplasmic	Exclusive pH 7.0
A1B702	Pden_3215	4-carboxymuconolactone decarboxylase (EC 4.1.1.44)	Cytoplasmic	Exclusive pH 7.0
A1B7Y1	Pden_3555	Uncharacterized protein	Cytoplasmic	Exclusive pH 7.0
A1B8R3	Pden_3841	Helicase domain protein	Cytoplasmic	Exclusive pH 7.0
A1B8T0	Pden_3858	ABC transporter related protein	Cytoplasmic/Membrane	Exclusive pH 7.0
A1B8T2	Pden_3860	NUDIX hydrolase	Cytoplasmic	Exclusive pH 7.0
A1B8T9	Pden_3867	DedA family protein	Cytoplasmic/Membrane	Exclusive pH 7.0
A1B8X4	Pden_3902	Uncharacterized protein	Unknown	Exclusive pH 7.0
A1B967	Pden_3995	Isoprenyl transferase (EC 2.5.1.-)	Cytoplasmic	Exclusive pH 7.0
A1B992	Pden_4020	ABC transporter related protein	Cytoplasmic/Membrane	Exclusive pH 7.0
A1B9L0	Pden_4140	Periplasmic solute binding protein	Periplasmic	Exclusive pH 7.0
A1BA45	Pden_4325	Two component transcriptional regulator, winged helix family	Cytoplasmic	Exclusive pH 7.0
A1BAE8	Pden_4428	1,4-alpha-glucan branching enzyme GlgB (EC 2.4.1.18) (1,4-alpha-D-glucan:1,4-alpha-D-glucan 6-glucosyl-transferase) (Alpha-(1->4)-glucan branching enzyme) (Glycogen branching enzyme) (BE)	Cytoplasmic	Exclusive pH 7.0
A1BAF3	Pden_4433	Transcriptional regulator, IclR family	Cytoplasmic	Exclusive pH 7.0
A1BAK9	Pden_4489	Cell division protein FtsQ	Cytoplasmic	Exclusive pH 7.0
A1BB27	Pden_4660	ABC transporter related protein	/	Exclusive pH 7.0
A1BBA6	Pden_4739	Alkylphosphonate utilization operon protein PhnA	Unknown	Exclusive pH 7.0
A1BC20	Pden_5004	Aliphatic sulfonates import ATP-binding protein SsuB 2 (EC 3.6.3.-)	Cytoplasmic/Membrane	Exclusive pH 7.0
A1BC85	Pden_5069	Cystathionine gamma-synthase (EC 2.5.1.48)	Cytoplasmic	Exclusive pH 7.0
A1BC94	Pden_5078	Transcriptional regulator, LacI family	Cytoplasmic	Exclusive pH 7.0
A1AZ39	Pden_0419	Ribonuclease 3 (EC 3.1.26.3) (Ribonuclease III) (RNase III)	Cytoplasmic	Exclusive pH 7.0

A1AZ62	Pden_0442	Uncharacterized protein	Unknown	Exclusive pH 7.0
A1AZG7	Pden_0549	GCN5-related N-acetyltransferase	Cytoplasmic	Exclusive pH 7.0
A1AZJ1	Pden_0573	Uncharacterized protein	Cytoplasmic	Exclusive pH 7.0
A1AZS4	Pden_0656	Phosphoadenylyl-sulfate reductase (thioredoxin) (EC 1.8.4.8)	Cytoplasmic	Exclusive pH 7.0
A1AZX3	Pden_0705	Lytic transglycosylase, catalytic	Periplasmic	Exclusive pH 7.0
A1AZY3	Pden_0715	Urocanate hydratase (Urocanase) (EC 4.2.1.49) (Imidazolonepropionate hydrolase)	Cytoplasmic	Exclusive pH 7.0
A1B1R2	Pden_1354	Uncharacterized protein	Cytoplasmic	Exclusive pH 7.0
A1B1V5	Pden_1398	Gamma-glutamylputrescine oxidase	Cytoplasmic	Exclusive pH 7.0
A1B1W4	Pden_1407	Uncharacterized protein	Cytoplasmic/Membrane	Exclusive pH 7.0
A1B394	Pden_1893	Methionine-R-sulfoxide reductase (EC 1.8.4.11)	Cytoplasmic	Exclusive pH 7.0
A1B3I1	Pden_1983	Uncharacterized protein	Cytoplasmic	Exclusive pH 7.0
A1B477	Pden_2229	Type III pantothenate kinase (EC 2.7.1.33) (PanK-III) (Pantothenic acid kinase)	Unknown	Exclusive pH 7.0
A1B4Q9	Pden_2415	Transcriptional regulator, XRE family	Unknown	Exclusive pH 7.0
A1B558	Pden_2565	Putative esterase	Unknown	Exclusive pH 7.0
A1B5A4	Pden_2611	Transcriptional regulator, XRE family	Unknown	Exclusive pH 7.0
A1B5F4	Pden_2661	Beta-lactamase	Cytoplasmic/Membrane	Exclusive pH 7.0
A1B5T4	Pden_2794	UvrD-like DNA helicase, C-terminal domain	Cytoplasmic	Exclusive pH 7.0
A1B5Z8	Pden_2858	Formate dehydrogenase gamma subunit (EC 1.2.1.2)	Cytoplasmic	Exclusive pH 7.0
A1B681	Pden_2941	Efflux transporter, RND family, MFP subunit	Cytoplasmic/Membrane	Exclusive pH 7.0
A1B682	Pden_2942	Transporter, hydrophobe/amphiphile efflux-1 (HAE1) family	Cytoplasmic/Membrane	Exclusive pH 7.0
A1B6M0	Pden_3083	Putative outer membrane protein	Outer Membrane	Exclusive pH 7.0
A1B717	Pden_3230	AMP-dependent synthetase and ligase	Cytoplasmic	Exclusive pH 7.0
A1B841	Pden_3618	Glutathione S-transferase, C-terminal domain	Cytoplasmic	Exclusive pH 7.0
A1B845	Pden_3622	GCN5-related N-acetyltransferase	Cytoplasmic	Exclusive pH 7.0
A1B875	Pden_3652	ABC transporter related protein	Cytoplasmic	Exclusive pH 7.0
A1B8K6	Pden_3784	Cytidylate kinase (CK) (EC 2.7.4.25) (Cytidine monophosphate kinase) (CMP kinase)	Cytoplasmic	Exclusive pH 7.0
A1B8S8	Pden_3856	Substrate-binding region of ABC-type glycine betaine transport system	Periplasmic	Exclusive pH 7.0
A1B914	Pden_3942	Ubiquinone biosynthesis hydroxylase, UbiH/UbiF/VisC/COQ6 family	Cytoplasmic	Exclusive pH 7.0
A1B961	Pden_3989	Ppx/GppA phosphatase	Cytoplasmic	Exclusive pH 7.0
A1B9A3	Pden_4031	Uncharacterized protein	Cytoplasmic	Exclusive pH 7.0

A1B9J6	Pden_4126	TrkA-N domain protein	Cytoplasmic/Membrane	Exclusive pH 7.0
A1B9K8	Pden_4138	Zinc import ATP-binding protein ZnuC (EC 3.6.3.-)	Cytoplasmic/Membrane	Exclusive pH 7.0
A1B9S0	Pden_4200	ABC transporter related protein	Cytoplasmic/Membrane	Exclusive pH 7.0
A1BA17	Pden_4297	GTP cyclohydrolase FolE2 (EC 3.5.4.16)	Cytoplasmic	Exclusive pH 7.0
A1BA44	Pden_4324	OmpA/MotB domain protein	Unknown	Exclusive pH 7.0
A1BA62	Pden_4342	Sulfate ABC transporter, inner membrane subunit CysW	Cytoplasmic/Membrane	Exclusive pH 7.0
A1BAS8	Pden_4559	Uncharacterized protein	Unknown	Exclusive pH 7.0
A1BAX3	Pden_4604	Transcriptional regulator, IclR family	Cytoplasmic	Exclusive pH 7.0
A1BC26	Pden_5010	ABC transporter related protein	Cytoplasmic/Membrane	Exclusive pH 7.0
A1BC49	Pden_5033	BioY protein	Cytoplasmic/Membrane	Exclusive pH 7.0
A1BCE3	Pden_5127	Transcriptional regulator, Fis family	Unknown	Exclusive pH 7.0
P52221	Pden_1410	Heme exporter protein D (Cytochrome c-type biogenesis protein CcmD)	Unknown	Exclusive pH 7.0

[1]Protein annotated from UniProt (UP000000361). [2]Genes annotated from GeneBank (T00440). [3]Subcellular location according with PSOTb v3.0.2.[4]Fold change calculated as the ratio protein expression at pH 7.2/pH 7.0 protein expression at pH 7.2 and at pH 7.0 (positive values indicate proteins over-represented at pH 7.2 and negative values correspond to proteins over-represented at pH 7.0). Proteins showing an absolute value of fold change >100 were considered exclusive at the indicated pH.

Table S3. Differential analysis of the *P. denitrificans* proteome by LC-MS/MS at pH 7.2 *versus* pH 7.5 (pH 7.2 was used as reference).

Protein ID[1]	Gene ID[2]	Name	Location[3]	FC[4]
A1B9J2	Pden_4122	TRAP dicarboxylate transporter, DctP subunit	Periplasmic	7882.29
A1BAC4	Pden_4404	Uncharacterized protein UPF0065	Unknown	544.19
A1AY25	Pden_0052	Amino acid/amide ABC transporter substrate-binding protein, HAAT family	Unknown	255.44
A1AYS8	Pden_0308	Uncharacterized protein UPF0065	Periplasmic	90.22
A1B862	Pden_3639	3-methylcrotonoyl-CoA carboxylase, alpha subunit (EC 6.4.1.4)	Cytoplasmic	58.86
A1BAC1	Pden_4401	Uncharacterized protein UPF0065	Unknown	41.11
A1AXZ3	Pden_0020	Pyrrolo-quinoline quinone	Periplasmic	40.98
A1B646	Pden_2906	Acyl-CoA dehydrogenase domain protein	Cytoplasmic	33.02
A1AY01	Pden_0028	Uncharacterized protein	Unknown	29.10
A1B856	Pden_3633	Isovaleryl-CoA dehydrogenase (EC 1.3.8.4)	Cytoplasmic	24.79
A1B647	Pden_2907	Acetyl-CoA acetyltransferase (EC 2.3.1.9)	Cytoplasmic	20.18
A1B9T1	Pden_4211	SSS sodium solute transporter superfamily	Cytoplasmic/Membrane	18.55
A1B648	Pden_2908	Short-chain dehydrogenase/reductase SDR	Unknown	15.29
A1B2S0	Pden_1717	Amino acid/amide ABC transporter substrate-binding protein, HAAT family	Unknown	13.80
A1BBI4	Pden_4817	TRAP dicarboxylate transporter, DctP subunit	Periplasmic	11.61
A1B9T3	Pden_4213	Acetyl-coenzyme A synthetase (AcCoA synthetase) (Acs) (EC 6.2.1.1) (Acetate--CoA ligase) (Acyl-activating enzyme)	Cytoplasmic	11.50
A1BAR9	Pden_4550	Acetyl-coenzyme A synthetase (AcCoA synthetase) (Acs) (EC 6.2.1.1) (Acetate--CoA ligase) (Acyl-activating enzyme)	Cytoplasmic	11.50
A1B5F6	Pden_2663	Acetyl-CoA acetyltransferase (EC 2.3.1.9)	Cytoplasmic	10.69
A1AXY8	Pden_0015	Glutathione-dependent formaldehyde-activating enzyme (EC 4.4.1.22) (S-(hydroxymethyl)glutathione synthase)	Cytoplasmic	10.31
A1B8U5	Pden_3873	Crotonyl-CoA reductase	Cytoplasmic	9.67
A1B4X6	Pden_2483	Nitric oxide reductase, NorB subunit apoprotein	Cytoplasmic/Membrane	9.64
A1B8B1	Pden_3688	Propionyl-CoA carboxylase carboxyltransferase subunit	Cytoplasmic	8.94
A1B3K3	Pden_2005	AMP-dependent synthetase and ligase	Cytoplasmic	8.93
A1B8A7	Pden_3684	Biotin carboxyl carrier protein / biotin carboxylase (EC 6.3.4.14)	Cytoplasmic	8.05
A1B5Y0	Pden_2840	Acyl-CoA dehydrogenase (EC 1.3.8.-)	Cytoplasmic	6.99

A1B8A4	Pden_3681	Methylmalonyl-CoA mutase (EC 5.4.99.2)	Cytoplasmic	6.93
A1B7I1	Pden_3404	Carbohydrate ABC transporter substrate-binding protein, CUT1 family	Periplasmic	6.92
A1B810	Pden_3586	Transglutaminase domain protein	Unknown	6.89
A1B4W6	Pden_2473	Uncharacterized protein	Unknown	6.80
A1BAK2	Pden_4482	3-hydroxyisobutyrate dehydrogenase (HIBADH) (EC 1.1.1.31)	Cytoplasmic	6.25
A1B9P3	Pden_4173	TonB-dependent receptor	Outer Membrane	6.07
A1AZS8	Pden_0660	Gamma-glutamyltransferase 1, Threonine peptidase, MEROPS family T03 (EC 2.3.2.2)	Periplasmic	6.07
A1B2N7	Pden_1684	Monosaccharide ABC transporter substrate-binding protein, CUT2 family	Periplasmic	5.98
A1AZY9	Pden_0721	Patatin	Cytoplasmic	5.88
A1AZC3	Pden_0505	Mannose-binding protein / fructose-binding protein / ribose-binding protein	Periplasmic	5.71
A1BC00	Pden_4984	Transketolase, central region	Cytoplasmic	5.63
A1B9S9	Pden_4209	Phosphate butyryltransferase (EC 2.3.1.19)	Cytoplasmic	5.60
A1B608	Pden_2868	Acyl-CoA dehydrogenase domain protein	Cytoplasmic	5.51
A1AZK8	Pden_0590	Membrane protein involved in aromatic hydrocarbon degradation	Outer Membrane	5.44
A1B5Z6	Pden_2856	Formate dehydrogenase alpha subunit (EC 1.2.1.2)	Cytoplasmic	5.31
A1B0P5	Pden_0978	Uncharacterized protein	Unknown	5.04
A1B067	Pden_0799	Citrate (Pro-3S)-lyase (EC 4.1.3.6)	Cytoplasmic	4.96
A1B537	Pden_2544	Cytochrome c, class II	Periplasmic	4.95
A1B5W3	Pden_2823	2-nitropropane dioxygenase, NPD	Unknown	4.87
A1B2M4	Pden_1671	Extracellular solute-binding protein, family 5	Periplasmic	4.86
A1AZD0	Pden_0512	Sarcosine oxidase, beta subunit family	Cytoplasmic	4.81
A1B4W9	Pden_2476	Glutamine--fructose-6-phosphate transaminase (EC 2.6.1.16)	Cytoplasmic	4.72
A1B2B9	Pden_1563	TRAP dicarboxylate transporter-DctP subunit	Periplasmic	4.71
A1BCB9	Pden_5103	Poly-beta-hydroxybutyrate polymerase domain protein	Cytoplasmic	4.69
A1B4W5	Pden_2472	Carbohydrate ABC transporter substrate-binding protein, CUT1 family	Periplasmic	4.55
A1BBI7	Pden_4820	AMP-dependent synthetase and ligase	Cytoplasmic	4.53
A1B2T8	Pden_1735	Dyp-type peroxidase family	Periplasmic	4.51
A1B9R0	Pden_4190	Carbohydrate ABC transporter substrate-binding protein, CUT1 family	Periplasmic	4.49
A1B078	Pden_0810	Extracellular solute-binding protein, family 5	Periplasmic	4.36
A1BA29	Pden_4309	Acetyl-CoA hydrolase (EC 3.1.2.1)	Cytoplasmic	4.19
A1B6E6	Pden_3007	TonB-dependent siderophore receptor	Outer Membrane	4.18
A1B0M5	Pden_0958	Poly(R)-hydroxyalkanoic acid synthase, class I	Cytoplasmic	4.05

A1B7P7	Pden_3470	Uncharacterized protein	Unknown	3.98
A1BAR5	Pden_4546	TRAP dicarboxylate transporter-DctP subunit	Unknown	3.97
A1B595	Pden_2602	Branched chain amino acid aminotransferase apoenzyme (EC 2.6.1.42)	Cytoplasmic	3.94
A1B473	Pden_2225	Propionyl-CoA synthetase (EC 6.2.1.17)	Cytoplasmic	3.84
A1B9C1	Pden_4049	Uncharacterized protein	Cytoplasmic	3.84
A1B0L7	Pden_0950	Fervidolysin, Serine peptidase, MEROPS family S08A	Cytoplasmic	3.82
A1B9S2	Pden_4202	Hemin-degrading family protein	Cytoplasmic	3.72
A1BAH4	Pden_4454	Putative nitrate transport protein	Cytoplasmic/Membrane	3.71
A1AYG3	Pden_0191	Glyoxalase/bleomycin resistance protein/dioxygenase	Cytoplasmic	3.63
A1B2T9	Pden_1736	Uncharacterized protein	Periplasmic	3.63
A1B6E4	Pden_3005	Uncharacterized protein	Unknown	3.61
A1AYH6	Pden_0206	Short-chain dehydrogenase/reductase SDR	Cytoplasmic	3.60
A1B2S2	Pden_1719	AMP-dependent synthetase and ligase	Cytoplasmic	3.54
A1BAE7	Pden_4427	Glucose-1-phosphate adenylyltransferase (EC 2.7.7.27) (ADP-glucose pyrophosphorylase) (ADPGlc PPase) (ADP-glucose synthase)	Cytoplasmic	3.53
A1BB77	Pden_4710	Glucose-methanol-choline oxidoreductase	Cytoplasmic	3.50
A1B2N6	Pden_1683	Putative ribose/galactose/methyl galactoside import ATP-binding protein (EC 3.6.3.17)	Cytoplasmic/Membrane	3.47
A1AZE6	Pden_0528	Nucleoside-binding protein	Unknown	3.44
A1B097	Pden_0829	Extracellular solute-binding protein, family 1	Unknown	3.42
A1B951	Pden_3979	Enoyl-CoA hydratase (EC 4.2.1.17)	Cytoplasmic	3.41
A1BCC6	Pden_5110	Putative signal-transduction protein with CBS domains	Cytoplasmic	3.40
A1B051	Pden_0783	30S ribosomal protein S11	Cytoplasmic	3.36
A1B415	Pden_2167	Glutamate-ammonia-ligase adenylyltransferase (EC 2.7.7.42)	Cytoplasmic	3.29
A1AYK9	Pden_0239	Extracellular solute-binding protein, family 5	Periplasmic	3.22
A1B2N5	Pden_1682	Monosaccharide ABC transporter membrane protein, CUT2 family	Cytoplasmic/Membrane	3.17
A1B076	Pden_0808	Uncharacterized protein	Unknown	3.15
A1B610	Pden_2870	Acetyl-CoA acetyltransferase (EC 2.3.1.9)	Cytoplasmic	3.14
A1B347	Pden_1844	4Fe-4S ferredoxin, iron-sulfur binding domain protein	Cytoplasmic/Membrane	3.12
A1BB94	Pden_4727	Glucose sorbosone dehydrogenase	Unknown	3.10
A1B607	Pden_2867	Transcriptional regulator, MerR family	Cytoplasmic	3.09
A1AZ54	Pden_0434	Acyl-CoA dehydrogenase domain protein	Cytoplasmic	3.06
A1B4F5	Pden_2308	Ribokinase (RK) (EC 2.7.1.15)	Cytoplasmic	3.06
A1B4Z6	Pden_2503	Asparaginase (EC 3.5.1.1)	Cytoplasmic	3.04

A1B3D7	Pden_1938	Cytochrome c oxidase subunit 1 (EC 1.9.3.1)	Cytoplasmic/Membrane	3.03
A1B2N4	Pden_1681	Monosaccharide ABC transporter substrate-binding protein, CUT2 family	Periplasmic	3.02
A1B1W2	Pden_1405	Uncharacterized protein	Cytoplasmic/Membrane	2.99
A1B2M1	Pden_1668	ABC transporter related protein	Cytoplasmic/Membrane	2.98
A1B357	Pden_1854	Uncharacterized protein	Unknown	2.90
A1BBC7	Pden_4760	Dihydrolipoyl dehydrogenase (EC 1.8.1.4)	Cytoplasmic	2.86
A1B2M5	Pden_1672	5-deoxyglucuronate isomerase (EC 5.3.1.-)	Cytoplasmic	2.84
A1B122	Pden_1108	AMP-dependent synthetase and ligase	Cytoplasmic	2.83
A1B5U3	Pden_2803	Beta-lactamase domain protein	Unknown	2.83
A1BAG7	Pden_4447	Extracellular solute-binding protein, family 1	Periplasmic	2.82
A1B611	Pden_2871	3-hydroxyacyl-CoA dehydrogenase (EC 1.1.1.35)	Cytoplasmic	2.82
A1B8C8	Pden_3705	Amino acid ABC transporter substrate-binding protein, PAAT family	Periplasmic	2.80
A1AYQ1	Pden_0281	Hydroxyectoine-binding protein / ectoine-binding protein	Periplasmic	2.69
A1B0C0	Pden_0852	Nicotinate phosphoribosyltransferase (NAPRTase) (EC 6.3.4.21)	Cytoplasmic	2.67
A1B487	Pden_2239	NADH-quinone oxidoreductase subunit H (EC 1.6.5.11) (NADH dehydrogenase I subunit 8) (NADH dehydrogenase I subunit H) (NADH-quinone oxidoreductase subunit 8) (NQO8) (NDH-1 subunit 8) (NDH-1 subunit H)	Cytoplasmic/Membrane	2.66
A1B2T7	Pden_1734	Uncharacterized protein	Periplasmic	2.63
A1AZI4	Pden_0566	L-erythro-3-methylmalyl-CoA dehydratase (EC 4.2.1.-)	Cytoplasmic	2.62
A1B376	Pden_1875	BolA family protein	Unknown	2.62
A1B2W6	Pden_1763	Nucleoside ABC transporter ATP-binding protein	Cytoplasmic/Membrane	2.61
A1B5R1	Pden_2771	5'-nucleotidase, lipoprotein e(P4) family	Outer Membrane	2.59
A1B9S1	Pden_4201	TonB-dependent heme/hemoglobin receptor family protein	Outer Membrane	2.55
A1AZD3	Pden_0515	Sarcosine oxidase, alpha subunit family	Cytoplasmic	2.54
A1AZQ1	Pden_0633	Thiamine pyrophosphate enzyme TPP binding domain protein	Cytoplasmic	2.54
A1B4B7	Pden_2269	Alcohol dehydrogenase, zinc-binding domain protein	Cytoplasmic	2.47
A1B4W2	Pden_2469	Carbohydrate ABC transporter ATP-binding protein, CUT1 family	Cytoplasmic/Membrane	2.47
A1B4A5	Pden_2257	Succinate semialdehyde dehydrogenase (EC 1.2.1.16)	Cytoplasmic	2.45
A1B878	Pden_3655	Glycosyl transferase, group 1	Cytoplasmic	2.43
A1B092	Pden_0824	Uncharacterized protein	Unknown	2.42

A1B4D2	Pden_2285	Substrate-binding region of ABC-type glycine betaine transport system	Periplasmic	2.41
A1AZ52	Pden_0432	Aa3 type cytochrome c oxidase subunit IV	Cytoplasmic/Membrane	2.40
A1B880	Pden_3657	Uncharacterized protein	Unknown	2.39
A1B8X0	Pden_3898	Aldehyde dehydrogenase (NAD(+)) (EC 1.2.1.3)	Cytoplasmic	2.39
A1B428	Pden_2180	GCN5-related N-acetyltransferase	Unknown	2.39
A1BB21	Pden_4654	HAD superfamily (Subfamily IIIB) phosphatase, TIGR01672 (EC 3.1.3.2)	Unknown	2.36
A1B5G4	Pden_2671	Amidase	Cytoplasmic	2.34
A1AZM6	Pden_0608	Extracellular solute-binding protein, family 5	Periplasmic	2.32
A1BBC6	Pden_4759	Dihydrolipoamide acetyltransferase component of pyruvate dehydrogenase complex (EC 2.3.1.-)	Cytoplasmic	2.31
A1BAW1	Pden_4592	Alkyl hydroperoxide reductase AhpD (EC 1.11.1.15)	Unknown	2.30
A1B1B8	Pden_1207	Urease accessory protein UreE	Cytoplasmic	2.29
A1B3B6	Pden_1916	Peptidyl-prolyl cis-trans isomerase (PPIase) (EC 5.2.1.8)	Unknown	2.27
A1BA20	Pden_4300	Uncharacterized protein	Unknown	2.27
A1AZI1	Pden_0563	Citryl-CoA lyase (EC 4.1.3.34)	Cytoplasmic	2.26
A1B2C5	Pden_1569	Extracellular solute-binding protein, family 5	Periplasmic	2.26
A1B470	Pden_2222	Thymidine phosphorylase (EC 2.4.2.4)	Unknown	2.24
A1B3V3	Pden_2105	Transcriptional regulator, GntR family	Cytoplasmic	2.24
A1B1N5	Pden_1324	Oligopeptide/dipeptide ABC transporter, ATPase subunit	Cytoplasmic/Membrane	2.23
A1B3P5	Pden_2047	Arginase (EC 3.5.3.1)	Unknown	2.22
A1B1L8	Pden_1307	Uncharacterized protein	Unknown	2.21
A1B4L0	Pden_2364	Alpha-2-macroglobulin domain protein	Unknown	2.20
A1B2N1	Pden_1678	Inositol 2-dehydrogenase (EC 1.1.1.18) (Myo-inositol 2-dehydrogenase) (MI 2-dehydrogenase)	Unknown	2.20
A1B956	Pden_3984	Aminotransferase (EC 2.6.1.-)	Cytoplasmic	2.19
A1B9B4	Pden_4042	Uncharacterized protein	Cytoplasmic	2.19
A1B457	Pden_2209	Transcriptional regulator, AsnC family	Cytoplasmic	2.18
A1B4N1	Pden_2386	2,3-dihydroxybenzoate-AMP ligase	Cytoplasmic	2.18
A1B3M5	Pden_2027	3-oxoacyl-[acyl-carrier-protein] reductase (EC 1.1.1.100)	Cytoplasmic	2.18
A1B574	Pden_2581	ErfK/YbiS/YcfS/YnhG family protein	Unknown	2.17
A1B4X1	Pden_2478	Putative transcriptional regulator, Crp/Fnr family	Cytoplasmic	2.17
A1B1G6	Pden_1255	Short-chain dehydrogenase/reductase SDR	Cytoplasmic	2.16
A1B0L2	Pden_0945	Substrate-binding region of ABC-type glycine betaine transport system	Periplasmic	2.16
A1BB61	Pden_4694	FAD-dependent pyridine nucleotide-disulfide oxidoreductase	Periplasmic	2.16
A1B484	Pden_2236	Carboxymuconolactone decarboxylase	Unknown	2.16
A1B2M9	Pden_1676	5-dehydro-2-deoxygluconokinase (EC 2.7.1.92)	Cytoplasmic	2.15

A1B284	Pden_1528	Uncharacterized protein	Cytoplasmic	2.15
A1B0J3	Pden_0926	Uncharacterized protein	Unknown	2.14
A1BBI9	Pden_4822	Short-chain dehydrogenase/reductase SDR	Cytoplasmic	2.13
A1B1W1	Pden_1404	OmpA/MotB domain protein	Cytoplasmic/Membrane	2.12
A1AZZ5	Pden_0727	Outer membrane transport energization protein ExbB	Cytoplasmic/Membrane	2.12
A1B1F9	Pden_1248	Extracellular solute-binding protein, family 5	Periplasmic	2.11
A1B3T8	Pden_2090	Uncharacterized protein	Cytoplasmic	2.08
A1B3I2	Pden_1984	Dihydroxy-acid dehydratase (DAD) (EC 4.2.1.9)	Cytoplasmic	2.07
A1B2M0	Pden_1667	ABC transporter related protein	Cytoplasmic/Membrane	2.06
A1B446	Pden_2198	Pyrroline-5-carboxylate reductase (P5C reductase) (P5CR) (EC 1.5.1.2) (PCA reductase)	Cytoplasmic	2.06
A1B688	Pden_2948	Antifreeze protein, type I	Cytoplasmic	2.04
A1B8U9	Pden_3877	Alkyl hydroperoxide reductase AhpD (EC 1.11.1.15)	Unknown	2.03
A1B4I5	Pden_2338	4-aminobutyrate aminotransferase apoenzyme (EC 2.6.1.19)	Cytoplasmic	2.03
A1B459	Pden_2211	Laccase domain protein	Unknown	2.03
A1B9W4	Pden_4244	Asparaginase (EC 3.5.1.1)	Unknown	2.03
A1B0M4	Pden_0957	Polyhydroxyalkanoate depolymerase, intracellular	Cytoplasmic	2.02
A1AYV0	Pden_0330	NADPH-dependent FMN reductase	Cytoplasmic	2.02
A1B352	Pden_1849	UspA domain protein	Cytoplasmic	2.02
A1B2B3	Pden_1557	Riboflavin synthase, alpha subunit	Cytoplasmic	2.02
A1AYF2	Pden_0180	Aldehyde dehydrogenase	Cytoplasmic	2.01
A1B994	Pden_4022	Redoxin domain protein	Unknown	2.00
A1B5P9	Pden_2759	Polyribonucleotide nucleotidyltransferase (EC 2.7.7.8) (Polynucleotide phosphorylase) (PNPase)	Cytoplasmic	-2.00
A1B346	Pden_1843	FixH family protein	Cytoplasmic/Membrane	-2.01
A1B030	Pden_0762	50S ribosomal protein L2	Cytoplasmic	-2.02
A1B585	Pden_2592	Transcription termination/antitermination protein NusA	Cytoplasmic	-2.03
A1BA23	Pden_4303	30S ribosomal protein S4	Cytoplasmic	-2.03
A1B0I9	Pden_0922	Peptide chain release factor 3 (RF-3)	Cytoplasmic	-2.04
A1B027	Pden_0759	50S ribosomal protein L3	Cytoplasmic	-2.04
A1AZN6	Pden_0618	Bifunctional protein GlmU [Includes: UDP-N-acetylglucosamine pyrophosphorylase (EC 2.7.7.23) (N-acetylglucosamine-1-phosphate uridyltransferase); Glucosamine-1-phosphate N-acetyltransferase (EC 2.3.1.157)]	Cytoplasmic	-2.04

A1BAG9	Pden_4449	Assimilatory nitrate reductase (NADH) alpha subunit apoprotein (EC 1.7.1.1)	Cytoplasmic	-2.05
A1BAV5	Pden_4586	10 kDa chaperonin (GroES protein) (Protein Cpn10)	Cytoplasmic	-2.07
A1B8R2	Pden_3840	Uncharacterized protein	Cytoplasmic	-2.08
A1B8V0	Pden_3878	Signal recognition particle protein (Fifty-four homolog)	Cytoplasmic/Membrane	-2.09
A1B832	Pden_3609	Lon protease (EC 3.4.21.53) (ATP-dependent protease La)	Cytoplasmic	-2.10
A1B0L8	Pden_0951	Ribonucleoside-diphosphate reductase, adenosylcobalamin-dependent	Cytoplasmic	-2.10
A1B029	Pden_0761	50S ribosomal protein L23	Cytoplasmic	-2.11
A1B041	Pden_0773	30S ribosomal protein S8	Cytoplasmic	-2.11
A1B8E9	Pden_3726	30S ribosomal protein S2	Cytoplasmic	-2.11
A1B472	Pden_2224	Malate dehydrogenase (Oxaloacetate-decarboxylating) (NADP(+))., Phosphate acetyltransferase (EC 1.1.1.40) (EC 2.3.1.8)	Cytoplasmic	-2.12
A1B2U3	Pden_1740	Uncharacterized protein	Cytoplasmic	-2.12
A1B4C1	Pden_2273	50S ribosomal protein L20	Cytoplasmic	-2.12
A1B612	Pden_2872	PfkB domain protein	Cytoplasmic	-2.12
A1B043	Pden_0775	50S ribosomal protein L18	Cytoplasmic	-2.13
A1B684	Pden_2944	Phosphomethylpyrimidine synthase (EC 4.1.99.17) (Hydroxymethylpyrimidine phosphate synthase) (HMP-P synthase) (HMP-phosphate synthase) (HMPP synthase) (Thiamine biosynthesis protein ThiC)	Cytoplasmic	-2.13
A1B339	Pden_1836	DNA topoisomerase 4 subunit B (EC 5.99.1.3) (Topoisomerase IV subunit B)	Cytoplasmic	-2.14
A1AZ25	Pden_0405	Histone family protein DNA-binding protein	Cytoplasmic	-2.16
A1AZP4	Pden_0626	OmpA/MotB domain protein	Cytoplasmic/Membrane	-2.18
A1B052	Pden_0784	DNA-directed RNA polymerase subunit alpha (RNAP subunit alpha) (EC 2.7.7.6) (RNA polymerase subunit alpha) (Transcriptase subunit alpha)	Cytoplasmic	-2.18
A1B371	Pden_1868	Beta-N-acetylhexosaminidase (EC 3.2.1.52)	Cytoplasmic	-2.20
A1B2A7	Pden_1551	Uncharacterized protein	Outer Membrane	-2.21
A1BAH1	Pden_4451	Assimilatory nitrite reductase (NAD(P)H) small subunit (EC 1.7.1.4)	Unknown	-2.21
A1B584	Pden_2591	Ribosome maturation factor RimP	Cytoplasmic	-2.21
A1B8V6	Pden_3884	50S ribosomal protein L19	Cytoplasmic	-2.23
A1BAH8	Pden_4458	RNA binding S1 domain protein	Cytoplasmic	-2.24
A1B380	Pden_1879	Carbamoyl-phosphate synthase large chain (EC 6.3.5.5) (Carbamoyl-phosphate synthetase ammonia chain)	Unknown	-2.24
A1B8W3	Pden_3891	Transketolase, central region	Cytoplasmic	-2.24
A1B9H4	Pden_4102	50S ribosomal protein L27	Cytoplasmic	-2.25

A1B3D4	Pden_1935	Methionine synthase (B12-dependent) (EC 2.1.1.13)	Cytoplasmic	-2.28
A1BAF0	Pden_4430	Nicotinate-nucleotide pyrophosphorylase (Carboxylating) (EC 2.4.2.19)	Cytoplasmic	-2.29
A1B5N3	Pden_2743	Elongation factor P (EF-P)	Cytoplasmic	-2.31
A1B5R5	Pden_2775	Aspartate-semialdehyde dehydrogenase (ASA dehydrogenase) (ASADH) (EC 1.2.1.11) (Aspartate-beta-semialdehyde dehydrogenase)	Cytoplasmic	-2.32
Q51687	Pden_1409	Histidinol-phosphate aminotransferase (EC 2.6.1.9) (Imidazole acetol-phosphate transaminase)	Cytoplasmic	-2.32
A1B9C7	Pden_4055	Glutamate 5-kinase (EC 2.7.2.11) (Gamma-glutamyl kinase) (GK)	Cytoplasmic	-2.32
A1B032	Pden_0764	50S ribosomal protein L22	Cytoplasmic	-2.33
A1AZ38	Pden_0418	GTPase Era	Cytoplasmic/Membrane	-2.33
A1B362	Pden_1859	50S ribosomal protein L32	Cytoplasmic	-2.34
A1BA52	Pden_4332	Chorismate synthase (CS) (EC 4.2.3.5) (5-enolpyruvylshikimate-3-phosphate phospholyase)	Cytoplasmic	-2.35
A1B328	Pden_1825	Transcriptional regulator, CarD family	Cytoplasmic	-2.37
A1AZH7	Pden_0559	Succinate--CoA ligase [ADP-forming] subunit beta (EC 6.2.1.5) (Succinyl-CoA synthetase subunit beta) (SCS-beta)	Cytoplasmic	-2.37
A1B4X4	Pden_2481	von Willebrand factor, type A	Unknown	-2.37
A1B2U9	Pden_1746	Nucleoside diphosphate kinase (NDK) (NDP kinase) (EC 2.7.4.6) (Nucleoside-2-P kinase)	Extracellular	-2.39
A1B028	Pden_0760	50S ribosomal protein L4	Cytoplasmic	-2.40
A1B026	Pden_0758	30S ribosomal protein S10	Cytoplasmic	-2.42
A1B8A8	Pden_3685	Uncharacterized protein	Unknown	-2.42
A1B869	Pden_3646	Heat shock protein Hsp20	Cytoplasmic	-2.44
A1AZA8	Pden_0490	Glutamate synthase (NADPH) small subunit (EC 1.4.1.13)	Cytoplasmic	-2.44
A1B9L2	Pden_4142	Pseudouridine synthase (EC 5.4.99.-)	Cytoplasmic	-2.45
A1BAG3	Pden_4443	Electron transport protein SCO1/SenC	Unknown	-2.47
A1AY33	Pden_0060	Orotidine 5'-phosphate decarboxylase (EC 4.1.1.23) (OMP decarboxylase) (OMPDCase) (OMPdecase)	Cytoplasmic	-2.47
A1B4Q6	Pden_2412	Type I secretion membrane fusion protein, HlyD family	Cytoplasmic/Membrane	-2.48
A1B080	Pden_0812	Phosphoserine phosphatase (EC 3.1.3.3)	Cytoplasmic	-2.49
A1B318	Pden_1815	Peptide chain release factor 2 (RF-2)	Cytoplasmic	-2.49
A1B9E9	Pden_4077	50S ribosomal protein L25 (General stress protein CTC)	Cytoplasmic	-2.51
A1B0F4	Pden_0887	Trigger factor (TF) (EC 5.2.1.8) (PPIase)	Cytoplasmic	-2.56
A1BC29	Pden_5013	Alkanesulfonate monooxygenase (EC 1.14.14.5)	Cytoplasmic	-2.56
A1B035	Pden_0767	50S ribosomal protein L29	Cytoplasmic	-2.59
A1B0E5	Pden_0878	MOSC domain containing protein	Cytoplasmic	-2.60

A1B8K8	Pden_3786	30S ribosomal protein S1	Cytoplasmic	-2.61
A1AZS6	Pden_0658	Ferredoxin--NADP(+) reductase (EC 1.18.1.2)	Cytoplasmic	-2.61
A1B047	Pden_0779	50S ribosomal protein L15	Cytoplasmic	-2.67
A1BC28	Pden_5012	ABC transporter substrate-binding protein	Unknown	-2.67
A1B042	Pden_0774	50S ribosomal protein L6	Cytoplasmic	-2.68
A1B4S6	Pden_2433	50S ribosomal protein L28	Cytoplasmic	-2.70
A1B877	Pden_3654	60 kDa chaperonin (GroEL protein) (Protein Cpn60)	Cytoplasmic	-2.71
A1B9V2	Pden_4232	Peptidylprolyl isomerase (EC 5.2.1.8)	Cytoplasmic	-2.71
A1BAA7	Pden_4387	Carbohydrate ABC transporter substrate-binding protein, CUT1 family	Periplasmic	-2.72
A1BAD6	Pden_4416	Succinate semialdehyde dehydrogenase (EC 1.2.1.16)	Cytoplasmic	-2.73
A1B1V8	Pden_1401	DNA-directed RNA polymerase subunit omega (RNAP omega subunit) (EC 2.7.7.6) (RNA polymerase omega subunit) (Transcriptase subunit omega)	Cytoplasmic	-2.78
A1B013	Pden_0745	50S ribosomal protein L10	Cytoplasmic	-2.79
A1B0F6	Pden_0889	50S ribosomal protein L9	Cytoplasmic	-2.84
A1B045	Pden_0777	50S ribosomal protein L30	Unknown	-2.86
A1B876	Pden_3653	Cold-shock DNA-binding protein family	Cytoplasmic	-2.90
A1B5M8	Pden_2738	Chromosome partition protein Smc	Cytoplasmic	-2.90
A1BAE1	Pden_4421	Phosphonate metabolism protein PhnM	Cytoplasmic	-2.90
A1B372	Pden_1869	Sporulation domain protein	Unknown	-2.91
A1B011	Pden_0743	50S ribosomal protein L11	Cytoplasmic	-2.92
A1B1U1	Pden_1384	Ribonucleoside-diphosphate reductase class II (EC 1.17.4.-)	Cytoplasmic	-2.94
A1B082	Pden_0814	D-3-phosphoglycerate dehydrogenase (EC 1.1.1.95)	Cytoplasmic	-2.96
A1B034	Pden_0766	50S ribosomal protein L16	Cytoplasmic	-2.97
A1BAK7	Pden_4487	Cell division protein FtsZ	Cytoplasmic	-3.01
A1B2X1	Pden_1768	Acyl carrier protein (ACP)	Cytoplasmic	-3.03
A1B044	Pden_0776	30S ribosomal protein S5	Cytoplasmic	-3.07
A1AZJ3	Pden_0575	Aspartyl/glutamyl-tRNA(Asn/Gln) amidotransferase subunit C (Asp/Glu-ADT subunit C) (EC 6.3.5.-)	Cytoplasmic	-3.09
A1B2U6	Pden_1743	DEAD/DEAH box helicase domain protein	Cytoplasmic	-3.12
A1B1T3	Pden_1376	Lipoyl synthase (EC 2.8.1.8) (Lip-syn) (LS) (Lipoate synthase) (Lipoic acid synthase) (Sulfur insertion protein LipA)	Cytoplasmic	-3.15
A1BC33	Pden_5017	Uncharacterized protein	Unknown	-3.16
A1B037	Pden_0769	50S ribosomal protein L14	Cytoplasmic	-3.18
A1B050	Pden_0782	30S ribosomal protein S13	Cytoplasmic	-3.21
A1B4Q7	Pden_2413	ABC transporter related protein	Cytoplasmic/Membrane	-3.21
A1BCD5	Pden_5119	NADPH-dependent FMN reductase	Unknown	-3.22
A1B5W0	Pden_2820	RNA polymerase sigma factor	Cytoplasmic	-3.22
Q51664	Pden_2482	Protein NorQ	Cytoplasmic	-3.26

A1AZS5	Pden_0657	Uncharacterized protein	Unknown	-3.44
A1B053	Pden_0785	50S ribosomal protein L17	Cytoplasmic	-3.50
A1B9I1	Pden_4109	Putative sulfonate/nitrate transport system substrate-binding protein	Cytoplasmic	-3.51
A1B8E8	Pden_3725	Elongation factor Ts (EF-Ts)	Cytoplasmic	-3.52
A1BA97	Pden_4377	Ribonucleoside-diphosphate reductase (EC 1.17.4.1)	Cytoplasmic	-3.53
A1B4B5	Pden_2267	30S ribosomal protein S21	Unknown	-3.57
A1BAN4	Pden_4514	30S ribosomal protein S20	Cytoplasmic	-3.59
A1B4Q5	Pden_2411	Outer membrane efflux protein	Cytoplasmic	-3.65
A1B5N2	Pden_2742	Uncharacterized protein	Unknown	-3.72
A1AZS3	Pden_0655	Nitrite/sulfite reductase, hemoprotein beta-component, ferrodoxin domain protein	Cytoplasmic	-3.74
A1B6B0	Pden_2970	30S ribosomal protein S9	Cytoplasmic	-3.87
A1BCE1	Pden_5125	Putative monooxygenase protein	Cytoplasmic	-3.88
A1B6C8	Pden_2989	Anaerobic ribonucleoside-triphosphate reductase	Cytoplasmic	-4.39
A1B4Q1	Pden_2407	Mur ligase, middle domain protein	Cytoplasmic/Membrane	-4.39
A1BB78	Pden_4711	Cell division topological specificity factor	Cytoplasmic	-4.56
A1B038	Pden_0770	50S ribosomal protein L24	Cytoplasmic	-4.68
A1B014	Pden_0746	50S ribosomal protein L7/L12	Unknown	-4.87
A1B040	Pden_0772	30S ribosomal protein S14	Cytoplasmic	-5.18
A1BA98	Pden_4378	Ribonucleoside-diphosphate reductase subunit beta (EC 1.17.4.1)	Cytoplasmic	-5.24
Q51703	Pden_2491	Protein NirD	Cytoplasmic	-5.74
Q51701	Pden_2488	Uroporphyrinogen-III C-methyltransferase (Urogen III methylase) (EC 2.1.1.107) (SUMT) (Uroporphyrinogen III methylase) (UROM)	Cytoplasmic	-6.02
A1AZG3	Pden_0545	Phosphoglucosamine mutase (EC 5.4.2.10)	Cytoplasmic	-6.03
A1B0F7	Pden_0890	30S ribosomal protein S18	Cytoplasmic	-6.88
A1B5Q0	Pden_2760	30S ribosomal protein S15	Cytoplasmic	-7.07
A1B656	Pden_2916	Biotin synthase 2 (EC 2.8.1.6)	Cytoplasmic	-7.22
A1B4Q4	Pden_2410	Uncharacterized protein	Cytoplasmic	-8.86
A1BC23	Pden_5007	Monooxygenase, NtaA/SnaA/SoxA family	Cytoplasmic	-20.14
A1BBD2	Pden_4765	C4-dicarboxylate transport protein	Cytoplasmic/Membrane	-21.26
A1AXZ7	Pden_0024	Uncharacterized protein	Unknown	Exclusive pH 7.2
A1AXZ9	Pden_0026	Amino acid/amide ABC transporter substrate-binding protein, HAAT family	Unknown	Exclusive pH 7.2
A1AY00	Pden_0027	40-residue YVTN family beta-propeller repeat protein	Unknown	Exclusive pH 7.2
A1AY21	Pden_0048	AMP-dependent synthetase and ligase	Cytoplasmic	Exclusive pH 7.2
A1AY22	Pden_0049	Amino acid/amide ABC transporter ATP-binding protein 1, HAAT family	Cytoplasmic/Membrane	Exclusive pH 7.2

A1AY26	Pden_0053	Amino acid/amide ABC transporter ATP-binding protein 2, HAAT family	Cytoplasmic/Membrane	Exclusive pH 7.2
A1AY27	Pden_0054	Phenylacetate-CoA ligase, putative	Cytoplasmic	Exclusive pH 7.2
A1AY32	Pden_0059	Glutathione S-transferase, N-terminal domain	Cytoplasmic/Membrane	Exclusive pH 7.2
A1AY34	Pden_0061	Uncharacterized protein	Unknown	Exclusive pH 7.2
A1AY52	Pden_0079	Two component transcriptional regulator, winged helix family	Unknown	Exclusive pH 7.2
A1AYG5	Pden_0193	TRAP dicarboxylate transporter-DctP subunit	Unknown	Exclusive pH 7.2
A1AYS6	Pden_0306	Uncharacterized protein	Cytoplasmic/Membrane	Exclusive pH 7.2
A1AYS9	Pden_0309	Periplasmic iron-binding protein	Unknown	Exclusive pH 7.2
A1AZ67	Pden_0447	Uncharacterized protein	Cytoplasmic	Exclusive pH 7.2
A1AZE2	Pden_0524	Purine nucleoside phosphorylase (EC 2.4.2.1) (Inosine-guanosine phosphorylase)	Cytoplasmic	Exclusive pH 7.2
A1AZI3	Pden_0565	Uncharacterized protein	Unknown	Exclusive pH 7.2
A1AZQ0	Pden_0632	SsrA-binding protein (Small protein B)	Cytoplasmic	Exclusive pH 7.2
A1AZQ6	Pden_0638	Uncharacterized protein	Cytoplasmic/Membrane	Exclusive pH 7.2
A1AZT7	Pden_0669	3-oxoacyl-[acyl-carrier-protein] synthase III (EC 2.3.1.41)	Cytoplasmic	Exclusive pH 7.2
A1B096	Pden_0828	Hydratase/decarboxylase	Cytoplasmic/Membrane	Exclusive pH 7.2
A1B0B5	Pden_0847	Polysaccharide biosynthesis protein	Cytoplasmic/Membrane	Exclusive pH 7.2
A1B0W3	Pden_1046	Transcriptional regulator, LacI family	Cytoplasmic	Exclusive pH 7.2
A1B0Y2	Pden_1067	OsmC family protein	Cytoplasmic	Exclusive pH 7.2
A1B1F4	Pden_1243	3-oxoacyl-[acyl-carrier-protein] synthase II (EC 2.3.1.41)	Cytoplasmic/Membrane	Exclusive pH 7.2
A1B1G1	Pden_1250	Binding-protein-dependent transport systems inner membrane component	Cytoplasmic/Membrane	Exclusive pH 7.2
A1B1G2	Pden_1251	ABC transporter related protein	Cytoplasmic/Membrane	Exclusive pH 7.2
A1B1I8	Pden_1277	Uracil-xanthine permease	Cytoplasmic/Membrane	Exclusive pH 7.2
A1B1I9	Pden_1278	Uncharacterized protein	Cytoplasmic/Membrane	Exclusive pH 7.2
A1B1S0	Pden_1363	Isocitrate lyase (EC 4.1.3.1)	Cytoplasmic	Exclusive pH 7.2
A1B1S1	Pden_1364	Malate synthase (EC 2.3.3.9)	Cytoplasmic	Exclusive pH 7.2
A1B1Z5	Pden_1438	Pathogenesis-related protein	Cytoplasmic	Exclusive pH 7.2
A1B1Z6	Pden_1439	Uncharacterized protein	Cytoplasmic	Exclusive pH 7.2
A1B2C0	Pden_1564	TRAP dicarboxylate transporter, DctM subunit	Cytoplasmic/Membrane	Exclusive pH 7.2

A1B2C1	Pden_1565	Tripartite ATP-independent periplasmic transporter, DctQ component	Cytoplasmic/Membrane	Exclusive pH 7.2
A1B2M3	Pden_1670	Binding-protein-dependent transport systems inner membrane component	Cytoplasmic/Membrane	Exclusive pH 7.2
A1B2S6	Pden_1723	Uncharacterized protein	Unknown	Exclusive pH 7.2
A1B2T6	Pden_1733	Iron permease FTR1	Cytoplasmic/Membrane	Exclusive pH 7.2
A1B336	Pden_1833	Peptidase M19, renal dipeptidase	Unknown	Exclusive pH 7.2
A1B3M1	Pden_2023	Redoxin domain protein	Cytoplasmic/Membrane	Exclusive pH 7.2
A1B3U6	Pden_2098	Alkane 1-monooxygenase (EC 1.14.15.3)	Cytoplasmic/Membrane	Exclusive pH 7.2
A1B3Y2	Pden_2134	Aminotransferase (EC 2.6.1.-)	Cytoplasmic	Exclusive pH 7.2
A1B445	Pden_2197	Export-related chaperone CsaA	Cytoplasmic	Exclusive pH 7.2
A1B482	Pden_2234	NADH-quinone oxidoreductase subunit K (EC 1.6.5.11) (NADH dehydrogenase I subunit K) (NADH dehydrogenase I, subunit 11) (NADH-quinone oxidoreductase subunit 11) (NQO11) (NDH-1 subunit K) (NDH-1, subunit 11)	Cytoplasmic/Membrane	Exclusive pH 7.2
A1B4A1	Pden_2253	Glutathione-dependent formaldehyde-activating, GFA	Unknown	Exclusive pH 7.2
A1B4G9	Pden_2322	Transcriptional regulator, AsnC family	Cytoplasmic	Exclusive pH 7.2
A1B4J9	Pden_2353	Uncharacterized protein	Cytoplasmic	Exclusive pH 7.2
A1B4K0	Pden_2354	Two component transcriptional regulator, LuxR family	Cytoplasmic	Exclusive pH 7.2
A1B4T4	Pden_2441	Serine/threonine protein kinase	Cytoplasmic	Exclusive pH 7.2
A1B4T7	Pden_2444	Uncharacterized protein	Cytoplasmic	Exclusive pH 7.2
A1B522	Pden_2529	Cob(I)yrinic acid a,c-diamide adenosyltransferase (EC 2.5.1.17)	Cytoplasmic	Exclusive pH 7.2
A1B530	Pden_2537	Precorrin-6A reductase (EC 1.3.1.54)	Cytoplasmic	Exclusive pH 7.2
A1B5B5	Pden_2622	Uncharacterized protein	Unknown	Exclusive pH 7.2
A1B5E7	Pden_2654	Efflux transporter, RND family, MFP subunit	Unknown	Exclusive pH 7.2
A1B5F0	Pden_2657	Uncharacterized protein	Cytoplasmic	Exclusive pH 7.2
A1B5F7	Pden_2664	Putative transcriptional regulator, ModE family	Unknown	Exclusive pH 7.2
A1B5I2	Pden_2689	Flavin reductase domain protein, FMN-binding protein	Unknown	Exclusive pH 7.2
A1B5N5	Pden_2745	Multiple monosaccharide-binding protein	Periplasmic	Exclusive pH 7.2
A1B5Q9	Pden_2769	Glutathione S-transferase, N-terminal domain	Cytoplasmic	Exclusive pH 7.2
A1B5U2	Pden_2802	Uncharacterized protein	Unknown	Exclusive pH 7.2

A1B5W6	Pden_2826	Protein FdhE homolog	Cytoplasmic	Exclusive pH 7.2
A1B645	Pden_2905	Short chain enoyl-CoA hydratase (EC 4.2.1.17)	Cytoplasmic	Exclusive pH 7.2
A1B649	Pden_2909	AMP-dependent synthetase and ligase	Cytoplasmic	Exclusive pH 7.2
A1B690	Pden_2950	Uncharacterized protein	Unknown	Exclusive pH 7.2
A1B693	Pden_2953	Transcriptional regulator, AraC family with amidase-like domain	Cytoplasmic/Membrane	Exclusive pH 7.2
A1B6A7	Pden_2967	Enoyl-CoA hydratase/isomerase	Cytoplasmic	Exclusive pH 7.2
A1B750	Pden_3266	Uncharacterized protein UPF0065	Unknown	Exclusive pH 7.2
A1B751	Pden_3267	Amidohydrolase 2	Cytoplasmic	Exclusive pH 7.2
A1B7R6	Pden_3490	4-hydroxybenzoate 3-monooxygenase (EC 1.14.13.2)	Cytoplasmic	Exclusive pH 7.2
A1B7S3	Pden_3497	TRAP dicarboxylate transporter-DctP subunit	Periplasmic	Exclusive pH 7.2
A1B7V3	Pden_3527	TonB-dependent receptor	Outer Membrane	Exclusive pH 7.2
A1B7V6	Pden_3530	Siderophore-interacting protein	Cytoplasmic	Exclusive pH 7.2
A1B7V7	Pden_3531	Periplasmic binding protein	Unknown	Exclusive pH 7.2
A1B7Z3	Pden_3569	Formamidase (EC 3.5.1.49)	Cytoplasmic	Exclusive pH 7.2
A1B822	Pden_3599	Uncharacterized protein	Unknown	Exclusive pH 7.2
A1B857	Pden_3634	Uncharacterized protein	Unknown	Exclusive pH 7.2
A1B861	Pden_3638	Appr-1-p processing domain protein	Cytoplasmic	Exclusive pH 7.2
A1B864	Pden_3641	Hydroxymethylglutaryl-CoA lyase (EC 4.1.3.4)	Cytoplasmic	Exclusive pH 7.2
A1B865	Pden_3642	Methylglutaconyl-CoA hydratase (EC 4.2.1.18)	Cytoplasmic	Exclusive pH 7.2
A1B879	Pden_3656	GumN family protein	Unknown	Exclusive pH 7.2
A1B884	Pden_3661	MaoC domain protein dehydratase	Cytoplasmic	Exclusive pH 7.2
A1B8C2	Pden_3699	Heme A synthase (HAS) (EC 1.3.-.-) (Cytochrome aa3-controlling protein) (ctaA)	Cytoplasmic/Membrane	Exclusive pH 7.2
A1B8D1	Pden_3708	Uncharacterized protein	Unknown	Exclusive pH 7.2
A1B8G6	Pden_3743	Uncharacterized protein	Cytoplasmic	Exclusive pH 7.2
A1B8L9	Pden_3797	Transcriptional regulator, RpiR family	Cytoplasmic	Exclusive pH 7.2
A1B8P6	Pden_3824	CoA-binding domain protein	Cytoplasmic	Exclusive pH 7.2
A1B8S2	Pden_3850	Uncharacterized protein	Unknown	Exclusive pH 7.2
A1B949	Pden_3977	Cytochrome c-type biogenesis protein CcmF	Cytoplasmic/Membrane	Exclusive pH 7.2
A1B9E5	Pden_4073	DNA primase (EC 2.7.7.-)	Cytoplasmic	Exclusive pH 7.2
A1B9H6	Pden_4104	L-carnitine dehydratase/bile acid-inducible protein F	Cytoplasmic	Exclusive pH 7.2

A1B9I2	Pden_4110	HpcH/HpaI aldolase	Cytoplasmic	Exclusive pH 7.2
A1B9I3	Pden_4111	Uncharacterized protein	Cytoplasmic	Exclusive pH 7.2
A1B9I4	Pden_4112	L-carnitine dehydratase/bile acid-inducible protein F	Cytoplasmic	Exclusive pH 7.2
A1B9I6	Pden_4116	Uncharacterized protein	Cytoplasmic/Membrane	Exclusive pH 7.2
A1B9I7	Pden_4117	Pimeloyl-CoA biosynthesis protein BioC	Cytoplasmic	Exclusive pH 7.2
A1B9I8	Pden_4118	Transcriptional regulator, LysR family	Cytoplasmic	Exclusive pH 7.2
A1B9I9	Pden_4119	Fumarate hydratase class I (EC 4.2.1.2)	Cytoplasmic	Exclusive pH 7.2
A1B9J0	Pden_4120	TRAP dicarboxylate transporter, DctM subunit	Cytoplasmic/Membrane	Exclusive pH 7.2
A1B9J1	Pden_4121	Tripartite ATP-independent periplasmic transporter, DctQ component	Cytoplasmic/Membrane	Exclusive pH 7.2
A1B9S3	Pden_4203	Periplasmic binding protein	Unknown	Exclusive pH 7.2
A1B9S8	Pden_4208	Acetate kinase (EC 2.7.2.1) (Acetokinase)	Cytoplasmic	Exclusive pH 7.2
A1BAB8	Pden_4398	FAD linked oxidase domain protein	Cytoplasmic	Exclusive pH 7.2
A1BAC2	Pden_4402	Uncharacterized protein	Cytoplasmic/Membrane	Exclusive pH 7.2
A1BAI6	Pden_4466	Antifreeze protein, type I	Unknown	Exclusive pH 7.2
A1BAK1	Pden_4481	Enoyl-CoA hydratase/isomerase	Cytoplasmic	Exclusive pH 7.2
A1BAN5	Pden_4515	3-demethylubiquinone-9 3-methyltransferase	Unknown	Exclusive pH 7.2
A1BAN9	Pden_4519	Activator of Hsp90 ATPase 1 family protein	Cytoplasmic	Exclusive pH 7.2
A1BAR8	Pden_4549	Iron-containing alcohol dehydrogenase	Cytoplasmic	Exclusive pH 7.2
A1BAS1	Pden_4552	Na+/solute symporter	Cytoplasmic/Membrane	Exclusive pH 7.2
A1BAX2	Pden_4603	Putative ABC transporter binding protein component	Periplasmic	Exclusive pH 7.2
A1BAY5	Pden_4616	Dihydropyrimidinase (EC 3.5.2.2)	Cytoplasmic	Exclusive pH 7.2
A1BB09	Pden_4642	Uncharacterized protein	Unknown	Exclusive pH 7.2
A1BB12	Pden_4645	Transcriptional regulator, TetR family	Cytoplasmic	Exclusive pH 7.2
A1BBC5	Pden_4758	Branched-chain alpha-keto acid dehydrogenase E1 component (EC 1.2.4.4)	Cytoplasmic	Exclusive pH 7.2
A1BBF9	Pden_4792	3-hydroxyacyl-CoA dehydrogenase (EC 1.1.1.35)	Cytoplasmic	Exclusive pH 7.2
A1BBG4	Pden_4797	Amino acid/amide ABC transporter substrate-binding protein, HAAT family	Periplasmic	Exclusive pH 7.2
A1BBG8	Pden_4801	Phenylacetate-coenzyme A ligase (EC 6.2.1.30) (Phenylacetyl-CoA ligase)	Cytoplasmic	Exclusive pH 7.2

A1BBN9	Pden_4873	Putative spermidine/putrescine transport system substrate-binding protein	Periplasmic	Exclusive pH 7.2
A1BBQ4	Pden_4888	Substrate-binding region of ABC-type glycine betaine transport system	Cytoplasmic/Membrane	Exclusive pH 7.2
A1BBZ8	Pden_4982	Short-chain dehydrogenase/reductase SDR	Unknown	Exclusive pH 7.2
A1BBZ9	Pden_4983	Alpha/beta hydrolase fold protein	Cytoplasmic	Exclusive pH 7.2
A1BCC8	Pden_5112	Integral membrane sensor signal transduction histidine kinase	Cytoplasmic/Membrane	Exclusive pH 7.2
A1BCC9	Pden_5113	Response regulator receiver protein	Cytoplasmic	Exclusive pH 7.2
A1BCD0	Pden_5114	DNA polymerase III, epsilon subunit (EC 2.7.7.7)	Unknown	Exclusive pH 7.2
A1AY04	Pden_0031	Uncharacterized protein	Unknown	Exclusive pH 7.2
A1AY05	Pden_0032	Molybdenum cofactor guanylyltransferase (MoCo guanylyltransferase) (EC 2.7.7.77) (GTP:molybdopterin guanylyltransferase) (Mo-MPT guanylyltransferase) (Molybdopterin guanylyltransferase) (Molybdopterin-guanine dinucleotide synthase) (MGD synthase)	Cytoplasmic	Exclusive pH 7.2
A1AY84	Pden_0111	Uncharacterized protein	Cytoplasmic/Membrane	Exclusive pH 7.2
A1AYI0	Pden_0210	Uncharacterized protein	Unknown	Exclusive pH 7.2
A1AYS1	Pden_0301	L-seryl-tRNA(Sec) selenium transferase (EC 2.9.1.1) (Selenocysteine synthase) (Sec synthase) (Selenocysteinyl-tRNA(Sec) synthase)	Cytoplasmic	Exclusive pH 7.2
A1AYT3	Pden_0313	Uncharacterized protein	Unknown	Exclusive pH 7.2
A1AZC1	Pden_0503	Ribose ABC transporter ATP-binding protein / fructose ABC transporter ATP-binding protein / mannose ABC transporter ATP-binding protein	Cytoplasmic/Membrane	Exclusive pH 7.2
A1AZE3	Pden_0525	Nucleoside ABC transporter membrane protein	Cytoplasmic/Membrane	Exclusive pH 7.2
A1AZJ7	Pden_0579	Ribosomal RNA large subunit methyltransferase J (EC 2.1.1.266) (23S rRNA (adenine(2030)-N6)-methyltransferase) (23S rRNA m6A2030 methyltransferase)	Cytoplasmic	Exclusive pH 7.2
A1AZQ7	Pden_0639	Uncharacterized protein	Cytoplasmic	Exclusive pH 7.2
A1AZR2	Pden_0644	Uncharacterized protein	Cytoplasmic/Membrane	Exclusive pH 7.2
A1AZV7	Pden_0689	tRNA(Ile)-lysidine synthase (EC 6.3.4.19) (tRNA(Ile)-2-lysyl-cytidine synthase) (tRNA(Ile)-lysidine synthetase)	Cytoplasmic	Exclusive pH 7.2

A1AZW6	Pden_0698	Multisubunit potassium/proton antiporter, PhaC subunit	Cytoplasmic/Membrane	Exclusive pH 7.2
A1B056	Pden_0788	Recombination protein MgsA	Cytoplasmic	Exclusive pH 7.2
A1B0B9	Pden_0851	Nicotinamidase (EC 3.5.1.19)	Cytoplasmic	Exclusive pH 7.2
A1B0C6	Pden_0858	Pyridoxine/pyridoxamine 5'-phosphate oxidase (EC 1.4.3.5) (PNP/PMP oxidase) (PNPOx) (Pyridoxal 5'-phosphate synthase)	Cytoplasmic	Exclusive pH 7.2
A1B0L4	Pden_0947	Glycine betaine/L-proline ABC transporter, ATPase subunit	Cytoplasmic/Membrane	Exclusive pH 7.2
A1B1A4	Pden_1193	Glycine betaine/L-proline ABC transporter, ATPase subunit	Cytoplasmic/Membrane	Exclusive pH 7.2
A1B1C6	Pden_1215	Activator of Hsp90 ATPase 1 family protein	Unknown	Exclusive pH 7.2
A1B1G0	Pden_1249	Binding-protein-dependent transport systems inner membrane component	Cytoplasmic/Membrane	Exclusive pH 7.2
A1B1L1	Pden_1300	Uncharacterized protein	Cytoplasmic/Membrane	Exclusive pH 7.2
A1B1N8	Pden_1327	Binding-protein-dependent transport systems inner membrane component	Cytoplasmic/Membrane	Exclusive pH 7.2
A1B1N9	Pden_1328	Extracellular solute-binding protein, family 5	Periplasmic	Exclusive pH 7.2
A1B2A5	Pden_1549	Uncharacterized protein	Unknown	Exclusive pH 7.2
A1B2K7	Pden_1654	Lysine--tRNA ligase (EC 6.1.1.6)	Cytoplasmic	Exclusive pH 7.2
A1B2R8	Pden_1715	Amino acid/amide ABC transporter ATP-binding protein 2, HAAT family	Cytoplasmic	Exclusive pH 7.2
A1B2X4	Pden_1771	Integral membrane sensor signal transduction histidine kinase	Cytoplasmic/Membrane	Exclusive pH 7.2
A1B2Y2	Pden_1779	Uncharacterized conserved protein UCP032025	Unknown	Exclusive pH 7.2
A1B2Y9	Pden_1786	Response regulator receiver protein	Cytoplasmic	Exclusive pH 7.2
A1B349	Pden_1846	Cbb3-type cytochrome oxidase component	Unknown	Exclusive pH 7.2
A1B3I3	Pden_1985	Putative pterin-4-alpha-carbinolamine dehydratase (PHS) (EC 4.2.1.96) (4-alpha-hydroxy-tetrahydropterin dehydratase) (Pterin carbinolamine dehydratase) (PCD)	Cytoplasmic	Exclusive pH 7.2
A1B3N0	Pden_2032	Ammonium transporter	Cytoplasmic/Membrane	Exclusive pH 7.2
A1B3T5	Pden_2087	Uncharacterized protein	Cytoplasmic	Exclusive pH 7.2
A1B3X5	Pden_2127	ABC transporter related protein	Cytoplasmic	Exclusive pH 7.2
A1B461	Pden_2213	Prolipoprotein diacylglyceryl transferase (EC 2.4.99.-)	Cytoplasmic/Membrane	Exclusive pH 7.2
A1B4B4	Pden_2266	Transcriptional regulator, AsnC family	Cytoplasmic	Exclusive pH 7.2
A1B4I7	Pden_2340	Molybdopterin synthase subunit MoaE	Cytoplasmic	Exclusive pH 7.2

A1B4J8	Pden_2352	Histidine kinase	Cytoplasmic/Membrane	Exclusive pH 7.2
A1B4M4	Pden_2378	Putative RNA methylase	Cytoplasmic	Exclusive pH 7.2
A1B4T6	Pden_2443	Uncharacterized conserved protein UCP028301	Cytoplasmic	Exclusive pH 7.2
A1B535	Pden_2542	Precorrin-6A synthase (Deacetylating) (EC 2.1.1.152)	Cytoplasmic	Exclusive pH 7.2
A1B577	Pden_2584	SAM-dependent methyltransferase	Cytoplasmic	Exclusive pH 7.2
A1B5B0	Pden_2617	Iron-containing alcohol dehydrogenase	Cytoplasmic	Exclusive pH 7.2
A1B5G1	Pden_2668	Uncharacterized protein	Unknown	Exclusive pH 7.2
A1B5P8	Pden_2758	Uncharacterized protein	Cytoplasmic	Exclusive pH 7.2
A1B5Q8	Pden_2768	HI0933 family protein	Cytoplasmic	Exclusive pH 7.2
A1B5Z7	Pden_2857	Formate dehydrogenase beta subunit (EC 1.2.1.2)	Cytoplasmic	Exclusive pH 7.2
A1B659	Pden_2919	Adenosylmethionine-8-amino-7-oxononanoate aminotransferase apoenzyme (EC 2.6.1.62)	Unknown	Exclusive pH 7.2
A1B671	Pden_2931	Gluconate transporter	Cytoplasmic/Membrane	Exclusive pH 7.2
A1B680	Pden_2940	Transcriptional regulator, TetR family	Cytoplasmic	Exclusive pH 7.2
A1B6A8	Pden_2968	Uncharacterized protein	Cytoplasmic	Exclusive pH 7.2
A1B6D2	Pden_2993	Pyrrolo-quinoline quinone	Periplasmic	Exclusive pH 7.2
A1B6G3	Pden_3024	Transcriptional regulator, BadM/Rrf2 family	Cytoplasmic	Exclusive pH 7.2
A1B735	Pden_3248	Regulatory protein, IclR	Unknown	Exclusive pH 7.2
A1B7G6	Pden_3387	TRAP dicarboxylate transporter, DctP subunit	Periplasmic	Exclusive pH 7.2
A1B7P5	Pden_3468	Putative transcriptional regulator, Crp/Fnr family	Cytoplasmic	Exclusive pH 7.2
A1B7Q6	Pden_3480	TRAP dicarboxylate transporter, DctP subunit	Periplasmic	Exclusive pH 7.2
A1B7Y0	Pden_3554	Uncharacterized protein	Cytoplasmic	Exclusive pH 7.2
A1B8A2	Pden_3679	Uncharacterized protein	Cytoplasmic	Exclusive pH 7.2
A1B8G8	Pden_3745	Uncharacterized protein	Unknown	Exclusive pH 7.2
A1B8W1	Pden_3889	Acriflavin resistance protein	Cytoplasmic/Membrane	Exclusive pH 7.2
A1B8Y6	Pden_3914	Uncharacterized protein	Cytoplasmic/Membrane	Exclusive pH 7.2
A1B8Z1	Pden_3919	D-3-hydroxyaspartate aldolase (EC 4.1.3.-)	Cytoplasmic	Exclusive pH 7.2
A1B8Z2	Pden_3920	Pyridoxal-5'-phosphate-dependent enzyme, beta subunit	Cytoplasmic	Exclusive pH 7.2
A1B905	Pden_3933	Transcriptional regulator, AraC family with amidase-like domain	Cytoplasmic	Exclusive pH 7.2
A1B998	Pden_4026	Uncharacterized protein	Cytoplasmic	Exclusive pH 7.2

A1B9I5	Pden_4115	Glyoxalase/bleomycin resistance protein/dioxygenase	Cytoplasmic	Exclusive pH 7.2
A1B9X7	Pden_4257	Molybdopterin dehydrogenase, FAD-binding protein	Cytoplasmic	Exclusive pH 7.2
A1B9Y0	Pden_4260	Guanine deaminase (EC 3.5.4.3)	Cytoplasmic	Exclusive pH 7.2
A1B9Y5	Pden_4265	Transcriptional regulator, GntR family	Cytoplasmic	Exclusive pH 7.2
A1B9Z2	Pden_4272	Amino acid/amide ABC transporter substrate-binding protein, HAAT family	Unknown	Exclusive pH 7.2
A1BAB9	Pden_4399	FAD linked oxidase domain protein	Cytoplasmic	Exclusive pH 7.2
A1BAD5	Pden_4415	Transcriptional regulator, XRE family	Cytoplasmic	Exclusive pH 7.2
A1BAF4	Pden_4434	Putative aminotransferase, class IV	Unknown	Exclusive pH 7.2
A1BAS2	Pden_4553	Cyclic nucleotide-binding protein	Cytoplasmic	Exclusive pH 7.2
A1BAW3	Pden_4594	Thiamine pyrophosphate enzyme domain protein TPP-binding protein	Cytoplasmic	Exclusive pH 7.2
A1BB76	Pden_4709	Uncharacterized protein	Unknown	Exclusive pH 7.2
A1BBC4	Pden_4757	Branched-chain alpha-keto acid dehydrogenase E1 component (EC 1.2.4.4)	Cytoplasmic	Exclusive pH 7.2
A1BBH8	Pden_4811	Beta-ketoadipyl CoA thiolase (EC 2.3.1.9)	Cytoplasmic	Exclusive pH 7.2
A1BBT1	Pden_4915	Transcriptional regulator, DeoR family	Cytoplasmic	Exclusive pH 7.2
A1BBW5	Pden_4949	Uncharacterized protein	Cytoplasmic	Exclusive pH 7.2
A1BC01	Pden_4985	Pyruvate dehydrogenase (Acetyl-transferring) (EC 1.2.4.1)	Cytoplasmic	Exclusive pH 7.2
A1BC12	Pden_4996	CoA-binding domain protein	Cytoplasmic	Exclusive pH 7.2
A1BC53	Pden_5037	ABC transporter related protein	Cytoplasmic	Exclusive pH 7.2
A1BC82	Pden_5066	TRAP dicarboxylate transporter, DctP subunit	Periplasmic	Exclusive pH 7.2
P52220	Pden_1411	Heme exporter protein C (Cytochrome c-type biogenesis protein CcmC)	Cytoplasmic/Membrane	Exclusive pH 7.2
A1AXX8	Pden_0005	Ribosomal RNA small subunit methyltransferase G (EC 2.1.1.170) (16S rRNA 7-methylguanosine methyltransferase) (16S rRNA m7G methyltransferase)	Cytoplasmic	Exclusive pH 7.5
A1AY77	Pden_0104	Uncharacterized protein	Unknown	Exclusive pH 7.5
A1AY88	Pden_0115	YecA family protein	Unknown	Exclusive pH 7.5
A1AYK1	Pden_0231	Transcriptional regulator, LysR family	Cytoplasmic	Exclusive pH 7.5
A1AYL3	Pden_0243	Succinate semialdehyde dehydrogenase (EC 1.2.1.16)	Cytoplasmic	Exclusive pH 7.5
A1AZ22	Pden_0402	NAD-dependent epimerase/dehydratase	Unknown	Exclusive pH 7.5
A1AZ62	Pden_0442	Uncharacterized protein	Unknown	Exclusive pH 7.5

A1AZC6	Pden_0508	Uncharacterized protein	Cytoplasmic	Exclusive pH 7.5
A1AZG7	Pden_0549	GCN5-related N-acetyltransferase	Cytoplasmic	Exclusive pH 7.5
A1AZL2	Pden_0594	Fmu (Sun) domain protein	Cytoplasmic	Exclusive pH 7.5
A1AZM3	Pden_0605	Thiamine diphosphokinase	Cytoplasmic/Membrane	Exclusive pH 7.5
A1AZW4	Pden_0696	Crossover junction endodeoxyribonuclease RuvC (EC 3.1.22.4) (Holliday junction nuclease RuvC) (Holliday junction resolvase RuvC)	Cytoplasmic	Exclusive pH 7.5
A1B083	Pden_0815	Metallophosphoesterase	Cytoplasmic	Exclusive pH 7.5
A1B086	Pden_0818	Hemimethylated DNA binding protein	Unknown	Exclusive pH 7.5
A1B1A3	Pden_1192	Pyrroline-5-carboxylate reductase (P5C reductase) (P5CR) (EC 1.5.1.2) (PCA reductase)	Cytoplasmic	Exclusive pH 7.5
A1B1B7	Pden_1206	Urease accessory protein UreF	Unknown	Exclusive pH 7.5
A1B1C3	Pden_1212	Urease accessory protein UreD	Cytoplasmic	Exclusive pH 7.5
A1B1R2	Pden_1354	Uncharacterized protein	Cytoplasmic	Exclusive pH 7.5
A1B1V5	Pden_1398	Gamma-glutamylputrescine oxidase	Cytoplasmic	Exclusive pH 7.5
A1B1W4	Pden_1407	Uncharacterized protein	Cytoplasmic/Membrane	Exclusive pH 7.5
A1B1X1	Pden_1414	Uncharacterized protein	Cytoplasmic/Membrane	Exclusive pH 7.5
A1B2F4	Pden_1598	Uncharacterized protein	Unknown	Exclusive pH 7.5
A1B2T5	Pden_1732	Putative glutathione S-transferase	Cytoplasmic	Exclusive pH 7.5
A1B3F2	Pden_1954	SAM-dependent methyltransferase (EC 2.1.1.-)	Cytoplasmic	Exclusive pH 7.5
A1B3G7	Pden_1969	Uncharacterized protein	Cytoplasmic	Exclusive pH 7.5
A1B469	Pden_2221	Phosphopentomutase (EC 5.4.2.7) (Phosphodeoxyribomutase)	Cytoplasmic	Exclusive pH 7.5
A1B4M8	Pden_2382	Isochorismate synthase	Cytoplasmic	Exclusive pH 7.5
A1B4Q3	Pden_2409	Uncharacterized protein	Unknown	Exclusive pH 7.5
A1B521	Pden_2528	Uncharacterized protein	Unknown	Exclusive pH 7.5
A1B563	Pden_2570	Uncharacterized protein	Cytoplasmic	Exclusive pH 7.5
A1B569	Pden_2576	ABC transporter related protein	Cytoplasmic/Membrane	Exclusive pH 7.5
A1B5A4	Pden_2611	Transcriptional regulator, XRE family	Unknown	Exclusive pH 7.5
A1B5F5	Pden_2662	Conserved hypothetical integral membrane protein	Cytoplasmic/Membrane	Exclusive pH 7.5
A1B5H8	Pden_2685	Short chain enoyl-CoA hydratase / Enoyl-CoA hydratase (EC 4.2.1.17)	Cytoplasmic/Membrane	Exclusive pH 7.5
A1B5P7	Pden_2757	Glycosyl transferase, family 25	Cytoplasmic	Exclusive pH 7.5

A1B5V4	Pden_2814	Dephospho-CoA kinase (EC 2.7.1.24) (Dephosphocoenzyme A kinase)	Cytoplasmic	Exclusive pH 7.5
A1B5Y3	Pden_2843	GCN5-related N-acetyltransferase	Cytoplasmic	Exclusive pH 7.5
A1B5Y8	Pden_2848	Phosphotransferase system, phosphocarrier protein HPr	Cytoplasmic	Exclusive pH 7.5
A1B613	Pden_2873	Endonuclease III (EC 4.2.99.18) (DNA-(apurinic or apyrimidinic site) lyase)	Cytoplasmic	Exclusive pH 7.5
A1B662	Pden_2922	Transcriptional regulator, GntR family	Cytoplasmic	Exclusive pH 7.5
A1B6M0	Pden_3083	Putative outer membrane protein	Outer Membrane	Exclusive pH 7.5
A1B6T5	Pden_3148	Protease Do	Periplasmic	Exclusive pH 7.5
A1B7K4	Pden_3427	TRAP dicarboxylate transporter-DctP subunit	Periplasmic	Exclusive pH 7.5
A1B8B6	Pden_3693	Endoribonuclease YbeY (EC 3.1.-.-)	Cytoplasmic	Exclusive pH 7.5
A1B8C1	Pden_3698	tRNA (cytidine/uridine-2'-O-)-methyltransferase TrmJ (EC 2.1.1.200) (tRNA (cytidine(32)/uridine(32)-2'-O)-methyltransferase) (tRNA Cm32/Um32 methyltransferase)	Cytoplasmic	Exclusive pH 7.5
A1B8R0	Pden_3838	Methyltransferase (EC 2.1.1.-)	Cytoplasmic	Exclusive pH 7.5
A1B8T0	Pden_3858	ABC transporter related protein	Cytoplasmic/Membrane	Exclusive pH 7.5
A1B961	Pden_3989	Ppx/GppA phosphatase	Cytoplasmic	Exclusive pH 7.5
A1B9A3	Pden_4031	Uncharacterized protein	Cytoplasmic	Exclusive pH 7.5
A1B9K8	Pden_4138	Zinc import ATP-binding protein ZnuC (EC 3.6.3.-)	Cytoplasmic/Membrane	Exclusive pH 7.5
A1B9V1	Pden_4231	Uncharacterized protein	Unknown	Exclusive pH 7.5
A1BA45	Pden_4325	Two component transcriptional regulator, winged helix family	Cytoplasmic	Exclusive pH 7.5
A1BAK9	Pden_4489	Cell division protein FtsQ	Cytoplasmic	Exclusive pH 7.5
A1BBA6	Pden_4739	Alkylphosphonate utilization operon protein PhnA	Unknown	Exclusive pH 7.5
A1BBG9	Pden_4802	Transcriptional regulator, TetR family	Cytoplasmic	Exclusive pH 7.5
A1BBI6	Pden_4819	Thiolase	Unknown	Exclusive pH 7.5
A1BBN1	Pden_4865	Aldehyde dehydrogenase	Cytoplasmic	Exclusive pH 7.5
A1BBT9	Pden_4923	Transcriptional regulator, IclR family	Cytoplasmic	Exclusive pH 7.5
A1BC19	Pden_5003	Cupin 2, conserved barrel domain protein	Cytoplasmic	Exclusive pH 7.5
A1BC94	Pden_5078	Transcriptional regulator, LacI family	Cytoplasmic	Exclusive pH 7.5
A1BCD8	Pden_5122	Transcriptional regulator, AsnC family	Cytoplasmic	Exclusive pH 7.5
A1BCE2	Pden_5126	Binding-protein-dependent transport systems inner membrane component	Cytoplasmic/Membrane	Exclusive pH 7.5
P54810	Pden_2026	Acetyl-CoA acetyltransferase (EC 2.3.1.9) (Acetoacetyl-CoA thiolase)	Unknown	Exclusive pH 7.5

A1AXX6	Pden_0003	tRNA modification GTPase MnmE (EC 3.6.-.-)	Cytoplasmic	Exclusive pH 7.5
A1AYR0	Pden_0290	Aminotransferase (EC 2.6.1.-)	Cytoplasmic	Exclusive pH 7.5
A1AYR5	Pden_0295	Transcriptional regulator, MarR family	Unknown	Exclusive pH 7.5
A1AZ39	Pden_0419	Ribonuclease 3 (EC 3.1.26.3) (Ribonuclease III) (RNase III)	Cytoplasmic	Exclusive pH 7.5
A1AZJ1	Pden_0573	Uncharacterized protein	Cytoplasmic	Exclusive pH 7.5
A1AZS2	Pden_0654	Uncharacterized protein	Unknown	Exclusive pH 7.5
A1AZS4	Pden_0656	Phosphoadenylyl-sulfate reductase (thioredoxin) (EC 1.8.4.8)	Cytoplasmic	Exclusive pH 7.5
A1AZY3	Pden_0715	Urocanate hydratase (Urocanase) (EC 4.2.1.49) (Imidazolonepropionate hydrolase)	Cytoplasmic	Exclusive pH 7.5
A1B0E2	Pden_0875	tRNA 2-thiocytidine biosynthesis protein TtcA	Cytoplasmic	Exclusive pH 7.5
A1B0G2	Pden_0895	Release factor glutamine methyltransferase (RF MTase) (EC 2.1.1.297) (N5-glutamine methyltransferase PrmC) (Protein-(glutamine-N5) MTase PrmC) (Protein-glutamine N-methyltransferase PrmC)	Cytoplasmic	Exclusive pH 7.5
A1B1J3	Pden_1282	Shikimate kinase (SK) (EC 2.7.1.71)	Cytoplasmic	Exclusive pH 7.5
A1B1R0	Pden_1350	Transcriptional regulator, XRE family	Cytoplasmic	Exclusive pH 7.5
A1B1T4	Pden_1377	Redoxin domain protein	Unknown	Exclusive pH 7.5
A1B2P2	Pden_1689	Globin	Cytoplasmic	Exclusive pH 7.5
A1B389	Pden_1888	Phosphoribosyl-ATP pyrophosphatase (PRA-PH) (EC 3.6.1.31)	Cytoplasmic	Exclusive pH 7.5
A1B392	Pden_1891	Queuine tRNA-ribosyltransferase (EC 2.4.2.29) (Guanine insertion enzyme) (tRNA-guanine transglycosylase)	Cytoplasmic	Exclusive pH 7.5
A1B394	Pden_1893	Methionine-R-sulfoxide reductase (EC 1.8.4.11)	Cytoplasmic	Exclusive pH 7.5
A1B3E4	Pden_1945	UBA/THIF-type NAD/FAD binding protein	Cytoplasmic/Membrane	Exclusive pH 7.5
A1B3F3	Pden_1955	6-phosphogluconate dehydratase (EC 4.2.1.12)	Cytoplasmic	Exclusive pH 7.5
A1B3I1	Pden_1983	Uncharacterized protein	Cytoplasmic	Exclusive pH 7.5
A1B3U2	Pden_2094	Tetraacyldisaccharide 4'-kinase (EC 2.7.1.130) (Lipid A 4'-kinase)	Cytoplasmic	Exclusive pH 7.5
A1B3Z3	Pden_2145	DNA polymerase III, delta prime subunit (EC 2.7.7.7)	Cytoplasmic	Exclusive pH 7.5
A1B477	Pden_2229	Type III pantothenate kinase (EC 2.7.1.33) (PanK-III) (Pantothenic acid kinase)	Unknown	Exclusive pH 7.5
A1B4C2	Pden_2274	50S ribosomal protein L35	Unknown	Exclusive pH 7.5
A1B4Q9	Pden_2415	Transcriptional regulator, XRE family	Unknown	Exclusive pH 7.5

A1B4X0	Pden_2477	N-acetylglucosamine-6-phosphate deacetylase (EC 3.5.1.25)	Cytoplasmic	Exclusive pH 7.5
A1B531	Pden_2538	Precorrin-6y C5,15-methyltransferase (Decarboxylating), CbiE subunit (EC 2.1.1.132)	Cytoplasmic	Exclusive pH 7.5
A1B568	Pden_2575	Polynucleotide adenylyltransferase region	Cytoplasmic	Exclusive pH 7.5
A1B5I3	Pden_2690	Uncharacterized protein	Cytoplasmic/Membrane	Exclusive pH 7.5
A1B5M7	Pden_2737	Uncharacterized protein	Unknown	Exclusive pH 7.5
A1B5P4	Pden_2754	Short-chain dehydrogenase/reductase SDR	Cytoplasmic	Exclusive pH 7.5
A1B5Q6	Pden_2766	Secreted periplasmic protein	Unknown	Exclusive pH 7.5
A1B5U1	Pden_2801	tRNA pseudouridine synthase B (EC 5.4.99.25) (tRNA pseudouridine(55) synthase) (Psi55 synthase) (tRNA pseudouridylate synthase) (tRNA-uridine isomerase)	Cytoplasmic	Exclusive pH 7.5
A1B681	Pden_2941	Efflux transporter, RND family, MFP subunit	Cytoplasmic/Membrane	Exclusive pH 7.5
A1B682	Pden_2942	Transporter, hydrophobe/amphiphile efflux-1 (HAE1) family	Cytoplasmic/Membrane	Exclusive pH 7.5
A1B6C6	Pden_2987	Anaerobic ribonucleoside-triphosphate reductase activating protein	Cytoplasmic	Exclusive pH 7.5
A1B6M9	Pden_3092	Uncharacterized protein	Cytoplasmic	Exclusive pH 7.5
A1B717	Pden_3230	AMP-dependent synthetase and ligase	Cytoplasmic	Exclusive pH 7.5
A1B7C8	Pden_3349	Nucleoid protein H-NS	Unknown	Exclusive pH 7.5
A1B7Y1	Pden_3555	Uncharacterized protein	Cytoplasmic	Exclusive pH 7.5
A1B834	Pden_3611	Uncharacterized protein	Unknown	Exclusive pH 7.5
A1B841	Pden_3618	Glutathione S-transferase, C-terminal domain	Cytoplasmic	Exclusive pH 7.5
A1B875	Pden_3652	ABC transporter related protein	Cytoplasmic	Exclusive pH 7.5
A1B888	Pden_3665	CinA domain protein	Cytoplasmic	Exclusive pH 7.5
A1B8K6	Pden_3784	Cytidylate kinase (CK) (EC 2.7.4.25) (Cytidine monophosphate kinase) (CMP kinase)	Cytoplasmic	Exclusive pH 7.5
A1B8R3	Pden_3841	Helicase domain protein	Cytoplasmic	Exclusive pH 7.5
A1B8S8	Pden_3856	Substrate-binding region of ABC-type glycine betaine transport system	Periplasmic	Exclusive pH 7.5
A1B8T2	Pden_3860	NUDIX hydrolase	Cytoplasmic	Exclusive pH 7.5
A1B914	Pden_3942	Ubiquinone biosynthesis hydroxylase, UbiH/UbiF/VisC/COQ6 family	Cytoplasmic	Exclusive pH 7.5
A1B992	Pden_4020	ABC transporter related protein	Cytoplasmic/Membrane	Exclusive pH 7.5
A1B9J6	Pden_4126	TrkA-N domain protein	Cytoplasmic/Membrane	Exclusive pH 7.5

A1B9L0	Pden_4140	Periplasmic solute binding protein	Periplasmic	Exclusive pH 7.5
A1B9S0	Pden_4200	ABC transporter related protein	Cytoplasmic/Membrane	Exclusive pH 7.5
A1BA17	Pden_4297	GTP cyclohydrolase FolE2 (EC 3.5.4.16)	Cytoplasmic	Exclusive pH 7.5
A1BA38	Pden_4318	Cytochrome c oxidase assembly protein CtaG	Unknown	Exclusive pH 7.5
A1BA62	Pden_4342	Sulfate ABC transporter, inner membrane subunit CysW	Cytoplasmic/Membrane	Exclusive pH 7.5
A1BAF3	Pden_4433	Transcriptional regulator, IclR family	Cytoplasmic	Exclusive pH 7.5
A1BAX3	Pden_4604	Transcriptional regulator, IclR family	Cytoplasmic	Exclusive pH 7.5
A1BB27	Pden_4660	ABC transporter related protein	Cytoplasmic/Membrane	Exclusive pH 7.5
A1BB91	Pden_4724	GreA/GreB family elongation factor	Cytoplasmic	Exclusive pH 7.5
A1BC18	Pden_5002	Rhodanese domain protein	Unknown	Exclusive pH 7.5
A1BC22	Pden_5006	Aliphatic sulfonates family ABC transporter, periplasmic ligand-binding protein	Unknown	Exclusive pH 7.5
A1BC26	Pden_5010	ABC transporter related protein	Cytoplasmic/Membrane	Exclusive pH 7.5
A1BCE3	Pden_5127	Transcriptional regulator, Fis family	Unknown	Exclusive pH 7.5

[1]Protein annotated from UniProt (UP000000361). [2]Genes annotated from GeneBank (T00440). [3]Subcellular location according with PSOTb v3.0.2.[4]Fold change calculated as the ratio protein expression at pH 7.2/pH 7.5 (positive values indicate proteins over-represented at pH 7.2 and negative values correspond to proteins over-represented at pH 7.5). Proteins showing an absolute value of fold change >100 were considered exclusive at the indicated pH.

Table S4. Proteins involved in biosynthesis of cofactors affected by pH in *P. denitrificans.*

Protein ID[1]	Gene ID [2]	Protein name	Cofactor	Fold change[3]
A1B4M1	Pden_2375	GTP cyclohydrolase-2 (EC 2.5.4.25)	Riboflavin	Exclusive pH 7.2
A1B2B7	Pden_1561	Diaminohydroxyphosphoribosylaminopyrimidine deaminase (EC 2.5.4.26) (EC 1.1.1.193)	Riboflavin	Exclusive pH 7.2
A1B2B3	Pden_1557	Riboflavin synthase, alpha subunit	Riboflavin	Exclusive pH 7.2
A1BB21	Pden_4654	HAD superfamily (Subfamily IIIB) phosphatase, TIGR01672 (EC 3.1.3.2)	Riboflavin	Exclusive pH 7.2
A1B885	Pden_3662	Riboflavin biosynthesis protein	Riboflavin	Exclusive pH 7.2
A1BCD5	Pden_5119	NADPH-dependent FMN reductase (EC 1.5.1.38)	Riboflavin	Exclusive pH 7.2
A1B3Y8	Pden_2140	Cyclic pyranopterin monophosphate synthase accessory protein (molybdenum cofactor biosynthesis protein C) (EC 4.6.1.17)	Molybdenum cofactor	Exclusive pH 7.2
A1B4I7	Pden_2340	Molybdopterin synthase subunit MoaE (EC 2.8.1.12)	Molybdenum cofactor	Exclusive pH 7.2
A1B3Y9	Pden_2141	Molybdopterin molybdochelatase (EC 2.10.1.1)	Molybdenum cofactor	Exclusive pH 7.2
A1AY05	Pden_0032	Molybdenum cofactor guanylyltransferase (MoCo guanylyltransferase) (EC 2.7.7.77)	Molybdenum cofactor	Exclusive pH 7.2
A1B0B9	Pden_0851	Nicotinamidase (EC 3.5.1.19)	Nicotinamide adenine dinucleotide	Exclusive pH 7.2
A1B973	Pden_4001	Purine nucleosidase (EC 3.2.2.1)	Nicotinamide adenine dinucleotide	Exclusive pH 7.2
A1B1X5	Pden_1418	MazG family protein	Nicotinamide adenine dinucleotide	Exclusive pH 7.2
A1AZE2	Pden_0524	Purine nucleoside phosphorylase (EC 2.4.2.1) (inosine-guanosine phosphorylase)	Nicotinamide adenine dinucleotide	Exclusive pH 7.2
A1B5M1	Pden_2731	5'-nucleotidase SurE (EC 3.1.3.5) (nucleoside 5'-monophosphate phosphohydrolase)	Nicotinamide adenine dinucleotide	Exclusive pH 7.2
A1B1M9	Pden_1318	Probable nicotinate-nucleotide adenylyltransferase (EC 2.7.7.18)	Nicotinamide adenine dinucleotide	Exclusive pH 7.2
A1B0I8	Pden_0921	NAD kinase (EC 2.7.1.23) (ATP-dependent NAD kinase)	Nicotinamide adenine dinucleotide	Exclusive pH 7.2
A1BAF0	Pden_4430	Nicotinate-nucleotide pyrophosphorylase (carboxylating) (EC 2.4.2.19)	Nicotinamide adenine dinucleotide	Exclusive pH 7.2
A1BAF1	Pden_4431	L-aspartate oxidase (EC 1.4.3.16)	Nicotinamide adenine dinucleotide	Exclusive pH 7.2
A1B4H9	Pden_2332	Cobalamin (vitamin B12) biosynthesis CbiX protein	Cobalamin	Exclusive pH 7.2
A1B529	Pden_2536	Precorrin-3 methyltransferase	Cobalamin	Exclusive pH 7.2
A1B533	Pden_2540	Precorrin-4 C11-methyltransferase	Cobalamin	Exclusive pH 7.2
A1B535	Pden_2542	Precorrin-6A synthase	Cobalamin	Exclusive pH 7.2
A1B530	Pden_2537	Precorrin-6A reductase	Cobalamin	Exclusive pH 7.2
A1B525	Pden_2532	Cobaltochelatase CobN subunit	Cobalamin	Exclusive pH 7.2
A1B439	Pden_2191	Cobaltochelatase CobT subunit	Cobalamin	2.44
A1B653	Pden_2913	Cobyrinic acid a,c-diamide synthase	Cobalamin	Exclusive pH 7.2
A1B522	Pden_2529	Cob(I)yrinic acid a,c-diamide adenosyltransferase	Cobalamin	Exclusive pH 7.2
A1B3I2	Pden_1984	Dihydroxy-acid dehydratase (DAD) (EC 4.2.1.9)	Pantothenate/Coenzyme A	2.36
A1B595	Pden_2602	Branched-chain amino acid aminotransferase (EC 2.6.1.42)	Pantothenate/Coenzyme A	3.48
A1B8W4	Pden_3892	Ketopantoate hydroxymethyltransferase (EC 2.1.2.11)	Pantothenate/Coenzyme A	2.34
A1B2L1	Pden_1658	Pantothenate synthetase (PS) (EC 6.3.2.1) (pantoate-β-alanine ligase) (pantoate-activating enzyme)	Pantothenate/Coenzyme A	Exclusive pH 7.2
A1AXY4	Pden_0011	Non-canonical purine NTP pyrophosphatase (EC 3.6.1.9)	Pantothenate/Coenzyme A	Exclusive pH 7.2
A1B8X8	Pden_3906	3-hydroxyacyl-(acyl-carrier-protein) dehydratase FabZ	Biotin	Exclusive pH 7.2
A1B659	Pden_2919	Adenosylmethionine-8-amino-7-oxononanoate aminotransferase	Biotin	Exclusive pH 7.2
A1B658	Pden_2918	ATP-dependent dethiobiotin synthetase BioD	Biotin	Exclusive pH 7.2
A1B1Z0	Pden_1433	Biotin synthase 1	Biotin	Exclusive pH 7.2
A1B478	Pden_2230	Biotin-acetyl-CoA-carboxylase ligase	Biotin	Exclusive pH 7.2
A1B0G5	Pden_0898	4-hydroxythreonine-4-phosphate dehydrogenase	Pyridoxal phosphate	Exclusive pH 7.2
A1B0C6	Pden_0858	Pyridoxine/pyridoxamine 5'-phosphate oxidase (pyridoxal 5'-phosphate synthase)	Pyridoxal phosphate	Exclusive pH 7.2

[1]Protein annotated from UniProt (UP000000361). [2]Genes annotated from GeneBank (T00440). [3]Fold change calculated as the ratio of protein expression at pH 7.2/pH 6.5. Proteins exclusive at pH 7.2 showed an absolute value of fold change >100.

Table S5. Oligonucleotides used in the qRT-PCR analysis.

Gene ID[1]	Protein ID[2]	Protein Name	Sequence Forward (5'→3')	Tm
Pden_1433	A1B1Z0	Biotin synthase 1	CCACGCCCTGCCCTTCGCCGAT	73.4
Pden_2918	A1B658	ATP-dependent dethiobiotin synthetase BioD	CGCCTGCCCGCCTCGCCGCATC	74.4
Pden_1658	A1B2L1	Pantothenate synthetase	GTTGCTTGCCCCGCTGGGCGTCGA	72.1
Pden_3662	A1B885	Riboflavin biosynthesis protein	GACGCGCCCCTGGGCGTCATCACC	71.8
Pden_2141	A1B3Y9	Molybdopterin molybdochelatase	GGATGCGCTCGGCCGCGCCATGAT	72.8
Pden_0851	A1B0B9	Nicotinamidase	CGCAGGACTGGCACCCCCACGACC	71.7
Pden_2731	A1B5M1	5'-nucleotidase SurE	TCTGGACCGTCGCCCCGGCCTT	73.8
Pden_2529a	A1B522	Cob(I)yrinic acid a,c-diamide adenosyltransferase	ACCGGCAAGGGCAAGGGCAAGTCC	71.9
Pden_2834	A1B5X4	DNA polymerase III β-subunit DnaN	CATGTCGTGGGTCAGCATAC	71.3
**Locus**	**Protein ID**	**Protein Name**	**Sequence Reverse (5'→3')**	**Tm**
Pden_1433	A1B1Z0	Biotin synthase 1	ATCCTCAGGGCAGCCGCCGGTCT	74.4
Pden_2918	A1B658	ATP-dependent dethiobiotin synthetase BioD	CCCGCCCGCGCCCTCGACCAC	74.1
Pden_1658	A1B2L1	Pantothenate synthetase	GTTGCTTGCCCCGCTGGGCGTCGA	72.1
Pden_3662	A1B885	Riboflavin biosynthesis protein	CCGGTTCGCCCGCGACTCCACGTT	73.2
Pden_2141	A1B3Y9	Molybdopterin molybdochelatase	GCCGAGCGCAGGGCATAGCCGTCC	72.4
Pden_0851	A1B0B9	Nicotinamidase	GCCCGCGCCATCGGTCCCGATCAC	71.7
Pden_2731	A1B5M1	5'-nucleotidase SurE	CGCGGCCCCAGCTCGGCGATCAG	73.3
Pden_2529a	A1B522	Cob(I)yrinic acid a,c-diamide adenosyltransferase	GCAACCGCCGCTCGCCGGTATCCC	72.3
Pden_2834	A1B5X4	DNA polymerase III β-subunit DnaN	CTCGCGACCATGCATATAGA	71.5

[1]Genes annotated from GeneBank (T00440). [2]Protein annotated from UniProt (UP000000361).
